# Oxygen-Donor Metalloligands Induce Slow Magnetization Relaxation in Zero Field for a Cobalt(II) Complex with {CoO_4_} Motif

**DOI:** 10.1021/acs.inorgchem.4c00054

**Published:** 2024-03-12

**Authors:** Giuseppe Lococciolo, Sandeep K. Gupta, Sebastian Dechert, Serhiy Demeshko, Carole Duboc, Mihail Atanasov, Frank Neese, Franc Meyer

**Affiliations:** † Institute of Inorganic Chemistry, University of Göttingen, Tammannstraße 4, Göttingen 37077, Germany; ‡ Université Grenoble Alpes, CNRS UMR 5250, DCM, Grenoble F-38000, France; § Max-Planck-Institut für Kohlenforschung Kaiser-Wilhelm-Platz 1, Mülheim an der Ruhr 45470, Germany; ∥ Institute of General and Inorganic Chemistry, Bulgarian Academy of Sciences, Akad. Georgi Bontchev Street 11, Sofia 1113, Bulgaria

## Abstract

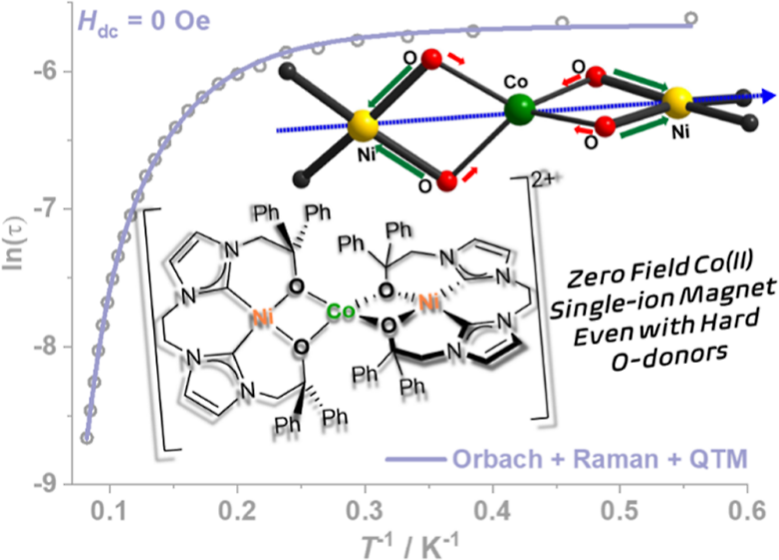

Most 3d metal-based single-molecule magnets (SMMs) use N-ligands or ligands with even softer donors to impart a particular coordination geometry and increase the zero-field splitting parameter |*D*|, while complexes with hard O-donor ligands showing slow magnetization relaxation are rare. Here, we report that a diamagnetic Ni^II^ complex of a tetradentate ligand featuring two *N*-heterocyclic carbene and two alkoxide-O donors, [L^O,O^Ni], can serve as a {O,O′}-chelating metalloligand to give a trinuclear complex [(L^O,O^Ni)Co(L^O,O^Ni)](OTf)_2_ (**2**) with an elongated tetrahedral {Co^II^O_4_} core, *D* = −74.3 cm^–1^, and a spin reversal barrier *U*_eff_ = 86.9 cm^–1^ in the absence of an external dc field. The influence of diamagnetic Ni^II^ on the electronic structure of the {CoO_4_} unit in comparison to [Co(OPh)_4_]^2–^ (**A**) has been probed with multireference ab initio calculations. These reveal a contrapolarizing effect of the Ni^II^, which forms stronger metal–alkoxide bonds than the central Co^II^, inducing a change in ligand field splitting and a 5-fold increase in the magnetic anisotropy in **2** compared to **A**, with an easy magnetization axis along the Ni–Co–Ni vector. This demonstrates a strategy to enhance the SMM properties of 3d metal complexes with hard O-donors by modulating the ligand field character via the coordination of diamagnetic ions and the benefit of robust metalloligands in that regard.

## Abstract 2

The Ni^II^ complex of a new tetradentate ligand with two carbene-C and two alkoxide-O donors is shown to serve as an {O,O′}-chelating metalloligand, and its weak ligand field with the contrapolarizing effect of the second-sphere Ni^II^ ions enhances the magnetic anisotropy at the central {Co^II^O_4_} site in a trinuclear NiCoNi complex, giving *D* = −74.3 cm^−1^ and a spin reversal barrier of 86.9 cm^−1^ in the absence of an external dc field.

## Introduction

The advent of classical magnet-like behavior in single-molecule magnets (SMMs) that also exhibited quantum phenomena promised a potential alternative for technological applications in the areas of high-density data storage, quantum computing, spintronics, and others.^1−6^ However, slow relaxation of magnetization in SMMs was initially observable only at very low temperatures. Various design strategies were pursued to enhance the magnetic anisotropy of such paramagnetic complexes, which would mean that the spin reversal within the bistable ground state occurs only at higher temperatures and over a longer time.^7−10^ As the earlier strategies to synthesize polynuclear complexes with a very large ground state spin (*S*) were unsuccessful with respect to maximizing the anisotropy,^11−13^ researchers focused more on maximizing the axial zero-field splitting (ZFS) parameter *D* in mononuclear single-ion magnets (SIMs), as the overall effective energy barrier for 3d metal ion-based SMMs is dictated by *U*_eff_ = |*D*|*S*^2^ (for integer spins) or *U*_eff_ = |*D*| (*S*^2^ – 1/4) (for half-integer spins). Since spin–orbit coupling (SOC) in 3d metal ions is intrinsically rather small, new synthetic design strategies had to be developed. In that respect, low coordination numbers are usually beneficial in order to induce a larger energy gap between the ground and lowest excited magnetic states, leading to an increase in the effective demagnetization barrier. This was exemplified by linear 3d metal complexes such as the Fe^I^ complex {Fe[C(SiMe_3_)_3_]_2_}^−^ or the Co^II^ complex {Co[C(SiMe_2_ONaph)_3_]_2_}, the latter exhibiting a limiting case of magnetic anisotropy for a Co^II^ ion.^14−17^

Among the 3d transition metal ions, high-spin Co^II^, which is a *d*^7^ Kramers ion with a non-integer spin (*S* = 3/2), has been the preferred choice for designing 3d metal-based SIMs, especially with linear two-coordinate (*C*_∞*v*_), planar three-coordinate, and tetragonally elongated four-coordinate (*D*_2*d*_) coordination geometries; the latter are probably the most studied, and a wide variety of different ligand systems has been employed, in particular, bidentate-chelating ligands.^7−10,18^ As Co^II^ ions in a distorted tetrahedral coordination environment lack first-order orbital angular momentum, magnetic anisotropy results from the contribution of second-order angular momentum by mixing the ground state with the excited states via SOC. The magnitude of *D* depends on the energy of close-lying excited states, and the sign of *D* is determined by the orbital ordering resulting from the ligand field splitting.^18^ It is generally assumed that weak ligand fields imparted by soft donor atoms are beneficial for achieving the sought-after small energetic separation between two unevenly populated orbitals.^7,10^ Such near degeneracy of the d_*x*^2^–*y*^2^_ and d_*xy*_ orbitals has been evidenced for elongated tetrahedral (*T*_*d*_ → *D*_2*d*_) Co^II^ complexes, leading to rather large and negative *D*.^19−30^

As the ordering and splitting of the 3d orbitals imparted by the ligand field largely depend on the nature of the ligand donor atoms, structural metrics, and the symmetry of the coordination polyhedra, these factors play the main role in dictating the effect of SOC and hence the sign and magnitude of the ZFS.^7−10,19−22,31−40^ Apart from these factors, handles such as the nature of the chemical bonding, rigidity of the local coordination environment, magnetic exchange, the second coordination sphere, and the nature of the counteranions are key to manipulating the magnetic anisotropy of SMMs.^34,36,41−43^ In recent years, various magneto-structural correlation studies have unraveled the influence of metal–ligand covalent bonding, the nature of the donor atoms, and structural distortion in four-coordinate Co^II^ systems, providing guidelines for tailoring their electronic structure in order to enhance the resulting magnetic properties.^7−10,19−22,25,30−41^ It should be noted though that the presence of large negative *D* is not the only crucial factor in determining whether a complex will exhibit slow relaxation dynamics, and it does not always translate into a large *U*_eff_ due to additional underbarrier relaxation processes such as quantum tunneling of magnetization (QTM), which is a crucial deactivating factor preventing SIM behavior.

**Figure 1:**
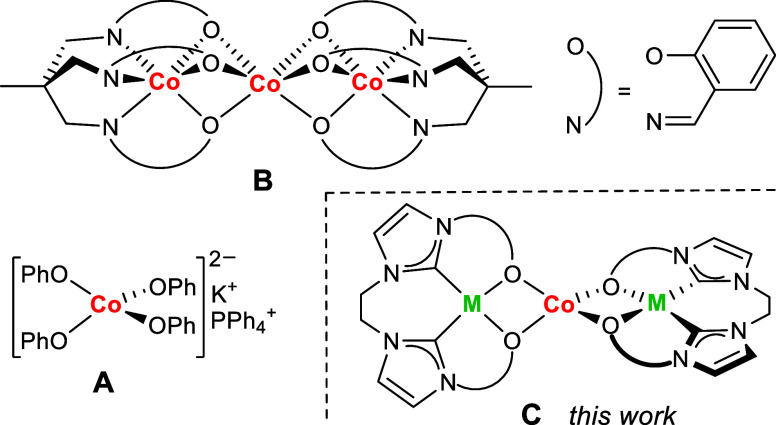
Previously reported O–ligated Co^II^ complexes **A**(22) and **B**(46) showing slow relaxation of the magnetization and generic structure **C** of the complexes reported in this work.

Slow magnetic relaxation in the absence of an applied magnetic field was reported for the four-coordinate tetrathiolato Co^II^ complex [Co(SPh)_4_](PPh_4_)_2_ as one of the earliest examples.^44^ It was found that *D* increased as the ligand field strength decreased across the series of complexes [Co(EPh)_4_](PPh_4_)_2_ with E = O, S, Se,^22^ and *D* was predicted to be the largest in the hypothetical [Co(TePh)_4_]^2–^ complex due to the π-anisotropy of the ligand donor atoms (Te).^36^ These findings are in line with the idea that ligands with softer donor atoms raise the value of |*D*|. Not surprisingly, the years since then have seen few studies on SIMs based on 3d metal ions ligated solely by hard O-donor ligands, and very few such O–ligated Co^II^ complexes have been shown to exhibit slow relaxation of the magnetization in the absence of an external applied magnetic field (see Table S8 for reference).^7,45^ For complex [Co(OPh)_4_]K(Ph_4_P) (**A**, Figure 1), slow relaxation was only observed in the case of a 6% magnetically diluted sample in the analogous Zn^II^ complex as the matrix, with an energy barrier of 34 cm^–1^.^22^ Most recently, the Co^II^_3_ complex {[Co(μ-L)]_2_Co} {**B**, where H_3_L = 1,1,1-tris[(salicylideneamino)methyl]ethane}, which has a central elongated trigonal prismatic {CoO_6_} core, was reported to behave as a SMM with open hysteresis at zero field. In this case, the relatively strong magnetic exchange coupling in the Co_3_ string, in combination with the collinearity of the local anisotropy axes, was shown to be responsible for the suppression of QTM and promote SMM behavior.^46^

In the present work, we pursued the strategy used for **B**, viz., the use of metalloligands providing alkoxides as O-donors for coordination of a central Co^II^ ion, but we aimed at a novel system featuring diamagnetic bidentate {O,O′}-chelating metalloligands that give rise to a magnetically isolated and tetragonally elongated tetrahedral {CoO_4_} core of *D*_2*d*_ symmetry. To ensure the diamagnetic (*S* = 0) character of the metalloligands and build upon our previous expertise in functionalized *N*-heterocyclic carbene (NHC) ligand scaffolds,^47−49^ we envisioned subunits with peripheral strong field NHC donors and bridging alkoxides, resulting in trinuclear M–Co–M systems, as shown in Figure 1**C**. The {O,O′}-chelating metalloligands were expected to serve several beneficial purposes. First, their relatively large size (compared to usual bidentate ligands) combined with the diamagnetic character of M increases the separation between paramagnetic Co^II^ centers in neighboring molecules in the crystal lattice, thus decreasing intermolecular dipolar interactions and quenching a potential QTM pathway. Second, the presence of a diamagnetic ion has been shown to have a pronounced effect on the quenching of QTM via charge polarization, as was demonstrated for 3d–4f ion heterometallic SMMs.^50,51^ For example, the coordination of diamagnetic metal ions like Zn^II^ to 4f metal-bound phenolates, resulting in O-bridging motifs, was shown to induce larger charge polarization and to increase the negative charges on the phenolato-O atoms that eventually govern the orientation of the easy axis.^50,51^ Third, bridging to the second metal ion M should reduce the ligand field strength of the O-donors bound to Co^II^ compared to terminal alkoxides. Lastly, the square-planar motif with a heavy metal ion M in the {O, O′}-chelating metalloligands provides a stable and rather rigid coordination environment for the Co^II^ center, thereby reducing potential pathways for relaxation via vibronic coupling. Here, we present the synthesis of the new NHC-based metalloligands with M being Ni^II^, as well as the comprehensive experimental and computational investigation of a Ni–Co–Ni type **C** complex, as shown in Figure 1, featuring a {CoO_4_} core. We show that this system indeed exhibits slow relaxation of the magnetization in the absence of an external magnetic field, thus demonstrating a promising strategy toward 3d metal ion-based SIMs with O-donor ligation.

## Results and Discussion

### Nickel(II)-Based O,O′-Metalloligand

**Scheme 1:**
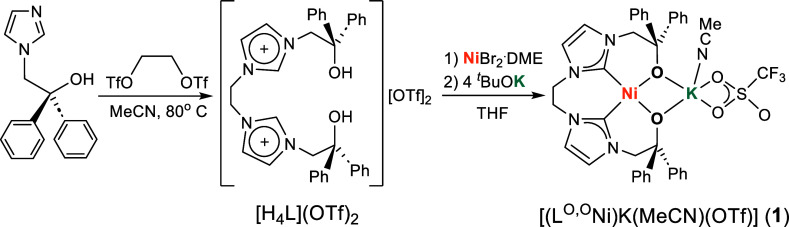
Synthesis of the K^+^-Salt of the Metalloligand **1**

The proligand [H_4_L^O,O^](OTf)_2_ was prepared by reacting 2-(1H-imidazole-1-yl)-1,1-diphenylethan-1-ol^52^ with 2 equivalents of 1,2-bis(trifluoromethylsulfonyloxyl)ethane^53^ in acetonitrile (Scheme 1). Sterically demanding substituents were installed next to the hydroxy groups in order to enforce a (distorted) tetrahedral ligation of the central Co^II^ ion in type **C** complexes. [H_4_L^O,O^](OTf)_2_ was characterized by NMR spectroscopy and MALDI mass spectrometry, the latter showing a major peak signal for the ion [H_4_L^O,O^(OTf)]^+^ at *m*/*z* = 705.4 (Figure S3). The reaction of [H_4_L^O,O^](OTf)_2_ with NiBr_2_·DME in THF in the presence of 4 equiv of KO^*t*^Bu, the latter serving as a base for deprotonation of both the imidazolium and hydroxy units of the proligand, provided the desired neutral Ni^II^ complex [L^O,O^Ni], which could be crystallized as the light yellow KOTf adduct [(L^O,O^Ni)K(MeCN)(OTf)] (**1**) after diffusion of Et_2_O into a MeCN solution of the product (Scheme 1).

**Figure 2:**
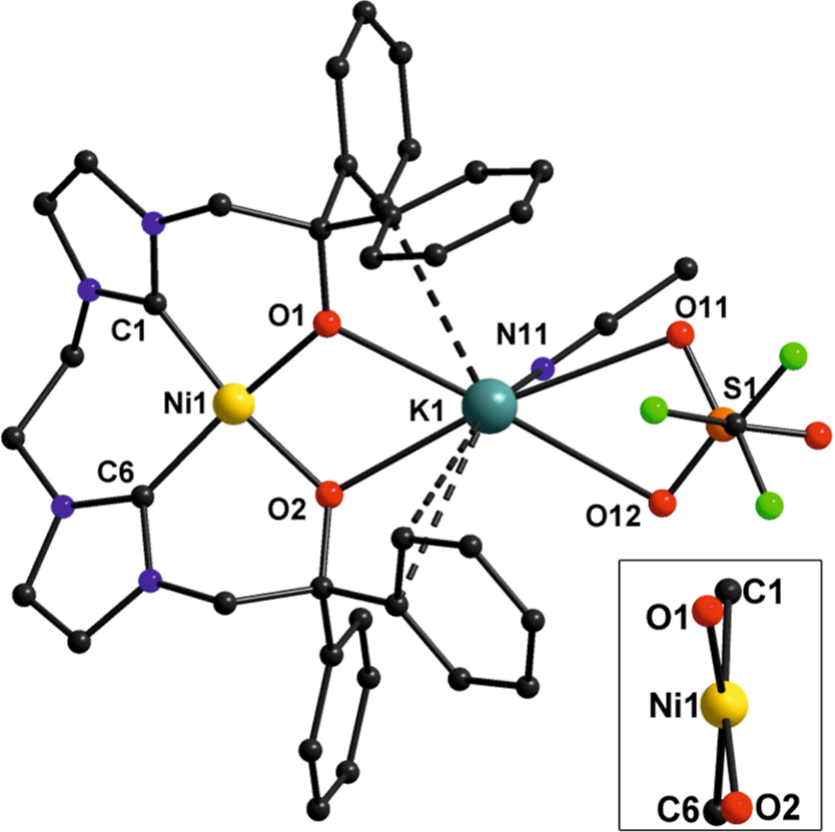
Molecular structure of complex **1**. Hydrogen atoms have been omitted for the sake of clarity.

Compound **1** crystallizes in the monoclinic space group *P*2_1_/*c* with the Ni^II^ ion in a slightly distorted square planar coordination environment (τ_4_ = 0.14)^54^ composed of the two carbene-C and alkoxido-O atoms of the tetradentate ligand [L^O,O^]^2–^, as anticipated (Figure 2). Selected structural parameters for **1** are listed in Table S2. All Ni–C/O bond lengths lie in the narrow range 1.8462(15)–1.8862(16) Å, with bond angles involving the central Ni^II^ and neighboring donor atoms in the range 82.92(5)–95.38(7)°. The K^+^ ion is associated with the two Ni-bound O atoms [Ni–O–K angles of 110.01(5) and 108.02(5)°] and further ligated by the O,O′-chelating triflate as well as a MeCN solvent ligand. Relatively close-lying parts of the flanking phenyl groups [shortest K–C^Ph^ distances 3.0891(18)–3.5235(16) Å] may indicate some additional K^+^···π interactions (Figure 2).

Association of the complex [L^O,O^Ni] with K^+^ in solid **1** evidences its propensity to serve as a chelating O,O′-metalloligand. However, the ESI(+) mass spectrum of a solution of **1** in MeCN shows a major peak at *m*/*z* = 611.3 for the ion [L^O,O^Ni + H]^+^ (Figure S8), suggesting that K^+^ largely dissociates in solution. ^1^H and ^13^C NMR spectra of **1** in MeCN-*d*_3_ reflect the diamagnetic character of the complex [L^O,O^Ni] with its Ni^II^ ion in a square planar environment, as anticipated for strong field NHC ligation, and the spectra are in accordance with *C*_2*v*_ symmetry (Figures S6 and S7).

### Synthesis and Structural Characterization of the NiCoNi Complex **2**

**Scheme 2:**
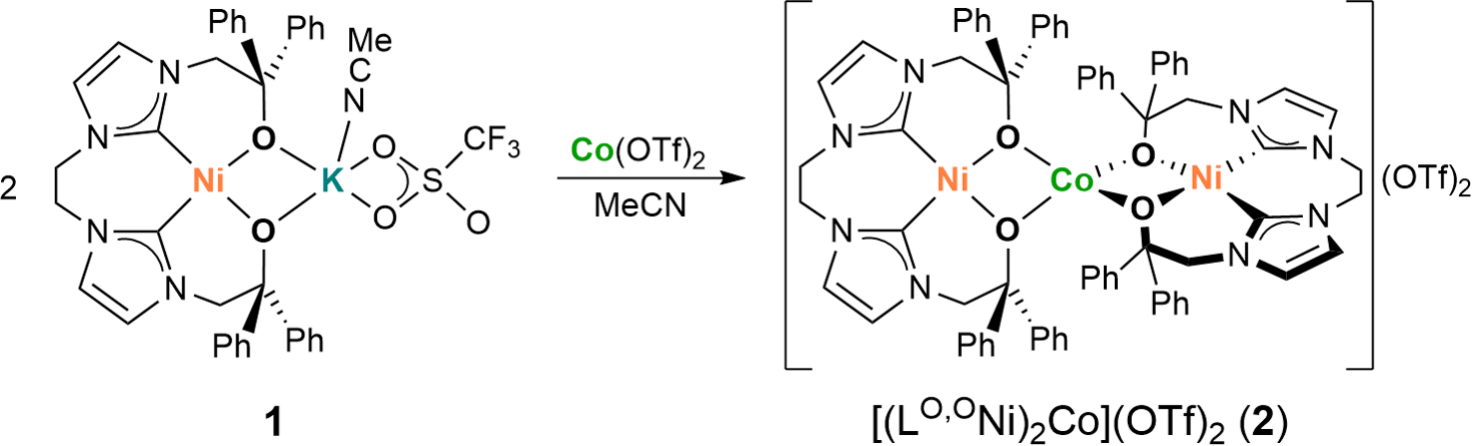
Synthesis of Trimetallic Complex **2**

The reaction of **1** with 0.5 equiv of anhydrous Co(OTf)_2_ leads to the trimetallic complex [(L^O,O^Ni)_2_Co](OTf)_2_ (**2**; Scheme 2), as evidenced by the ESI(+) mass spectrum showing a single major peak for the dication [(L^O,O^Ni)_2_Co]^2+^ at *m*/*z* = 640.6 (Figure S11), which also indicates the stability of the trinuclear core structure in solution. Diffusion of Et_2_O into an acetone solution of **2** gave reddish-green single crystals (monoclinic space group *P*2_1_/*c*) that were subjected to an X-ray diffraction analysis.

**Figure 3:**
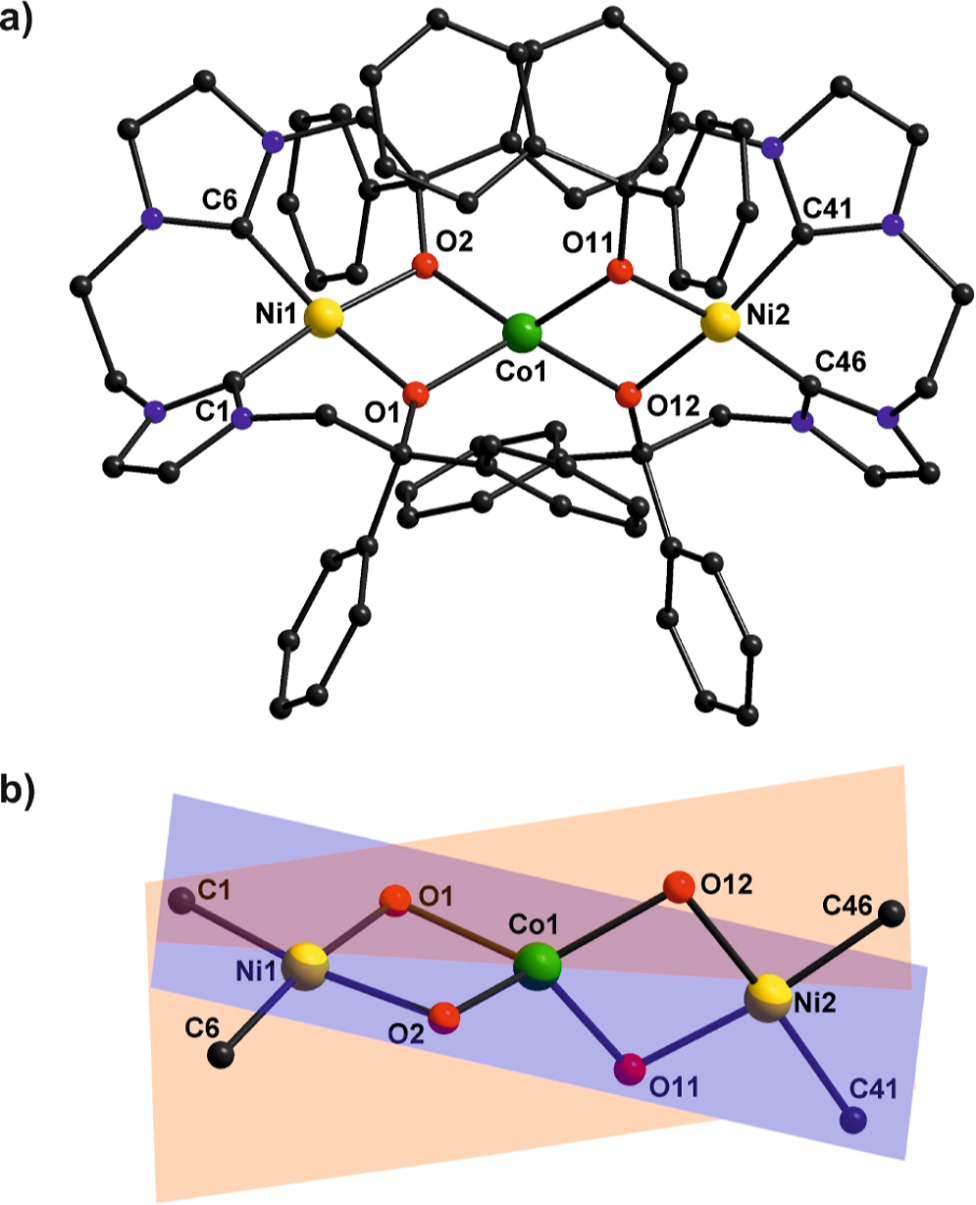
(a) Molecular structure of the cation of **2** with two [L^O,O^Ni] metalloligands providing an elongated tetrahedral coordination environment for the central Co^II^ ion; hydrogen atoms, counteranions, and lattice solvent molecules are omitted for clarity. (b) Core structure of **2** illustrating the nearly orthogonal arrangement of planes defined by the {O,O′}-chelating [L^O,O^Ni] metalloligands.

The molecular structure of the dication of **2** (Figure 3) confirms that two [L^O,O^Ni] units act as bidentate O,O′-chelating metalloligands to provide an {O_4_} donor environment for the central Co^II^ ion that is found in distorted (elongated) tetrahedral geometry (*D*_2*d*_; τ_4_ = 0.72). Structural features of the two [L^O,O^Ni] fragments in **2** are quite similar to those in **1** (Tables S4 and S5), with the Ni^II^ ions adopting a slightly distorted square planar configuration [τ_4_ = 0.13 (Ni_1_) and 0.15 (Ni_2_)] but Ni–O bond lengths are elongated [1.923(3)/1.953(3) Å in **2** versus 1.8733(11)–1.8843(11) Å in **1**]. Selected bond distances and bond angles are compiled in Table S3. Co–O bond lengths in **2** lie in a narrow range of 1.989(3)–1.996(3) Å with two acute O1–Co1–O2 and O11–Co1–O12 bite angles (2θ) of 79.58(11) and 79.69(11)°, respectively, imposed by the [L^O,O^Ni] metalloligands, and much wider O–Co–O angles involving the O-donor atoms from the two different [L^O,O^Ni] fragments [in the range 121.62(11)–130.02(13)°]. The 2θ angles are within the regime of 71–81° that was found optimal for harnessing large magnetic anisotropy in Co^II^ complexes with distorted tetrahedral {CoN_4_} cores, as derived from recent magnetostructural correlation studies.^19,25,40^ Furthermore, the dihedral angle (δ) of 84.32° between the two O–Co–O chelate planes defined by the [L^O,O^Ni] metalloligands (Figure 3b) is close to the ideal dihedral angle (90°) that was recently shown to lead to maximized magnetic anisotropy for a *D*_2*d*_ type {CoN_4_} system.^30^ These combined structural features suggest that **2** may exhibit a large magnetic anisotropy and favorable spin relaxation properties.

The UV–vis spectra recorded for MeCN solutions show that the proligand [H_4_L](OTf)_2_ absorbs only at high energy [λ_max_ around 260 nm (38460 cm^–1^) with a hump around 280 nm (35715 cm^–1^)] due to π–π* transitions in the ligand backbone (Figure S5). For **1**, these strong absorptions are red-shifted with λ_max_ around 320 nm (31250 cm^–1^, ε = 18.7 × 10^3^ M^–1^ cm^–1^), and a shoulder is discernible around 380 nm (26315 cm^–1^, ε = 2.5 × 10^3^ M^–1^ cm^–1^) and is tentatively attributed to a ligand-to-metal charge transfer (LMCT) transition (Figure S10). The latter gives rise to the light yellow color of **1**, while the 400–800 nm (25000–12500 cm^–1^) region is featureless. The UV–vis spectrum of complex **2** shows two major bands in the visible region at 410 nm (24390 cm^–1^, ε = 740 M^–1^ cm^–1^) and 567 nm (17640 cm^–1^, ε = 330 M^–1^ cm^–1^). The latter results from the d–d transitions of the high-spin d^7^ Co^II^ ion and is accompanied by two shoulder bands at 523 nm (19120 cm^–1^, ε = 280 M^–1^ cm^–1^) and 585 nm (17100 cm^–1^, ε = 295 M^–1^ cm^–1^) due to the distortion of the Co^II^ coordination sphere away from tetrahedral *T*_*d*_ symmetry toward *D*_2*d*_;^26,30^ band assignment is corroborated computationally (vide infra). A UV–vis spectrum of solid material of **2** (diffuse reflectance; Figure S14) exhibits similar features as observed for **2** in MeCN, confirming that the structure of the complex is preserved in solution.

### Magnetic Studies

**Figure 4:**
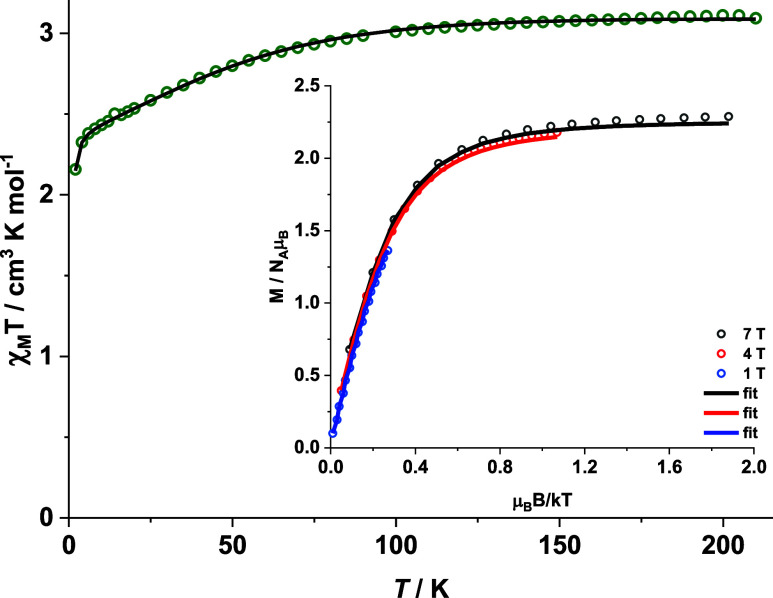
(a) Temperature dependence of χ_M_*T* for complex **2** measured under an applied dc field of 0.5 T. Inset: variable-temperature variable-field magnetization data for complex **2**. The solid lines are the best fit, with *D* = −74.3 cm^–1^, *g*_*x*_ = *g*_*y*_ = 2.33, and *g*_*z*_ = 2.89 (see Figure S26 for a comparison between experimental and CASSCF/NEVPT2 computed values).

The direct current (dc) magnetic properties of powdered polycrystalline **2** were probed with a Quantum Design MPMS3 SQUID magnetometer between 2 and 210 K; the upper temperature was determined by the pour point of the inert oil used to prevent the microcrystals from orienting in the magnetic field. The χ_M_*T* value of 3.09 cm^3^ mol^–1^ K at 210 K is higher than the expected spin-only value (1.875 cm^3^ mol^–1^ K) for an isolated noninteracting high-spin Co^II^ ion and two diamagnetic low-spin Ni^II^ ions, indicating considerable orbital contributions to the magnetic moment. The χ_M_*T* value remains almost constant until 100 K before gradually decreasing to 2.16 cm^3^ mol^–1^ K upon cooling to 2.0 K (Figure 4). As the shortest intermolecular Co^II^···Co^II^ distance in the crystal lattice is rather large (12.18 Å), this decrease of χ_M_*T* can be ascribed to the presence of large ZFS. The magnetic susceptibility data of complex **2** were fitted along with the variable-field variable-temperature (VTVH) magnetization data (Figure 4 inset) to the spin Hamiltonian (eq 1)
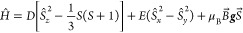
1where *D* and *E* represent the axial and the rhombic ZFS parameters; *S*, *S*_*x*_, *S*_*y*_, and *S*_*z*_ represent the total spin and its corresponding *x*, *y*, and *z* components; μ_B_, ***g***, and *B* represent the Bohr magneton, the *g*-tensor, and the magnetic flux density, respectively. The best fit using the julX_2s program^55^ yields *D* = −74.3 cm^–1^, *E*/*D* = 0, *g*_*x*_ = *g*_*y*_ = 2.33, *g*_*z*_ = 2.89 as well as some temperature-independent paramagnetism (TIP = 180 × 10^–6^ cm^3^ mol^–1^, subtracted). The large negative *D* indicates a significant separation between the ground state *M*_S_ = ±3/2 and the excited state *M*_S_ = ±1/2 Kramers doublets (KDs).

**Figure 5:**
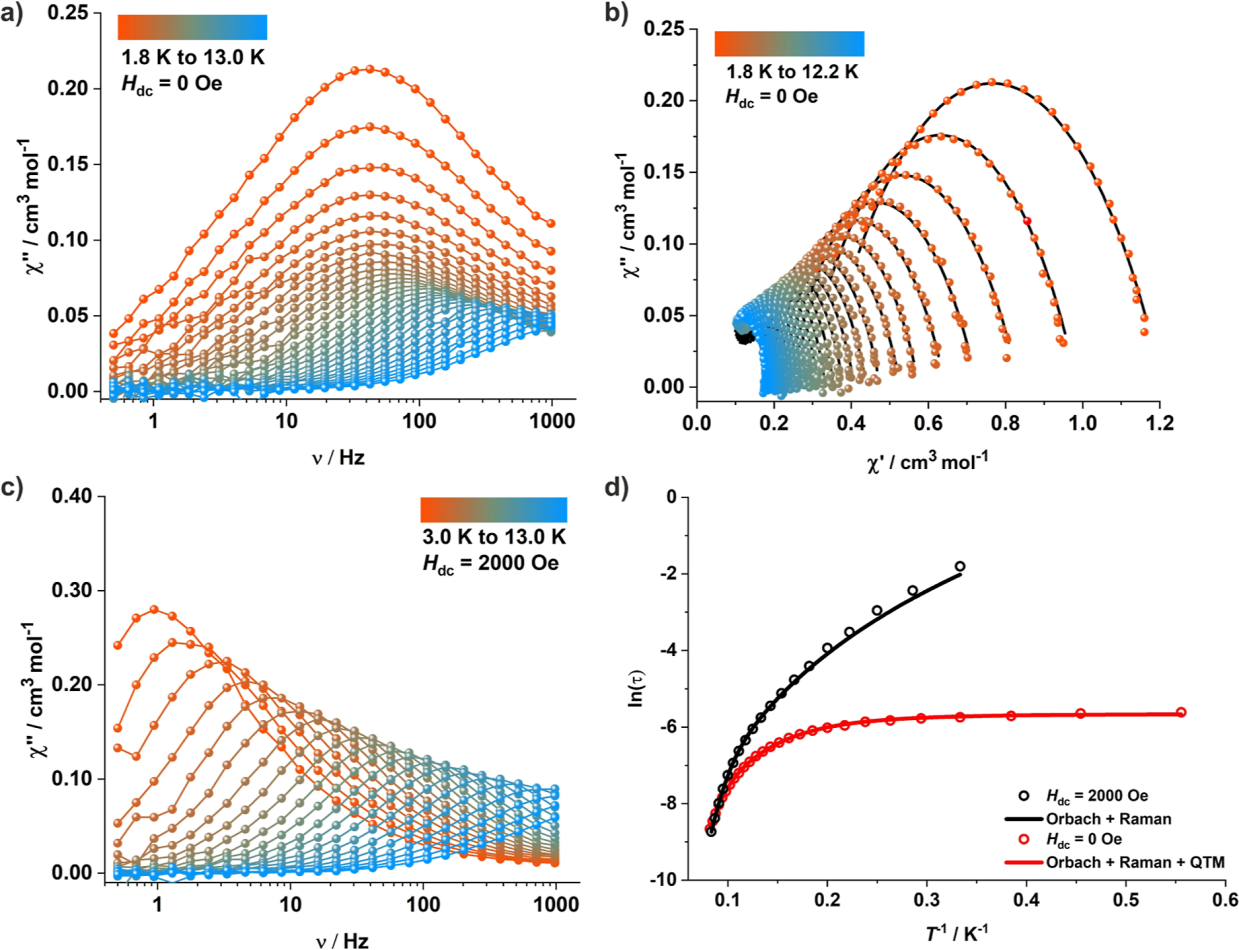
(a,c) Out-of-phase (χ_M_″) component of the frequency-dependent (0.1–1000 Hz) ac susceptibility for **2** measured in an oscillating ac field of 3.0 Oe under a zero dc field (a) and applied dc field of 2000 Oe (c), respectively. (b) Cole–Cole plots for **2** under a zero dc field. (d) Arrhenius plot of the relaxation time, ln (τ) vs *T*^–1^; the solid red and black lines represent the best fit to the data using a combination of relaxation mechanisms as indicated in the figure under the zero dc field and applied dc field of 2000 Oe, respectively.

To investigate the relaxation dynamics of magnetization, alternating current (ac) susceptibility measurements were performed on polycrystalline samples of **2** in an oscillating ac field of 3.0 Oe (frequency range 0.1–1000 Hz) without the application of any external dc field. Clear temperature-dependent and temperature-independent regimes were observed in the frequency-dependent out-of-phase (χ_M_″) component of the ac susceptibilities, with maxima observable up to 12.2 K (Figure 5a). Cole–Cole plots of the out-of-phase (χ_M_″) versus in-phase (χ_M_′) components of the ac susceptibilities display a distorted semicircular curve (Figure 5b). The χ_M_″ versus χ_M_′ curves were fitted with the generalized Debye function to extract the relaxation times, τ (Table S6);^56^ resulting α parameters in the 0.13–0.43 range indicate a wide distribution of relaxation times. To gain further insights into the dynamics of magnetic relaxation, the relaxation rates (τ^–1^) over the entire temperature range were analyzed with the following function
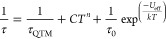
2where the first term represents magnetic relaxation through QTM, the second term corresponds to relaxation via the Raman process, and the last term represents relaxation through the Orbach mechanism. At low temperatures, QTM dominates the relaxation mechanism, whereas the Orbach and Raman processes seem to be favorable pathways for relaxation at higher temperatures (Figure 5d). The best fit yields parameters *U*_eff_ = 86.9 cm^–1^, τ_0_ = 1.32 × 10^–8^ s; *C* = 0.403 s^–1^ K^–*n*^, *n* = 3.54; τ_QTM_ = 0.00349 s. To the best of our knowledge, this is the highest energy barrier reported for any 3d metal SIM featuring solely O-donors around metal ions, i.e., with a {MO_*x*_} motif (see Table S8 for a compilation of relevant systems and references). *U*_eff_ = 43.8 cm^–1^ has been determined for the tricobalt(II) complex **B** with a central {CoO_6_} core, much smaller than the barrier for the local Co^II^ ions predicted from their large negative *D* values; this has been attributed to Orbach relaxation through the first or second excited states, possibly complemented by Raman relaxation. The first example of an exclusively O-donor-ligated complex showing slow magnetization relaxation at zero field was a 6% magnetically diluted sample of K(Ph_4_P)[Co(OPh)_4_], for which an energy barrier *U*_eff_ = 34 cm^–1^ was reported (see Table S8).^45^

In order to get further insights into the relaxation dynamics, ac susceptibility measurements were also carried out under the application of an external dc field (Figure S19). The optimum dc field was found to be 2000 Oe, which quenches the fast relaxation process via QTM that is operative in the lower temperature range; this is also evident from the disappearance of temperature-independent regimes in the frequency-dependent out-of-phase (χ_M_″) component of the ac susceptibilities (Figure 5c). Analysis of the Cole–Cole plots reveals α parameters in the 0.10–0.19 range, evidencing a narrower distribution of relaxation times in the presence of the external dc field (Figure S19 and Table S7). Again, fitting the relaxation times over the entire temperature range indicates the relaxation to mainly proceed via the Orbach and Raman relaxation mechanisms; the best fit parameters are *U*_eff_ = 93.1 cm^–1^, τ_0_ = 3.40 × 10^–9^ s; *C* = 0.088 s^–1^ K^–*n*^, *n* = 4.05 (Figure 5d). However, variable-field magnetization measurements conducted at 1.8 K at a sweep rate of 100 Oe/s did not reveal any opening of a hysteresis loop, suggesting that the blocking temperature of the SIM is below 1.8 K, viz., the minimum temperature accessible by the used SQUID magnetometer (Figure S20).

### Theoretical Calculations and Analysis

Spin-unrestricted DFT geometry optimizations were performed for **2** with all possible *S*_T_ values of 1/2, 3/2, 5/2, and 7/2 without restraining the spin density. In these calculations, a truncated model complex **2′** (Figure S21) was considered, where all eight phenyl substituents were replaced by hydrogen atoms. Löwdin spin-populations have been used to assign the local spins to metal centers Ni_1_, Co, and Ni_2_. In agreement with experimental magnetic data, a spin distribution with *M*_s_(Ni) = 0 for both Ni^II^ ions and *M*_s_(Co) = 3/2 turned out to be energetically most preferred compared to the three other alternatives (see Table 1). The *S*_T_ = 5/2 state represented by the *M*_s_(Ni^II^_1_) = 0, *M*_s_(Co^II^) = 3/2, and *M*_s_(Ni^II^_2_) = 1 spin configuration or alternatively *M*_s_(Ni^II^_1_) = 1, *M*_s_(Co^II^) = 3/2, and *M*_s_(Ni^II^_2_) = 0 spin configuration lies higher in energy by 17.7 kcal/mol. For the *S*_T_ = 3/2 ground state, the computed local geometrical parameters around the metal ions (Ni–O, Ni–C, and Co–O bond distances, the O–Co–O bite angle, and the Ni–O–Co bridging angles) are in good agreement with the X-ray crystallographic data (Table 1). Geometry optimization of **2** without any truncation was then performed using coordinates obtained from the X-ray crystallographic structure determination, followed by analytical frequency calculations (see Supporting Information for details on the computations and the computed IR spectrum). The computed vibrational frequencies turned out to be all positive, indicating that the computed geometry is at a stable minimum of the ground-state potential surface.

**Table 1:** Energies, Spin-Populations, and Geometric Parameters for the Truncated {NiCoNi} Model Complex **2′** (Figure S21) from Spin-Unrestricted DFT Calculations and Possible Spin Configurations (*M*_s_) Showing the Relative Stability and Local Spins Compatible with the Ni^II^Co^II^Ni^II^ Valence Form of Complex **2**

total spin (*S*_T_)	1/2	3/2	5/2	7/2	exp.
[*M*_s_(Ni_1_),*M*_s_(Co),*M*_s_(Ni_2_)]	[0,1/2,0]	[0,3/2,0]	[0,3/2,1]/[1,3/2,0]	[1,3/2,1]	
rel. energy (kcal/mol)	15.8	0	17.7	36.3	
SP(Co)	0.94	2.76	2.75	2.74	
SP(Ni_1_)	0.00	0.00	0.00(1.70)	1.71	
SP(Ni_2_)	0.00	0.00	1.70(0.00)	1.71	
R(Co–O)/Å	1.905	1.958	1.954	1.950	1.993
R(Ni–O)/Å	1.902	1.948	1.961	1.979	1.936
R(Ni–C)/Å	1.886	1.884	1.887(2.012)	2.016	1.861
			2.012(1.887)		
bite angles^a^ O–Co–O/°	78.7	78.9	78.6(82.9)	82.1	79.6
			82.9(78.6)		
bridging angles Ni–O–Co/°	101.4	100.7	100.8(97.8)	98.4	98.6
			97.8(100.8)		

a O–Co–O bite angles involving O atoms from the same [L^O,O^Ni] metalloligand.

**Figure 6:**
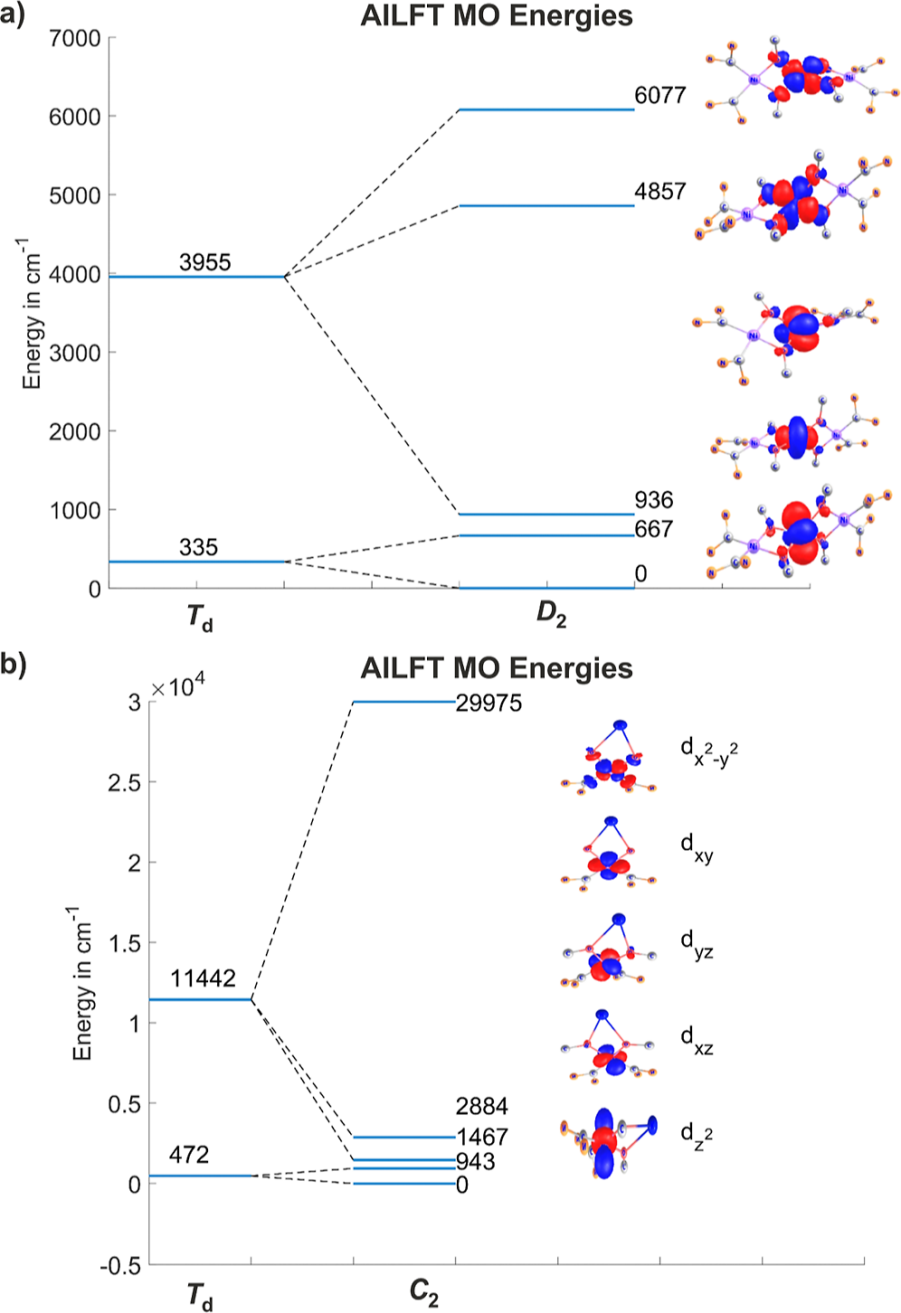
(a) Ab initio ligand field diagram of the Co^II^ 3d-MOs from CASSCF/NEVPT2 calculations of **2** without truncation. (b) Ab initio ligand field diagram of the Ni^II^ 3d-MOs from CASSCF/NEVPT2 calculations for [L^O,O^Ni] (**1**) without truncation.

**Figure 7:**
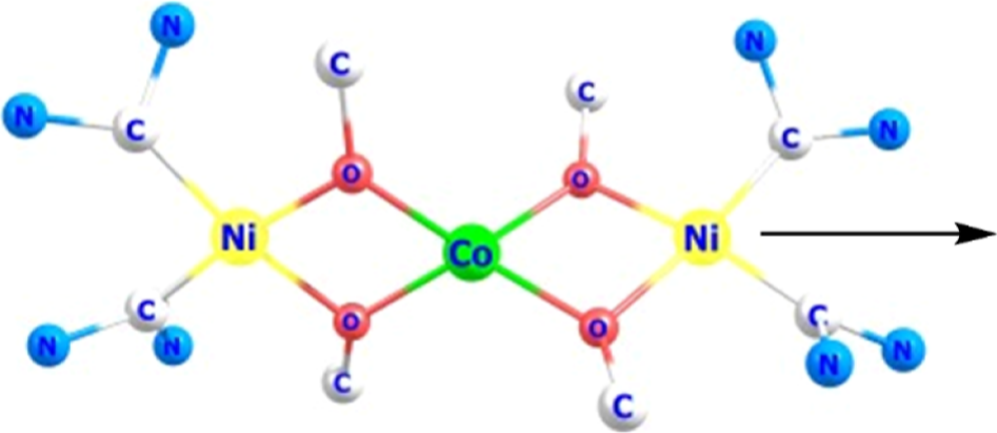
Direction of the easy magnetization axis in **2**.

Furthermore, complete active space self-consistent field (CASSCF)/ n-electron valence perturbation theory to second-order (NEVPT2) calculations on **2** were carried out considering the individual metal centers, Ni^II^ and Co^II^, as independent entities in the first approximation and accounting for their interactions in the second step. The CASSCF/NEVPT2 ab initio ligand field diagram (Figure 6a) shows the splitting of the metal’s e and t_2_ orbitals of the parent tetrahedral {CoO_4_} unit into d_*x*^2^–*y*^2^_, d_*z*^2^_, and d_*xy*_, d_*xz*_, d_*yz*_ subsets, respectively. Being doubly occupied in the ground state, the former two orbitals yield no contributions to the magnetic moments. However, the large splitting of the t_2_ orbital set induced by the [L^O,O^Ni] metalloligands and the resulting proximity of the d_*xy*_ next to d_*x*^2^–*y*^2^_ cause a large SOC mixing to induce ground-state magnetic moments and magnetic anisotropy along an easy magnetic direction parallel to the Ni_1_–Co–Ni_2_ vector (Figure 7). The availability of Ni^II^ complex **1** lacking the Co^II^ ion opens the possibility of extracting an analogous diagram for the [L^O,O^Ni] fragment and considering its effect on the ligand field of the central Co^II^ in **2** in the next step. The ordering of the 3d-MOs in the [L^O,O^Ni] chromophore (Figure 6b) is typical for closed-shell square planar complexes of Ni^II^ with four closely spaced low-energy d_*z*^2^_, d_*xz*_, d_*yz*_, and d_*xy*_ orbitals and the d_*x*^2^–*y*^2^_ orbital at about 30,000 cm^–1^ above.

The large ligand field splitting at the Ni^II^ results from the strong σ-antibonding interactions with both the alkoxide-O and the two NHC–C donors and is characteristic of diamagnetic square-planar Ni^II^(d^8^) systems, although some paramagnetic planar complexes of Ni^II^ have also been reported^57^ and are theoretically well understood.^58−61^

**Figure 8:**
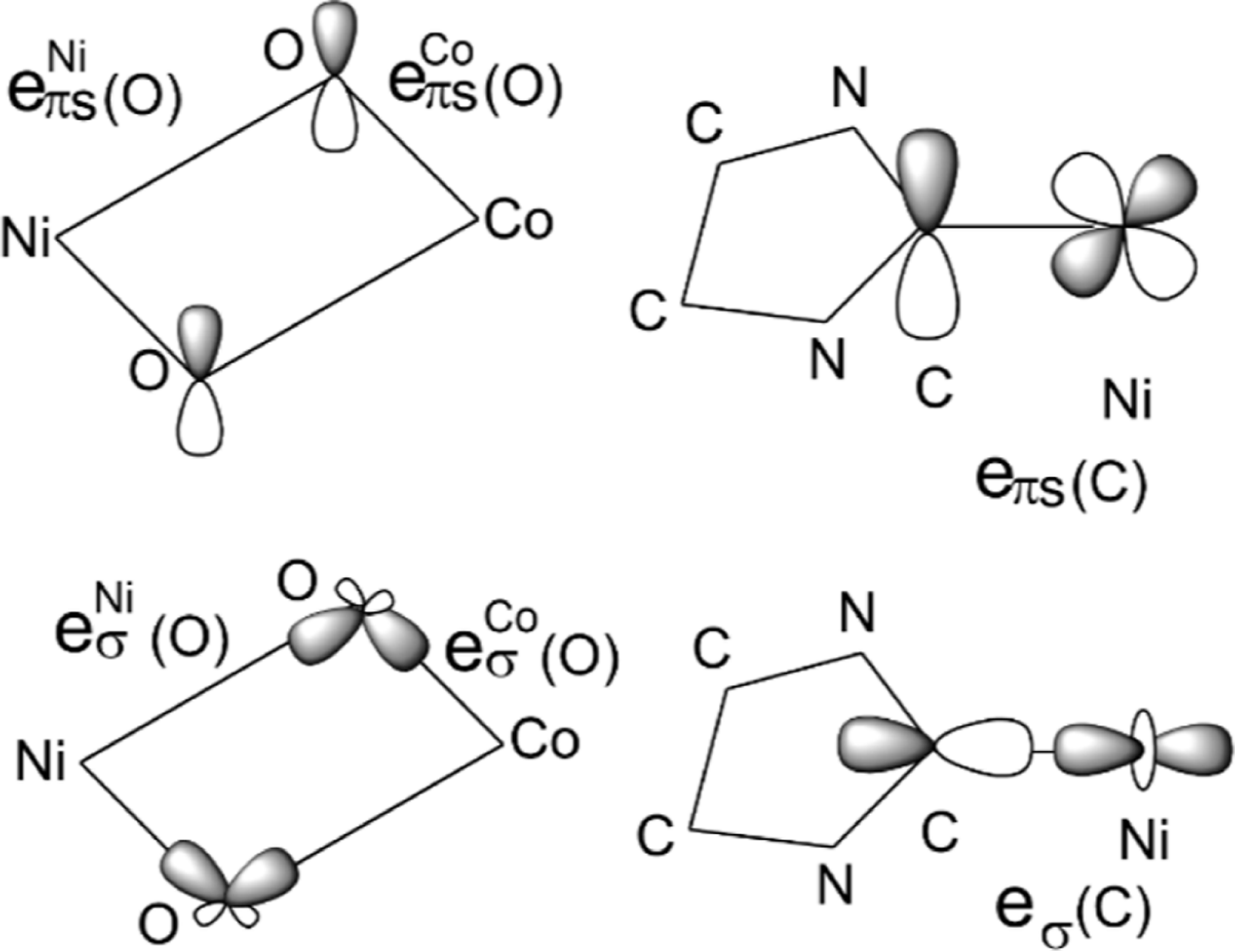
Angular overlap parametrization of the Ni^II^–O (alkoxide) and Ni–C (NHC) interactions (M = Co^II^, Ni^II^).

**Figure 9:**
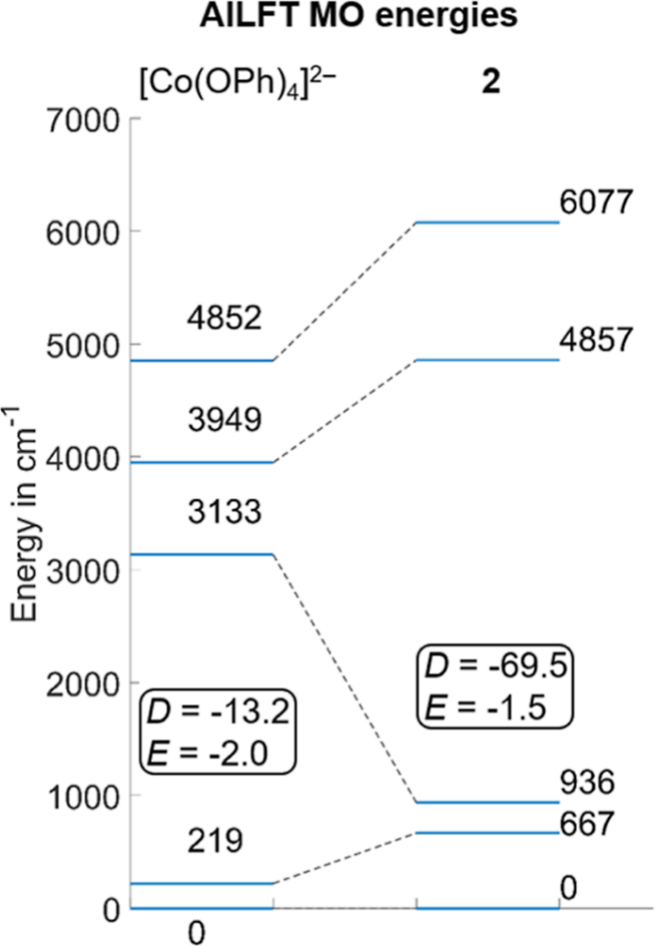
3d-MO ligand field splitting and ZFS parameters, *D* and *E* (all numerical entries are given in cm^–1^), of the Co^II^ ions in [Co(OPh)_4_]^2–^ (left) and **2** (right), illustrating the effect of the O-donor [L^O,O^Ni] metalloligand in **2**.

An analysis of the Ni–C and M–O bonds (M = Ni, Co) using the angular overlap model allows us to quantify metal–ligand interactions for both the Ni and Co ions in terms of two parameters, σ and out-of-plane π (denoted by πs) in the case of the Ni–C bonds, and three such parameters, viz., σ, πs, and πc, the latter accounting for in-plane π-bonding with the less anisotropic alkoxide-O, for the M–O bonds (Figure 8). Their values are listed in Table 2 (see the Supporting Information for details). The paramagnetic complex [Co(OPh)_4_]^2–^ (**A**, Figure 1) reported in an earlier study^22^ and magnetically characterized to have a small negative *D* value (−11.1 cm^–1^) is in good agreement with our CASSCF/NEVPT2 calculations reported here (*D* = −13.2 cm^–1^). Ab initio ligand field analyses characterize the PhO^–^ ligand as both σ- and π-donor toward Co^II^ (*e*_σ_ = 4570, *e*_πs_ = 1445, and *e*_πc_ = 1380 cm^–1^; see Table 2). The alkoxide-O donors adopt a bridging position between Ni^II^ and Co^II^ in **2**, which is accompanied by the lowering of *e*_σ_ to 3900 cm^–1^ as well as of *e*_πs_ and *e*_πc_ to small negative values, the latter reflecting a small yet non-negligible π-back bonding character (see Table 2). This significant change of the ligand field of the central Co^II^ upon going from [Co(OPh)_4_]^2–^ to **2** can be ascribed to the contrapolarizing effect of the Ni^II^ ions in the [L^O,O^Ni] metalloligands, which in their square-planar geometry form stronger bonds than the distorted tetrahedral Co^II^. The consequences are reflected in the large difference in the LF splitting diagram for the Co^II^ ion (Figure 9). As can be seen by the listed values of *D* (−69.5 cm^–1^) and *E* (−1.5 cm^–1^), drastic changes in the magnetic anisotropy are induced: an increase of the negative *D* by a factor of 5 is accompanied by a decrease of *E*. It is interesting to note that even with a small non-negligible *E*/*D* value that mixes the energy levels and is responsible for quenching the SMM behavior in 3d complexes, **2** surprisingly displays slow relaxation of the magnetization even in the absence of an external field, although the effect of QTM is obvious at lower temperatures (Figure 5).

**Table 2:** Angular Overlap Parameters Quantifying Ni^II^–Ligand Interactions in [L^O,O^Ni] and Co^II^–Ligand Interactions in [Co(OPh)_4_]^2–^^22^ and in the {Co^II^O_4_} Core of **2**

system	**1**	[Co(OPh)_4_]^2–^	**2**
M	Ni^II^	Co^II^	Co^II^
*e*σ(M–O)/cm^–1^	12546	4569	3931
*e*π_s_(M–O)/cm^–1^	5536	1445	–258
*e*π_c_(M–O)/cm^–1^	7144	1383	–678
*e*σ(M–C)/cm^–1^	12616		
*e*π_s_(M–C)/cm^–1^	1086		
standard dev./cm^–1^	1273	310	243

The computed many-particle spectra of the Ni^II^ and Co^II^ chromophores allow us to also rationalize the electronic absorption spectrum of **2**, showing d–d transitions at 17500 and 19000 cm^–1^, which are partly superimposed by a more intense band at 24,500 cm^–1^. Based on the CASSCF/NEVPT2 calculations (see Supporting Information), the former two can be assigned to transitions to the Co^II^^4^T_1_(^4^F) state that is split due to the lowered *D*_2_ symmetry and/or SOC. The band at 24,500 cm^–1^ can be interpreted in terms of a more intense d–d transition within the [L^O,O^Ni] chromophore.

## Discussion, Conclusions, and Outlook

The complex [L^O,O^Ni] with a Ni^II^ (*S* = 0) ion in square planar geometry was shown to serve as an {O,O′}-metalloligand toward other metal ions, and the replacement of K^+^ in the bimetallic complex [(L^O,O^Ni)K(MeCN)(OTf)] (**1**) by divalent Co^II^ led to the trinuclear complex [(L^O,O^Ni)Co(L^O,O^Ni)] (**2**) featuring a central Co^II^ ion in a distorted (elongated) tetrahedral {O_4_} donor environment. The two carbene-C and alkoxide-O donors induce a rather strong ligand field on the Ni^II^ ion in the [L^O,O^Ni] subunits, which translates into a corresponding weakening of the ligand field at the central Co^II^ site. Indeed, transition metal M–O bonds were found to be very variable, depending strongly on the electronic and geometric structure around the O-donor ligand, specifically on the presence of contrapolarizing cations besides M at the ligating O-donor atoms.^62^ This has been shown by analyzing optical and EPR spectroscopic data for oxidic solid networks composed of one or more transition metal ions and oxido (O^2–^) ligands. The information gained by detailed ligand field analysis of such spectra revealed that, for example, compounds with {MO_6_} units [M = Ni^II^(d^8^), Co^II^(d^7^), and Cu^II^(d^9^)] with bridging oxido ligands differ considerably from high-valent Fe^IV^, Mn^V^, and Mn^VI^ complexes with “terminal” O-donors that induce stronger ligand fields. In the former case, lower ligand fields result, and for Co^II^, invariably high-spin (*S* = 3/2) states with {CoO_6_} or {CoO_4_} entities are stabilized.^62^ In the latter case, highly covalent M–O bonds with almost equal sharing of the valence electrons between O^2–^ and the respective transition metal ion result. The complex **2** reported here can be assigned to the first category, where the contrapolarizing electronic effect of the second-sphere Ni^II^ ions induces a small d_*x*^2^–*y*^2^_–d_*xy*_ energy gap and concomitant mixing of these two orbitals via SOC in the ground magnetic *S* = 3/2 state. This leads to the observed magnetic anisotropy and the slow magnetic relaxation. The mentioned electronic effects are favorably supported by optimal geometrical constraints reflected by the acute O–Co–O bite angle on the central Co^II^ site enforced by the bidentate [L^O,O^Ni] metalloligands as well as the close to 90° dihedral angle between the two O–Co–O chelate planes, which results in a close to *D*_2*d*_ axial geometry. All these factors contribute to maximizing the magnetic anisotropy of the new system, which is 5-fold enhanced over the magnetic anisotropy of [Co(OPh)_4_]K(PPh_4_) (**A**), leading to slow spin relaxation in zero field with an effective energy barrier of up to 86.9 cm^–1^. It is a promising perspective to enhance the second coordination sphere effect by using {O,O′}-metalloligands with metal ions other than Ni^II^, in particular more strongly contrapolarizing high-valent metal ions that generate highly ionic O-donor atoms for the central Co^II^. Hence, these combined effects may open a new direction for designing SIMs even with hard oxygen donors based on rigid metalloligands, which complement most of the prominent Co and Fe-based SIMs reported to date that feature soft donor ligands or N/C coordinated metal ions in a low coordination geometry (see Tables S8 and S9).

Results from this study can also be placed in a more general context related to the reactivity of O-donor-containing transition metal complexes that act as catalysts in charge transfer-type reactions. Specifically, second-coordination sphere effects on O-donor ligation, e.g., by coordination of additional metal ions, may induce ambivalence in the O-donor properties at the active site.^63^ When combined with vibronic coupling, this aspect may become important in both homogeneous and heterogeneous catalysis and may be analyzed and exploited both spectroscopically and by theoretical means.

## Experimental Section

### Materials

All air- and moisture-sensitive compounds were handled under an anaerobic and anhydrous atmosphere of dry argon, using standard Schlenk techniques, or in an Ar-filled MBraun glovebox. Imidazole-diphenylethanol^52^ and 1,2-bis(trifluoromethylsulfonyloxyl)ethane^53^ were prepared following literature procedures. Unless otherwise stated, all chemicals used were purchased from commercial sources and used without further purification. The solvents were dried following standard procedures, stored under molecular sieves (3 Å), and degassed with Ar.

### Instruments for Spectroscopic and Analytical Characterization

^1^H and ^13^C NMR spectra were recorded on a Bruker AVANCE 300 or 400 MHz spectrometer at 298 K. ^13^C NMR spectra were recorded in the proton-decoupled mode. ESI–MS measurements were performed on a Thermo Finnigan Trace LCQ spectrometer or a Bruker Apex IV (FTICR-MS). The MALDI mass spectrometric analysis was carried out on a MALDI-TOF Autoflex Speed mass spectrometer from Bruker Daltonik. UV/vis spectra were recorded on a Varian Cary 5000 or Varian Cary 60 machine, using quartz cells (1 cm) in the solvent indicated. Diffuse reflectance UV–vis–NIR spectra of solid samples were recorded with a Varian Cary 5000 spectrophotometer by diluting the complex in dry KBr. Solid-state IR spectra were recorded with a Cary 630 FTIR spectrometer with Dial Path Technology and analyzed by FTIR Microlab software. Elemental analyses were performed by the analytical laboratory of the Institute of Inorganic Chemistry at the University of Göttingen using an Elementar Vario EL III instrument.

### Single-Crystal Structure Determinations

Crystal data and details of the data collection are listed in Table S1. X-ray data were collected on an STOE IPDS II or a BRUKER D8-QUEST diffractometer (monochromated Mo–Kα radiation, λ = 0.71073 Å) by the use of ω or ω and ϕ scans at low temperature. The structures were solved with SHELXT^64^ and refined on *F*^2^ using all reflections with SHELXL.^65^ Non-hydrogen atoms were refined anisotropically. Hydrogen atoms were placed in calculated positions and assigned to an isotropic displacement parameter of 1.5 or 1.2 *U*_eq_(C). In the case of **2**, a CF_3_SO_3_^–^ anion was found to be disordered at about two positions [occupancy factors 0.653(8)/0.347(8)]. SAME and RIGU restraints and EADP constraints were used for the refinement of the disordered parts. The unit cell of **2**, furthermore, contains highly disordered acetonitrile solvent molecules, for which no satisfactory model of disorder could be found. The solvent contribution to the structure factors was calculated with PLATON SQUEEZE,^66^ and the resulting .fab file was processed with SHELXL using the ABIN instruction. The empirical formula and derived values are in accordance with the calculated cell content. Face-indexed absorption corrections were performed numerically with the program X-RED,^67^ or in the case of **2**, by the multiscan method with SADABS.^68^ CCDC 2311463 (**1**) and 2311464 (**2**) contain the supplementary crystallographic data for this paper. This data can be obtained free of charge from the Cambridge Crystallographic Data Centre via www.ccdc.cam.ac.uk/structures.

### Magnetic Studies

Magnetic measurements were carried out with a Quantum-Design MPMS3 SQUID magnetometer equipped with a 7.0 T magnet. Dc magnetic susceptibility measurements were performed under an applied dc field with powdered polycrystalline samples in the range from 210 to 2 K. The powdered samples were packed in a polycarbonate capsule and covered with low-viscosity perfluoropolyether-based inert oil, Fomblin Y45, in a nonmagnetic sample holder. Each raw data for the measured magnetic moment was corrected for the diamagnetic contribution of the capsules, including the inert oil, if used according to *M*_dia_(capsule) = χ_g_·*m*·*H*, with an experimentally obtained gram susceptibility of the capsules, including the inert oil. The molar susceptibility data were corrected for the diamagnetic contribution. Experimental data were modeled with the julX_2s program.^55^ Ac susceptibility measurements were carried out in an oscillating ac field of 3.0 Oe and frequencies ranging from 0.1 to 1000 Hz.

### Safety Statement

All of the new chemicals synthesized in this work were handled in a well-ventilated fume hood wearing gloves and safety glasses or inside a glovebox under an inert atmosphere. The new compounds are stable at room temperature under an inert atmosphere, and no special risks or hazards were encountered during the investigation.

### Synthesis Protocols

#### [H_4_L^O,O^](OTf)_2_

Imidazole-diphenylethanol (7.41 g, 0.028 mol, 2 equiv) was dissolved in MeCN (200 mL), and the solution was heated at 80 °C, followed by the addition of 1,2-bis(trifluoromethylsulfonyloxyl)ethane (4.75 g, 1.79 mL, 0.014 mol, 1 equiv). The reaction was stirred at reflux overnight and then cooled to room temperature. The solvent was removed under reduced pressure. The product was obtained as a white powder after crystallization from the THF/Et_2_O mixture. Yield: 9.075 g, 78%. ^1^H NMR (300 MHz, DMSO-*d*_6_) δ (ppm): 4.56 (s, 2H), 5.07 (s, 2H), 6.62 (s, 1H), 7.17 (s, 1H), 7.22 (s, 1H), 7.25–7.45 (m, 10H), 8.78 (s, 1H). ^13^C{^1^H} NMR (75 MHz, DMSO-*d*_6_) δ (ppm): 48.3, 58.0, 76.3, 121.2, 124.3, 126.1, 127.7, 128.5, 137.3, 143.8. UV/vis (MeCN) λ [nm] (ε [M^–1^ cm^–1^]): 260 (1.13 × 10^3^). MALDI–MS *m*/*z*: 705.4 [H_4_L(OTf)]^+^. IR ṽ [cm^–1^]: 695 (s), 749 (s), 779 (m), 846 (m), 870 (m), 1027 (s), 1066 (m), 1154 (s), 1220 (s), 1250 (s), 1271 (m), 1450 (m), 1560 (m), 3084 (w), 3105 (w), 3138 (w),3406 (br).

#### [(L^O,O^Ni)K(MeCN)(OTf)] (**1**)

[H_4_L^O,O^](OTf)_2_ (0.5 g, 0.585 mmol, 1 equiv) was dissolved in 20 mL of THF, followed by the addition of solid NiBr_2_·DME (0.180 g, 0.585 mmol, 1 equiv). A solution of ^*t*^BuOK (0.262 g, 2.34 mmol, 4 equiv) in 10 mL of THF was then added dropwise. The reaction mixture was stirred for 24 h at room temperature, and the solvent was removed under vacuum. The crude solid was washed with CH_2_Cl_2_, and the solution was filtered. The solvent was removed, and light-yellow single crystals were obtained by the diffusion of Et_2_O in a solution of MeCN. Yield: 0.20 g (40%). ^1^H NMR (300 MHz, CD_3_CN) δ (ppm): 4.33 (s, 2H), 4.78 (s, 2H), 6.66 (s, 1H), 6.78 (s, 1H), 7.10–7.55 (m, 10H). ^13^C NMR (75 MHz, CD_3_CN) δ (ppm): 48.8, 63.0, 76.8, 121.9, 123.2, 126.8, 127.9, 128.5, 149.9, 162.2. UV/vis (MeCN) λ [nm] (ε [M^–1^ cm^–1^]): 320 (6.3 × 10^3^). Anal. Calcd for C_39_H_35_F_3_KN_5_NiO_5_S: C, 55.7; H, 4.2; N, 8.3. Found: C, 55.3; H, 4.2; N, 8.3. ESI–MS(+) *m*/*z*: 611.3 [M – K(MeCN)(OTf) + H]^+^. IR ṽ [cm^–1^]: 698 (s), 756 (m), 1028 (s), 1161 (s), 1248 (s), 1444 (w), 1653 (w).

#### [(L^O,O^Ni)_2_Co](OTf)_2_ (**2**)

Solid **1** (30 mg, 0.036 mmol, 1 equiv) was added to the solution of anhydrous Co(OTf)_2_ (6.4 mg, 0.018 mmol, 0.5 equiv) in 6 mL of MeCN. The solution was stirred for 48 h at room temperature. The solvent was then removed in vacuo, and the crude was dissolved in the minimum quantity of acetone. The resulting solution was filtered. Crystallization by the slow diffusion of Et_2_O at −27 °C led to the isolation of reddish-green single crystals. Yield: 40 mg (70%). UV/vis (MeCN) λ (nm) (ε [M^–1^ cm^–1^]): 300 (1.9 × 10^4^), 409 (7.4 × 10^2^), 567 (3.3 × 10^2^). Anal. Calcd for C_74_H_64_CoF_6_N_8_Ni_2_O_10_S_2_: C, 56.26; H, 4.08; N, 7.09. Found: C, 56.18, 4.06; N, 7.09. ESI–MS(+) *m*/*z*: 640.6 [M – 2(OTf)]^2+^. IR ṽ [cm^–1^]: 669 (m), 676 (m), 690 (s), 697 (s), 727 (m), 751 (m), 783 (m), 898 (m), 960 (m), 1029 (s), 1159 (m), 1154 (m), 1222 (m), 1252 (s), 1278 (m), 1446 (w), 3023 (w), 3055 (w), 3124 (w).

### Theoretical Calculations

Calculations were carried out with the ORCA package^69−71^ using coordinates obtained from the X-ray crystallographic data. The spectrum of the Hamiltonian was computed using the state-averaged CASSCF (SA-CASSCF)^72−74^ with NEVPT-2.^75−77^ Scalar relativistic effects were taken into account via the Douglas–Kroll–Hess (DKH) method of second order.^78−81^ Ahlrichs-polarized def2-TZVPP basis sets^82−84^ optimized for DKH method^85^ were used for all calculations. Resolution of identity along with the corresponding auxiliary basis set^86^ was used to speed up calculations. An active space of seven electrons on five active 3d molecular orbitals CAS(7,5) was employed. The *S* = 3/2 (*S* = 1/2) states of the free ion (^4^F, ^4^P and ^2^P, ^2^D, ^2^F, ^2^G, and ^2^H, respectively) give rise to ten quartets (40 doublets) of the complex. The absorption spectra of the complex are plotted with the orca_mapspc utility. The ZFS tensor ***D*** of the spin Hamiltonian^87−89^ was extracted using effective Hamiltonian theory from a mapping onto the full SOC of many-particle energy eigenvalues and wave functions (for details, see Supporting Information).

## Supporting Information Available

^1^H and ^13^C NMR, FTIR, UV–vis–NIR, and mass spectra for the new compounds reported in this work; additional magnetic data and crystallographic details; and details/results of the theoretical calculations including input files and all xyz coordinates (PDF)

## References

[ref1] Gatteschi D.; Sessoli R.; Villain J. Molecular Nanomagnets; Oxford University Press: New York, 2011.

[ref2] Shiddiq M.; Komijani D.; Duan Y.; Gaita-Ariño A.; Coronado E.; Hill S. Enhancing coherence in molecular spin qubits via atomic clock transitions. Nature 2016, 531 (7594), 348–351. 10.1038/nature16984. 26983539

[ref3] Bogani L.; Wernsdorfer W. Molecular spintronics using single-molecule magnets. Nat. Mater. 2008, 7 (3), 179–186. 10.1038/nmat2133. 18297126

[ref4] Urdampilleta M.; Klyatskaya S.; Cleuziou J. P.; Ruben M.; Wernsdorfer W. Supramolecular spin valves. Nat. Mater. 2011, 10 (7), 502–506. 10.1038/nmat3050. 21685902

[ref5] Aravena D.; Ruiz E. Spin dynamics in single-molecule magnets and molecular qubits. Dalton Trans. 2020, 49 (29), 9916–9928. 10.1039/D0DT01414A. 32589181

[ref6] Holynska M. Single-Molecule Magnets: Molecular Architectures and Building Blocks for Spintronics; Wiley, 2019.

[ref7] Sarkar A.; Dey S.; Rajaraman G. Role of Coordination Number and Geometry in Controlling the Magnetic Anisotropy in Fe(II), Co(II), and Ni(II) Single-Ion Magnets. Chem.—Eur. J. 2020, 26 (62), 14036–14058. 10.1002/chem.202003211. 32729641

[ref8] Murrie M. Cobalt(II) single-molecule magnets. Chem. Soc. Rev. 2010, 39 (6), 1986–1995. 10.1039/b913279c. 20422103

[ref9] Gómez-Coca S.; Aravena D.; Morales R.; Ruiz E. Large magnetic anisotropy in mononuclear metal complexes. Coord. Chem. Rev. 2015, 289–290, 379–392. 10.1016/j.ccr.2015.01.021.

[ref10] Frost J. M.; Harriman K. L. M.; Murugesu M. The rise of 3-d single-ion magnets in molecular magnetism: towards materials from molecules?. Chem. Sci. 2016, 7 (4), 2470–2491. 10.1039/C5SC03224E. 28660017 PMC5477015

[ref11] Cirera J.; Ruiz E.; Alvarez S.; Neese F.; Kortus J. How to Build Molecules with Large Magnetic Anisotropy. Chem.—Eur. J. 2009, 15 (16), 4078–4087. 10.1002/chem.200801608. 19248077

[ref12] Neese F.; Pantazis D. A. What is not required to make a single molecule magnet. Faraday Discuss. 2011, 148 (0), 229–238. 10.1039/C005256F. 21322486

[ref13] Waldmann O. A Criterion for the Anisotropy Barrier in Single-Molecule Magnets. Inorg. Chem. 2007, 46 (24), 10035–10037. 10.1021/ic701365t. 17979271

[ref14] Zadrozny J. M.; Xiao D. J.; Atanasov M.; Long G. J.; Grandjean F.; Neese F.; Long J. R. Magnetic blocking in a linear iron(I) complex. Nat. Chem. 2013, 5 (7), 577–581. 10.1038/nchem.1630. 23787747

[ref15] Bunting P. C.; Atanasov M.; Damgaard-Møller E.; Perfetti M.; Crassee I.; Orlita M.; Overgaard J.; van Slageren J.; Neese F.; Long J. R. A linear cobalt(II) complex with maximal orbital angular momentum from a non-Aufbau ground state. Science 2018, 362 (6421), eaat7319 10.1126/science.aat7319. 30442763

[ref16] Yao X.-N.; Du J.-Z.; Zhang Y.-Q.; Leng X.-B.; Yang M.-W.; Jiang S.-D.; Wang Z.-X.; Ouyang Z.-W.; Deng L.; Wang B.-W.; Gao S. Two-Coordinate Co(II) Imido Complexes as Outstanding Single-Molecule Magnets. J. Am. Chem. Soc. 2017, 139 (1), 373–380. 10.1021/jacs.6b11043. 27936686

[ref17] Atanasov M.; Zadrozny J. M.; Long J. R.; Neese F. A theoretical analysis of chemical bonding, vibronic coupling, and magnetic anisotropy in linear iron(ii) complexes with single-molecule magnet behavior. Chem. Sci. 2013, 4 (1), 139–156. 10.1039/C2SC21394J.

[ref18] Tripathi S.; Dey A.; Shanmugam M.; Narayanan R. S.; Chandrasekhar V. Cobalt(II) Complexes as Single-Ion Magnets. In Organometallic Magnets; Chandrasekhar V., Pointillart F., Eds.; Springer International Publishing: Cham, 2019; pp 35–75.

[ref19] Carl E.; Demeshko S.; Meyer F.; Stalke D. Triimidosulfonates as Acute Bite-Angle Chelates: Slow Relaxation of the Magnetization in Zero Field and Hysteresis Loop of a CoII Complex. Chem.—Eur. J. 2015, 21 (28), 10109–10115. 10.1002/chem.201406083. 26043416

[ref20] Fataftah M. S.; Coste S. C.; Vlaisavljevich B.; Zadrozny J. M.; Freedman D. E. Transformation of the coordination complex [Co(C_3_S_5_)_2_]^2-^ from a molecular magnet to a potential qubit. Chem. Sci. 2016, 7 (9), 6160–6166. 10.1039/C6SC02170K. 30034755 PMC6024178

[ref21] Vaidya S.; Tewary S.; Singh S. K.; Langley S. K.; Murray K. S.; Lan Y.; Wernsdorfer W.; Rajaraman G.; Shanmugam M. What Controls the Sign and Magnitude of Magnetic Anisotropy in Tetrahedral Cobalt(II) Single-Ion Magnets?. Inorg. Chem. 2016, 55 (19), 9564–9578. 10.1021/acs.inorgchem.6b01073. 27652694

[ref22] Zadrozny J. M.; Telser J.; Long J. R. Slow magnetic relaxation in the tetrahedral cobalt(II) complexes [Co(EPh)_4_]^2-^ (E = O, S, Se). Polyhedron 2013, 64, 209–217. 10.1016/j.poly.2013.04.008.

[ref23] Bamberger H.; Albold U.; Dubnická Midlíková J.; Su C.-Y.; Deibel N.; Hunger D.; Hallmen P. P.; Neugebauer P.; Beerhues J.; Demeshko S.; Meyer F.; Sarkar B.; van Slageren J. Iron(II), Cobalt(II), and Nickel(II) Complexes of Bis(sulfonamido)benzenes: Redox Properties, Large Zero-Field Splittings, and Single-Ion Magnets. Inorg. Chem. 2021, 60 (5), 2953–2963. 10.1021/acs.inorgchem.0c02949. 33591172

[ref24] Rechkemmer Y.; Breitgoff F. D.; van der Meer M.; Atanasov M.; Hakl M.; Orlita M.; Neugebauer P.; Neese F.; Sarkar B.; van Slageren J. A four-coordinate cobalt(II) single-ion magnet with coercivity and a very high energy barrier. Nat. Commun. 2016, 7 (1), 10467 10.1038/ncomms10467. 26883902 PMC4757785

[ref25] Legendre C. M.; Damgaard-Møller E.; Overgaard J.; Stalke D. The Quest for Optimal 3 d Orbital Splitting in Tetrahedral Cobalt Single-Molecule Magnets Featuring Colossal Anisotropy and Hysteresis. Eur. J. Inorg. Chem. 2021, 2021 (30), 3108–3114. 10.1002/ejic.202100465.

[ref26] Gupta S. K.; Nielsen H. H.; Thiel A. M.; Klahn E. A.; Feng E.; Cao H. B.; Hansen T. C.; Lelièvre-Berna E.; Gukasov A.; Kibalin I.; Dechert S.; Demeshko S.; Overgaard J.; Meyer F. Multi-Technique Experimental Benchmarking of the Local Magnetic Anisotropy of a Cobalt(II) Single-Ion Magnet. JACS Au 2023, 3 (2), 429–440. 10.1021/jacsau.2c00575. 36873706 PMC9975825

[ref27] Cui H.-H.; Lu F.; Chen X.-T.; Zhang Y.-Q.; Tong W.; Xue Z.-L. Zero-Field Slow Magnetic Relaxation and Hysteresis Loop in Four-Coordinate Co^II^ Single-Ion Magnets with Strong Easy-Axis Anisotropy. Inorg. Chem. 2019, 58 (19), 12555–12564. 10.1021/acs.inorgchem.9b01175. 31553166

[ref28] Boča R.; Miklovič J.; Titiš J. Simple Mononuclear Cobalt(II) Complex: A Single-Molecule Magnet Showing Two Slow Relaxation Processes. Inorg. Chem. 2014, 53 (5), 2367–2369. 10.1021/ic5000638. 24524226

[ref29] Wu T.; Zhai Y.-Q.; Deng Y.-F.; Chen W.-P.; Zhang T.; Zheng Y.-Z. Correlating magnetic anisotropy with the subtle coordination geometry variation of a series of cobalt(II)-sulfonamide complexes. Dalton Trans. 2019, 48 (41), 15419–15426. 10.1039/C9DT01296F. 31065655

[ref30] Gupta S. K.; Rao S. V.; Demeshko S.; Dechert S.; Bill E.; Atanasov M.; Neese F.; Meyer F. Air-stable four-coordinate cobalt(II) single-ion magnets: experimental and ab initio ligand field analyses of correlations between dihedral angles and magnetic anisotropy. Chem. Sci. 2023, 14 (23), 6355–6374. 10.1039/D3SC00813D. 37325133 PMC10266464

[ref31] Maganas D.; Sottini S.; Kyritsis P.; Groenen E. J. J.; Neese F. Theoretical Analysis of the Spin Hamiltonian Parameters in Co(II)S_4_ Complexes, Using Density Functional Theory and Correlated ab initio Methods. Inorg. Chem. 2011, 50 (18), 8741–8754. 10.1021/ic200299y. 21848258

[ref32] Saber M. R.; Dunbar K. R. Ligands effects on the magnetic anisotropy of tetrahedral cobalt complexes. Chem. Commun. 2014, 50 (82), 12266–12269. 10.1039/C4CC05724D. 25183324

[ref33] Vaidya S.; Upadhyay A.; Singh S. K.; Gupta T.; Tewary S.; Langley S. K.; Walsh J. P. S.; Murray K. S.; Rajaraman G.; Shanmugam M. A synthetic strategy for switching the single ion anisotropy in tetrahedral Co(II) complexes. Chem. Commun. 2015, 51 (18), 3739–3742. 10.1039/C4CC08305A. 25370832

[ref34] Vaidya S.; Singh S. K.; Shukla P.; Ansari K.; Rajaraman G.; Shanmugam M. Role of Halide Ions in the Nature of the Magnetic Anisotropy in Tetrahedral Co^II^ Complexes. Chem.—Eur. J. 2017, 23 (40), 9546–9559. 10.1002/chem.201606031. 28512770

[ref35] Sarkar A.; Tewary S.; Sinkar S.; Rajaraman G. Magnetic Anisotropy in Co^II^X_4_ (X = O, S, Se) Single-Ion Magnets: Role of Structural Distortions versus Heavy Atom Effect. Chem.—Asian J. 2019, 14 (24), 4696–4704. 10.1002/asia.201901140. 31489772

[ref36] Suturina E. A.; Maganas D.; Bill E.; Atanasov M.; Neese F. Magneto-Structural Correlations in a Series of Pseudotetrahedral [Co^II^(XR)_4_]^2--^ Single Molecule Magnets: An ab Initio Ligand Field Study. Inorg. Chem. 2015, 54 (20), 9948–9961. 10.1021/acs.inorgchem.5b01706. 26443918

[ref37] Suturina E. A.; Nehrkorn J.; Zadrozny J. M.; Liu J.; Atanasov M.; Weyhermüller T.; Maganas D.; Hill S.; Schnegg A.; Bill E.; Long J. R.; Neese F. Magneto-Structural Correlations in Pseudotetrahedral Forms of the [Co(SPh)_4_]^2–^ Complex Probed by Magnetometry, MCD Spectroscopy, Advanced EPR Techniques, and ab Initio Electronic Structure Calculations. Inorg. Chem. 2017, 56 (5), 3102–3118. 10.1021/acs.inorgchem.7b00097. 28225611

[ref38] Tripathi S.; Vaidya S.; Ansari K. U.; Ahmed N.; Rivière E.; Spillecke L.; Koo C.; Klingeler R.; Mallah T.; Rajaraman G.; Shanmugam M. Influence of a Counteranion on the Zero-Field Splitting of Tetrahedral Cobalt(II) Thiourea Complexes. Inorg. Chem. 2019, 58 (14), 9085–9100. 10.1021/acs.inorgchem.9b00632. 31246445

[ref39] Ziegenbalg S.; Hornig D.; Görls H.; Plass W. Cobalt(II)-Based Single-Ion Magnets with Distorted Pseudotetrahedral [N_2_O_2_] Coordination: Experimental and Theoretical Investigations. Inorg. Chem. 2016, 55 (8), 4047–4058. 10.1021/acs.inorgchem.6b00373. 27045421

[ref40] Wang M.; Xu H. J.; Sun T. M.; Cui H. H.; Zhang Y.-Q.; Chen L.; Tang Y. F. Optimal N-Co-N bite angle for enhancing the magnetic anisotropy of zero-field Co(II) single-ion magnets in tetrahedral [N_4_] coordination environment. J. Solid State Chem. 2021, 299, 122209 10.1016/j.jssc.2021.122209.

[ref41] Atanasov M.; Aravena D.; Suturina E.; Bill E.; Maganas D.; Neese F. First principles approach to the electronic structure, magnetic anisotropy and spin relaxation in mononuclear 3d-transition metal single molecule magnets. Coord. Chem. Rev. 2015, 289–290, 177–214. 10.1016/j.ccr.2014.10.015.

[ref42] Albold U.; Bamberger H.; Hallmen P. P.; van Slageren J.; Sarkar B. Strong Exchange Couplings Drastically Slow Down Magnetization Relaxation in an Air-Stable Cobalt(II)-Radical Single-Molecule Magnet (SMM). Angew. Chem., Int. Ed. 2019, 58 (29), 9802–9806. 10.1002/anie.201904645. PMC6771987 31050153

[ref43] Atanasov M.; Neese F. Computational Studies on Vibronic Coupling in Single Molecule Magnets: Impact on the Mechanism of Magnetic Relaxation. Journal of Physics: Conference Series; IOP Publishing, 2018; p 012006.

[ref44] Zadrozny J. M.; Long J. R. Slow Magnetic Relaxation at Zero Field in the Tetrahedral Complex [Co(SPh)_4_]^2–^. J. Am. Chem. Soc. 2011, 133 (51), 20732–20734. 10.1021/ja2100142. 22142241

[ref45] Kumar Sahu P.; Kharel R.; Shome S.; Goswami S.; Konar S. Understanding the unceasing evolution of Co(II) based single-ion magnets. Coord. Chem. Rev. 2023, 475, 214871 10.1016/j.ccr.2022.214871.

[ref46] Zabala-Lekuona A.; Landart-Gereka A.; Quesada-Moreno M. M.; Mota A. J.; Díaz-Ortega I. F.; Nojiri H.; Krzystek J.; Seco J. M.; Colacio E. Zero-Field SMM Behavior Triggered by Magnetic Exchange Interactions and a Collinear Arrangement of Local Anisotropy Axes in a Linear Co_3_^II^ Complex. Inorg. Chem. 2023, 62 (49), 20030–20041. 10.1021/acs.inorgchem.3c02817. 37991724 PMC10716897

[ref47] Meyer S.; Orben C. M.; Demeshko S.; Dechert S.; Meyer F. Synthesis and Characterization of Di- and Tetracarbene Iron(II) Complexes with Chelating N-Heterocyclic Carbene Ligands and Their Application in Aryl Grignard-Alkyl Halide Cross-Coupling. Organometallics 2011, 30 (24), 6692–6702. 10.1021/om200870w.

[ref48] Liu Y.; Resch S. G.; Klawitter I.; Cutsail G. E.; Demeshko S.; Dechert S.; Kühn F. E.; DeBeer S.; Meyer F. An Adaptable N-Heterocyclic Carbene Macrocycle Hosting Copper in Three Oxidation States. Angew. Chem., Int. Ed. 2020, 59 (14), 5696–5705. 10.1002/anie.201912745. PMC7154638 31769151

[ref49] Klawitter I.; Meyer S.; Demeshko S.; Meyer F. Nickel(II) and Iron(II) Complexes with Tetradentate NHC/Amide Hybrid Ligands. Z. Naturforsch. B 2013, 68 (5–6), 458–466. 10.5560/znb.2013-3091.

[ref50] Upadhyay A.; Singh S. K.; Das C.; Mondol R.; Langley S. K.; Murray K. S.; Rajaraman G.; Shanmugam M. Enhancing the effective energy barrier of a Dy(III) SMM using a bridged diamagnetic Zn(II) ion. Chem. Commun. 2014, 50 (64), 8838–8841. 10.1039/C4CC02094D. 24824019

[ref51] Upadhyay A.; Das C.; Vaidya S.; Singh S. K.; Gupta T.; Mondol R.; Langley S. K.; Murray K. S.; Rajaraman G.; Shanmugam M. Role of the Diamagnetic Zinc(II) Ion in Determining the Electronic Structure of Lanthanide Single-Ion Magnets. Chem.—Eur. J. 2017, 23 (20), 4903–4916. 10.1002/chem.201700399. 28177539

[ref52] Shimizu S.; Ogata M. Fluoride- or alkoxide-induced reaction of 1-[(trimethylsilyl)methyl]azoles with carbonyl compounds. J. Org. Chem. 1986, 51 (20), 3897–3900. 10.1021/jo00370a028.

[ref53] Yue Z.; Dunya H.; Aryal S.; Segre C. U.; Mandal B. Synthesis and electrochemical properties of partially fluorinated ether solvents for lithiumsulfur battery electrolytes. J. Power Sources 2018, 401, 271–277. 10.1016/j.jpowsour.2018.08.097.

[ref54] Yang L.; Powell D. R.; Houser R. P. Structural variation in copper(I) complexes with pyridylmethylamide ligands: structural analysis with a new four-coordinate geometry index, τ_4_. Dalton Trans. 2007, (9), 955–964. 10.1039/B617136B. 17308676

[ref55] Bill E. julX_2S Program for Simulation of Molecular Magnetic Data; Max-Planck Institute for Chemical Energy Conversion: Mülheim/Ruhr, Germany, 2014.

[ref56] Reta D.; Chilton N. F. Uncertainty estimates for magnetic relaxation times and magnetic relaxation parameters. Phys. Chem. Chem. Phys. 2019, 21 (42), 23567–23575. 10.1039/C9CP04301B. 31620733

[ref57] Frömmel T.; Peters W.; Wunderlich H.; Kuchen W. Bis(P,P-di-tert-butylphosphinic-N-isopropylamidato-N,O)nickel(II), a Paramagnetic Planar Complex. Angew. Chem., Int. Ed. 1992, 31 (5), 612–613. 10.1002/anie.199206121.

[ref58] Mink H. J.; Schmidtke H.-H. Optical properties of Ni[t-Bu_2_P(O)NR]_2_: A paramagnetic d^8^ complex with planar structure. Chem. Phys. Lett. 1994, 231 (2–3), 235–240. 10.1016/0009-2614(94)01248-2.

[ref59] Bridgeman A. J.; Gerloch M. Comment on optical properties of Ni[t-Bu_2_P(O)NR]_2_. A paramagnetic d^8^ complex with planar structure. Chem. Phys. Lett. 1995, 247 (3), 304–309. 10.1016/0009-2614(95)00679-8.

[ref60] Mink H.-J.; Schmidtke H.-H. Reply to comment on optical properties of Ni[t-Bu_2_P(O)NR]_2_. A paramagnetic d^8^ complex with planar structure. Chem. Phys. Lett. 1995, 247 (3), 310–312. 10.1016/0009-2614(95)01269-3.

[ref61] Atanasov M.; Ganyushin D.; Sivalingam K.; Neese F. A modern first-principles view on ligand field theory through the eyes of correlated multireference wavefunctions. Molecular Electronic Structures of Transition Metal Complexes II; Springer, 2011; Vol. 143, pp 149–220.

[ref62] Reinen D.; Atanasov M.; Lee S.-L. Second-sphere ligand field effects on oxygen ligator atoms and experimental evidence—the transition metal-oxygen bond in oxidic solids. Coord. Chem. Rev. 1998, 175 (1), 91–158. 10.1016/S0010-8545(98)00181-7.

[ref63] Volovik S. V.; Staninets V. I.; Zefirov N. S. Nature of ambivalence effects in chemical reactivity. Theor. Exp. Chem. 1991, 26 (4), 390–398. 10.1007/BF00530251.

[ref64] Sheldrick G. M. SHELXT - Integrated space-group and crystal-structure determination. Acta Crystallogr., Sect. A: Found. Adv. 2015, 71 (1), 3–8. 10.1107/S2053273314026370. 25537383 PMC4283466

[ref65] Sheldrick G. M. Crystal structure refinement with SHELXL. Acta Crystallogr., Sect. C: Struct. Chem. 2015, 71 (1), 3–8. 10.1107/S2053229614024218. 25567568 PMC4294323

[ref66] Spek A. L. PLATON SQUEEZE: a tool for the calculation of the disordered solvent contribution to the calculated structure factors. Acta Crystallogr., Sect. C: Struct. Chem. 2015, 71 (1), 9–18. 10.1107/S2053229614024929. 25567569

[ref67] X-RED; STOE & CIE GmbH: Darmstadt, Germany, 2002.

[ref68] SADABS; BRUKER AXS GmbH: Karlsruhe, Germany, 2016.

[ref69] Neese F. The ORCA program system. Wiley Interdiscip. Rev.: Comput. Mol. Sci. 2012, 2 (1), 73–78. 10.1002/wcms.81.

[ref70] Neese F. Software update: the ORCA program system, version 4.0. Wiley Interdiscip. Rev.: Comput. Mol. Sci. 2018, 8 (1), e1327 10.1002/wcms.1327.

[ref71] Neese F.; Wennmohs F.; Becker U.; Riplinger C. The ORCA quantum chemistry program package. J. Chem. Phys. 2020, 152 (22), 224108 10.1063/5.0004608. 32534543

[ref72] Siegbahn P.; Heiberg A.; Roos B.; Levy B. A Comparison of the Super-CI and the Newton-Raphson Scheme in the Complete Active Space SCF Method. Phys. Scr. 1980, 21 (3–4), 323–327. 10.1088/0031-8949/21/3-4/014.

[ref73] Siegbahn P. E. M.; Almlöf J.; Heiberg A.; Roos B. O. The complete active space SCF (CASSCF) method in a Newton-Raphson formulation with application to the HNO molecule. J. Chem. Phys. 1981, 74 (4), 2384–2396. 10.1063/1.441359.

[ref74] Roos B. O.; Taylor P. R.; Sigbahn P. E. M. A complete active space SCF method (CASSCF) using a density matrix formulated super-CI approach. Chem. Phys. 1980, 48 (2), 157–173. 10.1016/0301-0104(80)80045-0.

[ref75] Angeli C.; Cimiraglia R.; Evangelisti S.; Leininger T.; Malrieu J.-P. Introduction of n-electron valence states for multireference perturbation theory. J. Chem. Phys. 2001, 114 (23), 10252–10264. 10.1063/1.1361246.

[ref76] Angeli C.; Cimiraglia R. Multireference perturbation configuration interaction V. Third-order energy contributions in the Møller-Plesset and Epstein-Nesbet partitions. Theor. Chem. Acc. 2002, 107 (5), 313–317. 10.1007/s00214-002-0336-z.

[ref77] Angeli C.; Cimiraglia R.; Malrieu J.-P. N-electron valence state perturbation theory: a fast implementation of the strongly contracted variant. Chem. Phys. Lett. 2001, 350 (3–4), 297–305. 10.1016/S0009-2614(01)01303-3.

[ref78] Wolf A.; Reiher M.; Hess B. A. The generalized Douglas-Kroll transformation. J. Chem. Phys. 2002, 117 (20), 9215–9226. 10.1063/1.1515314. 15267790

[ref79] Hess B. A. Relativistic electronic-structure calculations employing a two-component no-pair formalism with external-field projection operators. Phys. Rev. A 1986, 33 (6), 3742–3748. 10.1103/PhysRevA.33.3742. 9897114

[ref80] Reiher M.; Wolf A. Exact decoupling of the Dirac Hamiltonian. II. The generalized Douglas-Kroll-Hess transformation up to arbitrary order. J. Chem. Phys. 2004, 121 (22), 10945–10956. 10.1063/1.1818681. 15634044

[ref81] Wolf A.; Reiher M. Exact decoupling of the Dirac Hamiltonian. III. Molecular properties. J. Chem. Phys. 2006, 124 (6), 064102 10.1063/1.2161179. 16483191

[ref82] Weigend F.; Ahlrichs R. Balanced basis sets of split valence, triple zeta valence and quadruple zeta valence quality for H to Rn: Design and assessment of accuracy. Phys. Chem. Chem. Phys. 2005, 7 (18), 3297–3305. 10.1039/b508541a. 16240044

[ref83] Schäfer A.; Horn H.; Ahlrichs R. Fully optimized contracted Gaussian basis sets for atoms Li to Kr. J. Chem. Phys. 1992, 97 (4), 2571–2577. 10.1063/1.463096.

[ref84] Schäfer A.; Huber C.; Ahlrichs R. Fully optimized contracted Gaussian basis sets of triple zeta valence quality for atoms Li to Kr. J. Chem. Phys. 1994, 100 (8), 5829–5835. 10.1063/1.467146.

[ref85] Pantazis D. A.; Chen X.-Y.; Landis C. R.; Neese F. All-Electron Scalar Relativistic Basis Sets for Third-Row Transition Metal Atoms. J. Chem. Theory Comput. 2008, 4 (6), 908–919. 10.1021/ct800047t. 26621232

[ref86] Neese F. A spectroscopy oriented configuration interaction procedure. J. Chem. Phys. 2003, 119 (18), 9428–9443. 10.1063/1.1615956.

[ref87] Neese F.; Lang L.; Chilkuri V. G. Topology, Entanglement, and Strong Correlations; Forschungszentrum Jülich GmbH, 2020.

[ref88] Maurice R.; Bastardis R.; Graaf C. d.; Suaud N.; Mallah T.; Guihéry N. Universal Theoretical Approach to Extract Anisotropic Spin Hamiltonians. J. Chem. Theory Comput. 2009, 5 (11), 2977–2984. 10.1021/ct900326e. 26609979

[ref89] Vallet V.; Maron L.; Teichteil C.; Flament J.-P. A two-step uncontracted determinantal effective Hamiltonian-based SO-CI method. J. Chem. Phys. 2000, 113 (4), 1391–1402. 10.1063/1.481929.

