# Supplementary material for: Oxygen-Donor Metalloligands Induce Slow Magnetization Relaxation in Zero Field for a Cobalt(II) Complex with {CoO4} Motif
Source: Inorg Chem. 2024 Mar 12;63(12):5652–63. doi: 10.1021/acs.inorgchem.4c00054 (PMC10966742; doi:10.1021/acs.inorgchem.4c00054)
Supplement: Supplementary file 1 — ic4c00054_si_001.pdf [file ic4c00054_si_001.pdf]

Supporting Information for

Oxygen-Donor Metalloligands Induce Slow Magnetization Relaxation in Zero Field for a Cobalt(II) Complex with $\{CoO_4\}$ Motif

Giuseppe Lococciolo,[a] Sandeep K. Gupta,[a],$ Sebastian Dechert,[a] Serhiy Demeshko,[a] Carole Duboc,[b] Mihail Atanasov,[c,d]* Frank Neese,[c]* and Franc Meyer[a]*

[a] Dr. G. Lococciolo, Dr. S. K. Gupta, Dr. S. Dechert, Dr. S. Demeshko, Prof. Dr. F. Meyer
University of Göttingen, Institute of Inorganic Chemistry
Tammannstraße 4, 37077 Göttingen, Germany

[b] Dr. Carole Duboc
Université Grenoble Alpes, CNRS UMR 5250, DCM
F-38000 Grenoble, France

[c] Prof. Dr. M. Atanasov, Prof. Dr. F. Neese
Max-Planck-Institut für Kohlenforschung
Kaiser-Wilhelm-Platz 1, 45470 Mülheim an der Ruhr, Germany

[d] Prof. Dr. M. Atanasov
Institute of General and Inorganic Chemistry, Bulgarian Academy of Sciences
Akad. Georgi Bontchev Street 11, 1113 Sofia, Bulgaria

* To whom correspondence should be addressed
E-mail: franc.meyer@chemie.uni-goettingen.de
E-mail: mihail.atanasov@kofo.mpg.de
E-mail: neese@kofo.mpg.de

$ New address: Department of Chemistry, Indian Institute of Technology Delhi, New Delhi, 110016, India

Table of Contents

1. Spectroscopic Characterization of the proligand $[H_4L(OTf)_2]$ S2
2. Spectroscopic Characterization of complex $[(L^{O,O}Ni)K(MeCN)(OTf)]$ (**1**)..........................S5
3. Spectroscopic Characterization of complex $[(L^{O,O}Ni)_2Co](OTf)_2$ (**2**).................................S8
4. Single Crystal Structure Determinations...S10
5. Magnetic Studies...S14
6. Theoretical Calculations and Analysis S22
7. References...S76

1. Spectroscopic Characterization of the proligand [H$_4$L(OTf)$_2$]

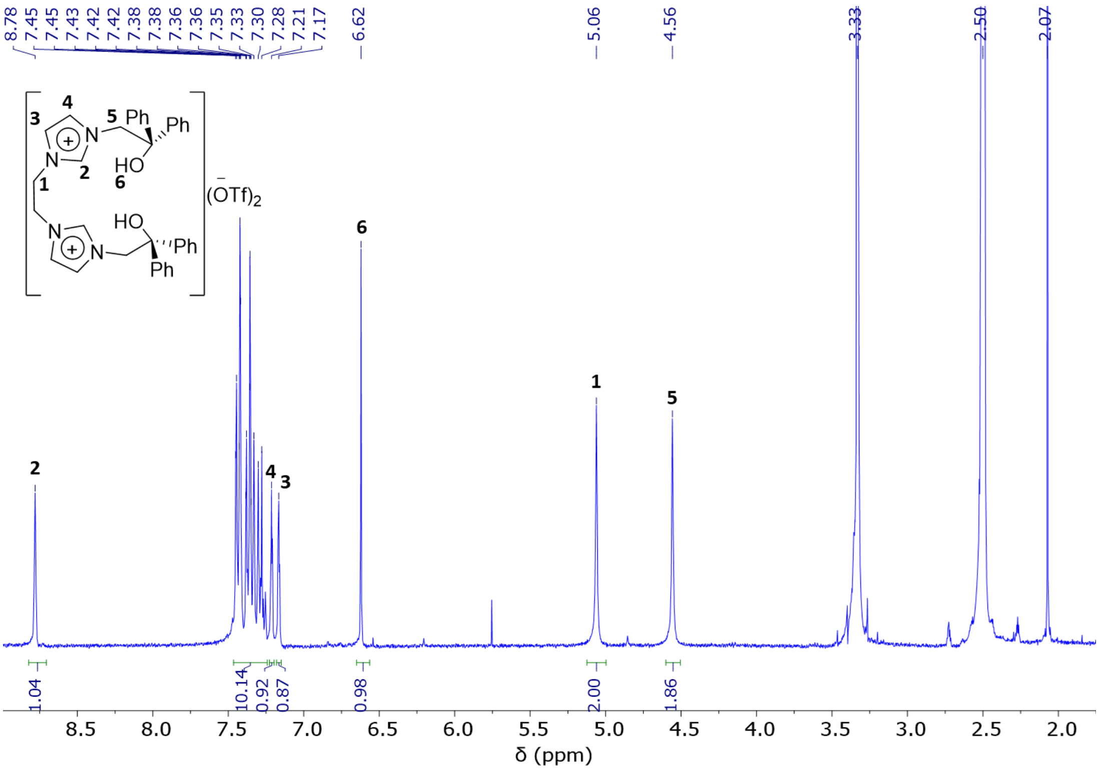

Figure S1. ^{1}H NMR spectrum of the proligand [H$_4$L^{O,O}](OTf)$_2$ in DMSO-d$_6$ at 295 K.

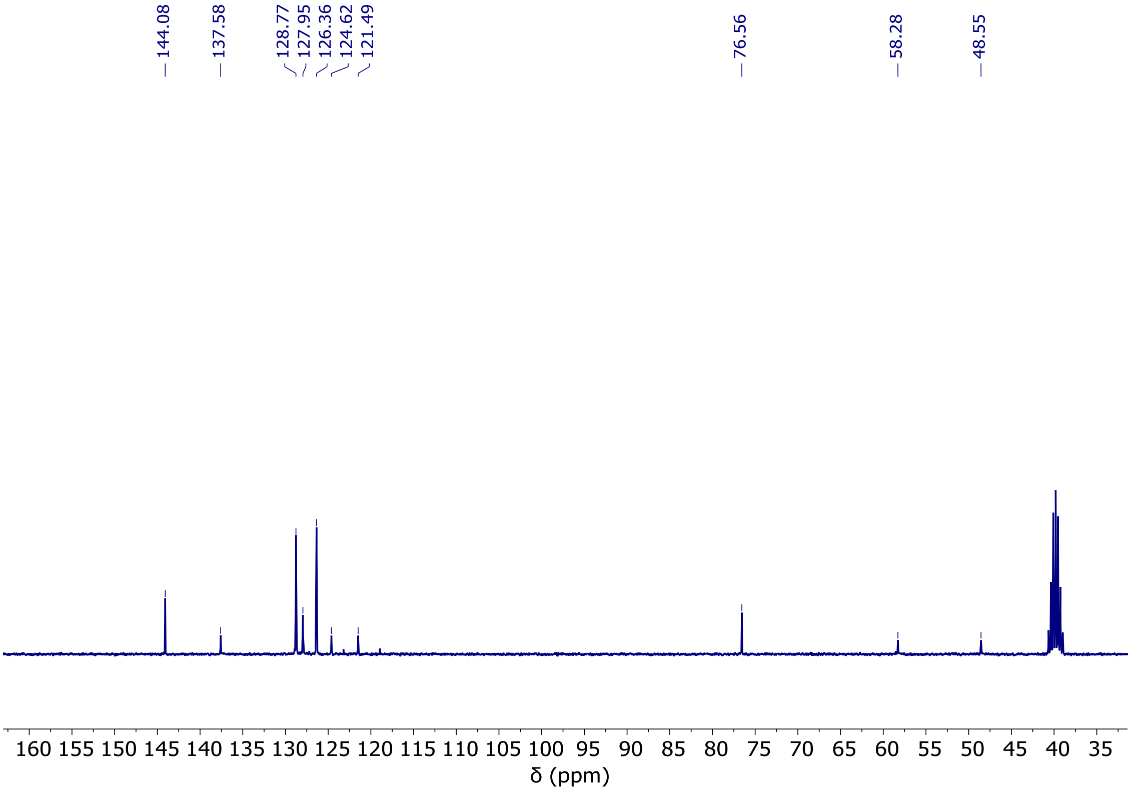

Figure S2. ^{13}C NMR spectrum of the proligand [H$_4$L^{O,O}](OTf)$_2$ in DMSO-d$_6$ at 295 K.

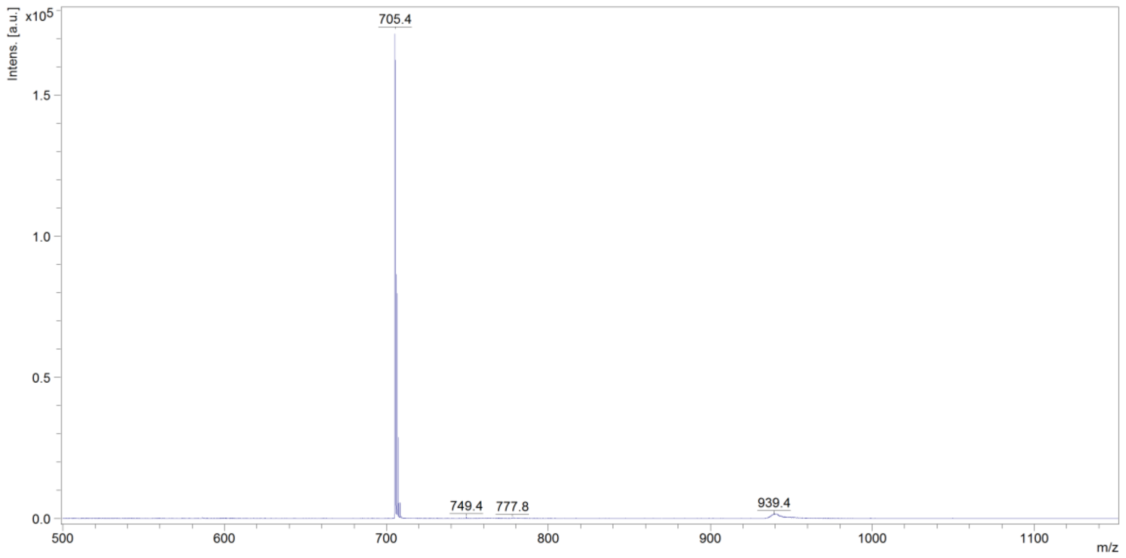

Figure S3. MALDI(+) mass spectrum of the proligand $[H_4L^{O,O}](OTf)_2$; *m*/*z* = 705.4 for the ion $[H_4L(OTf)]^+$.

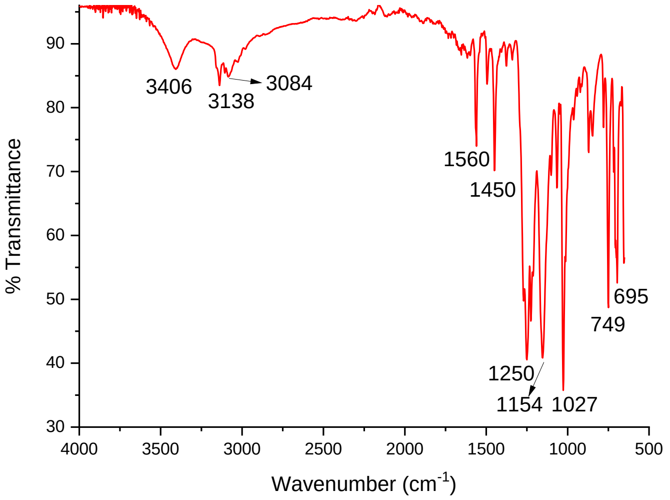

Figure S4. FTIR spectrum of the proligand $[H_4L^{O,O}](OTf)_2$.

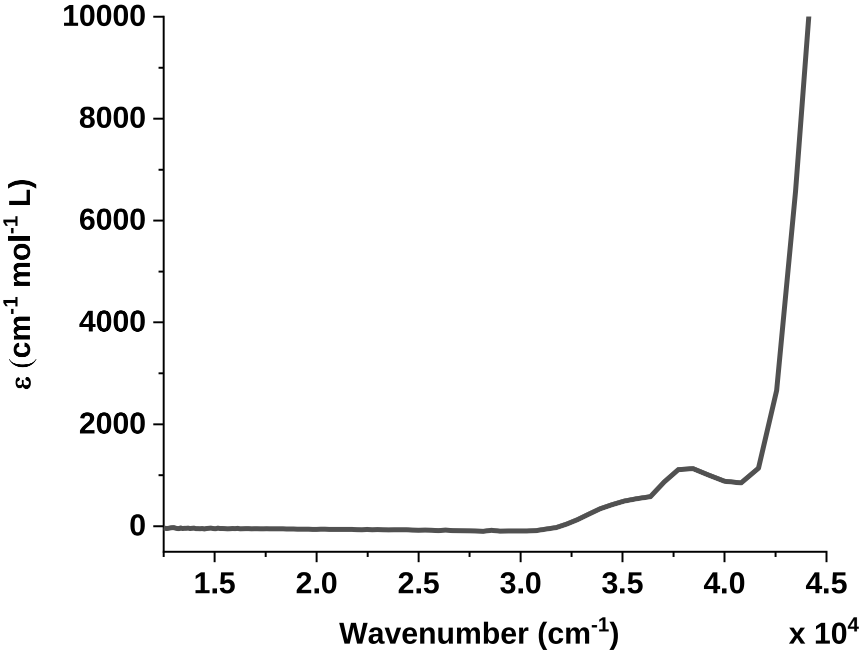

Figure S5. UV-Vis spectrum of the proligand $[H_4L^{O,O}](OTf)_2$ in MeCN.

2. Spectroscopic Characterization of complex [($L^{O,O}$Ni)K(MeCN)(OTf)] (1)

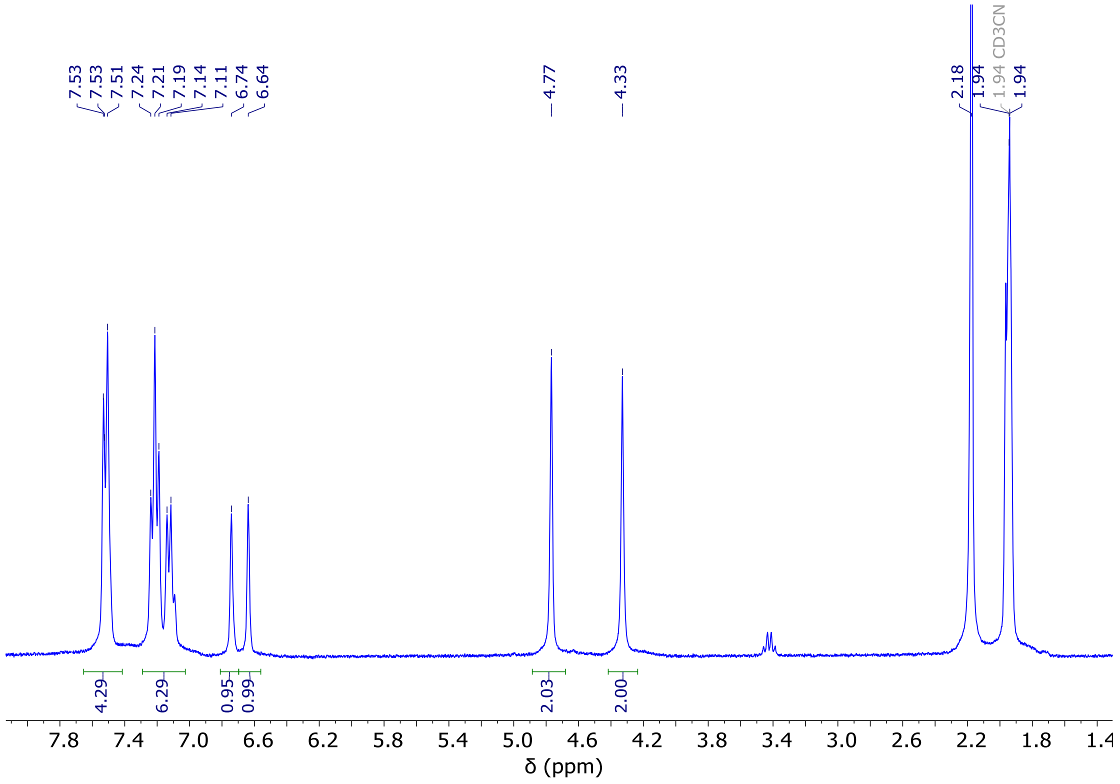

Figure S6. ^{1}H NMR spectrum of complex **1** in CD_3CN at 295 K.

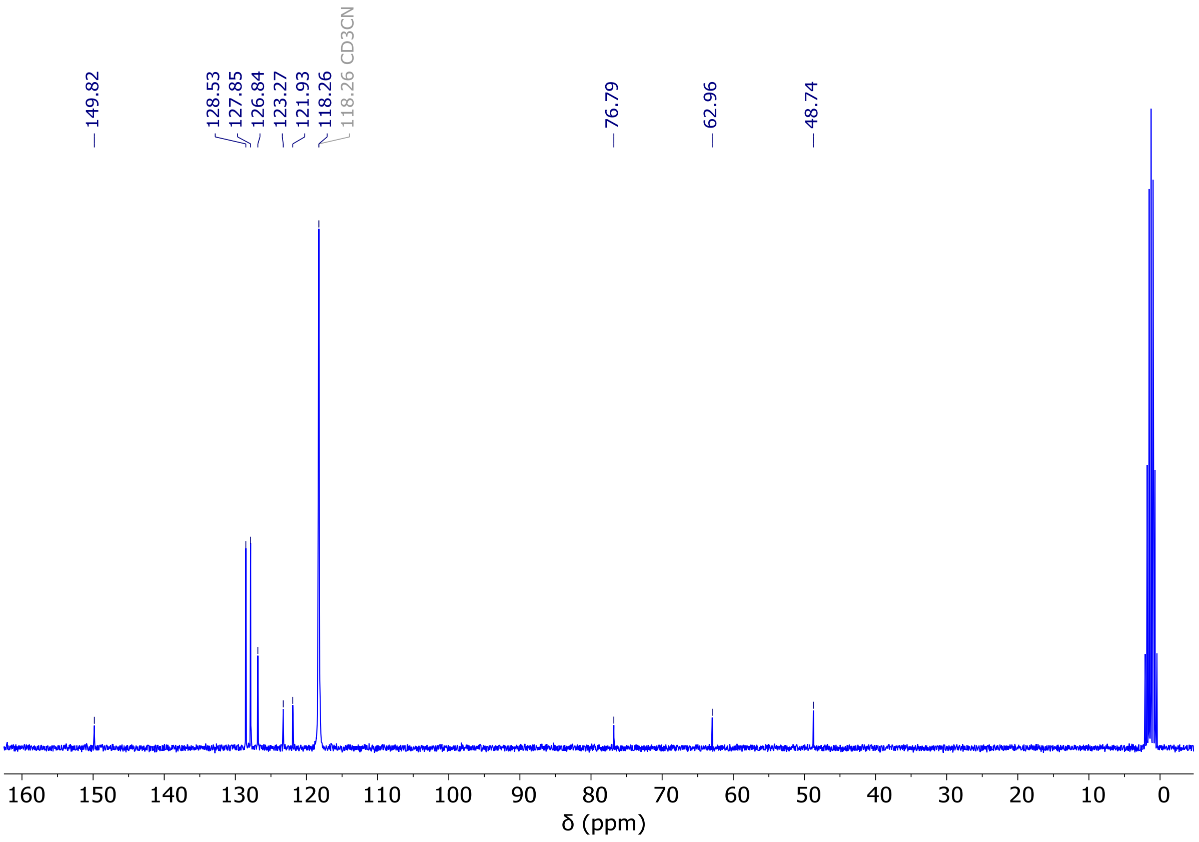

Figure S7. ^{13}C NMR spectrum of complex **1** in CD_3CN at 295 K.

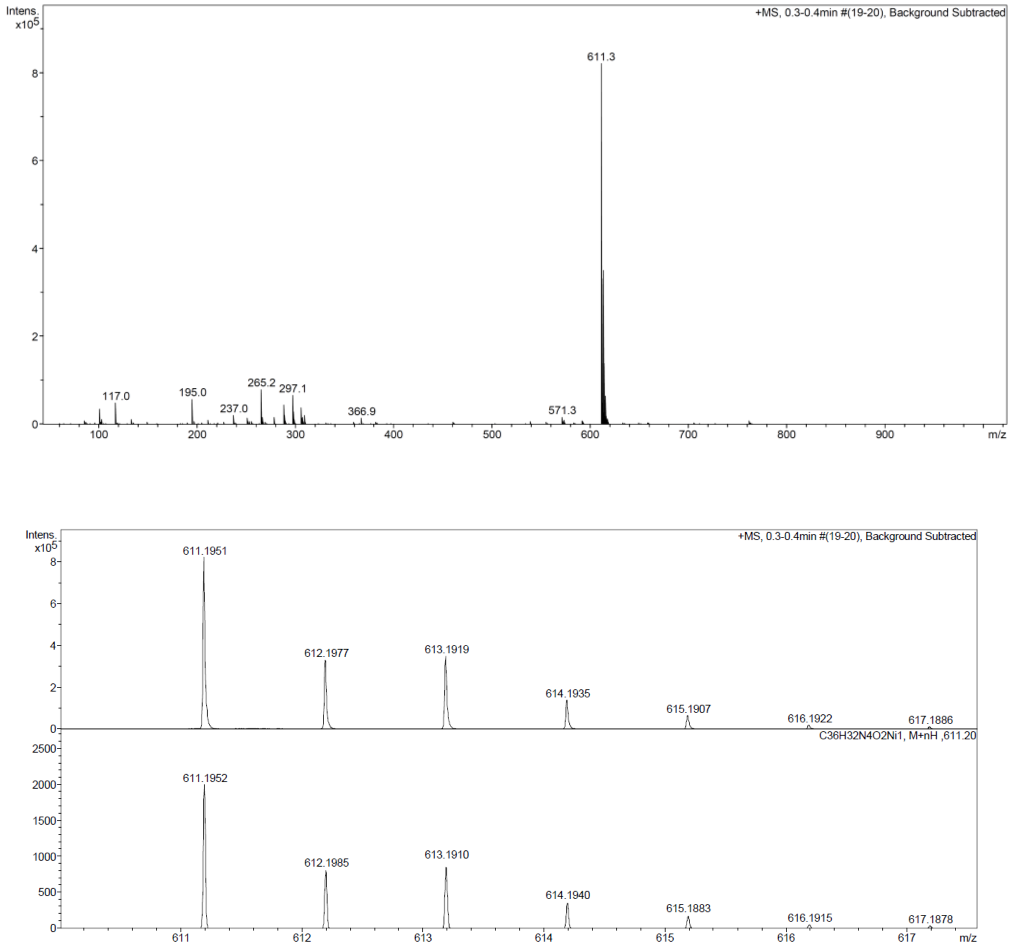

Figure S8. ESI(+) mass spectrum of complex **1** in MeCN; the lower part shows the experimental and simulated isotopic distribution pattern for the ion $[(L^{O,O}Ni)+H]^{+}$.

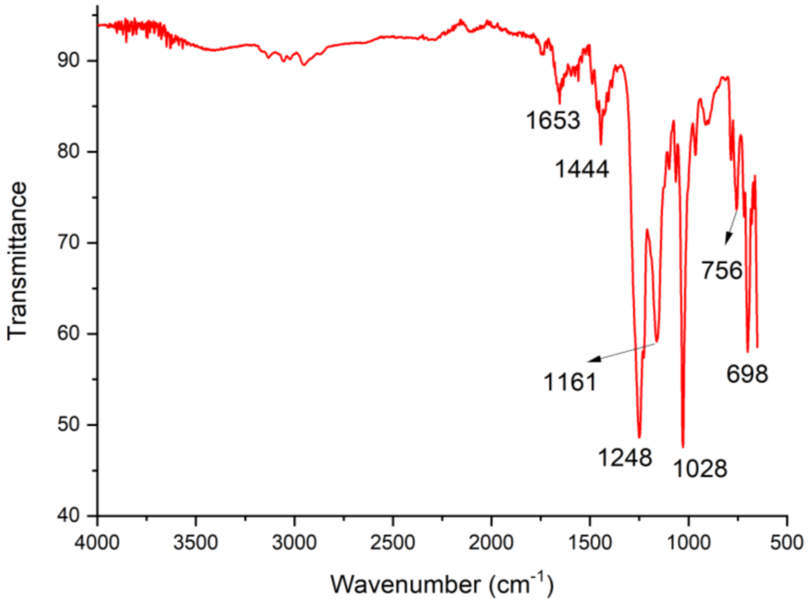

Figure S9. FTIR spectrum of complex **1.**

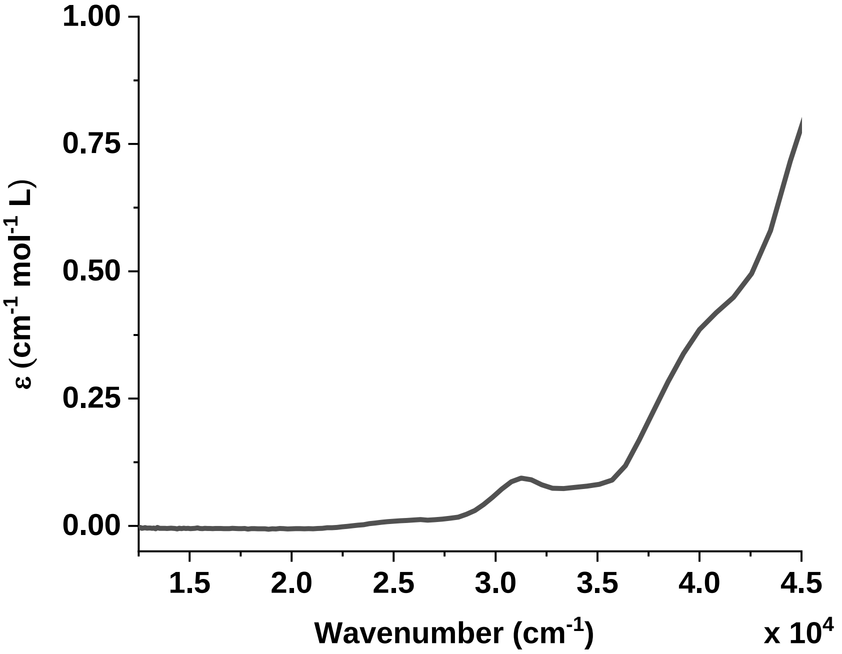

Figure S10. UV-Vis spectrum of complex **1** in MeCN.

3. Spectroscopic Characterization of complex $[(L^{O,O}Ni)_2Co](OTf)_2$ (2)

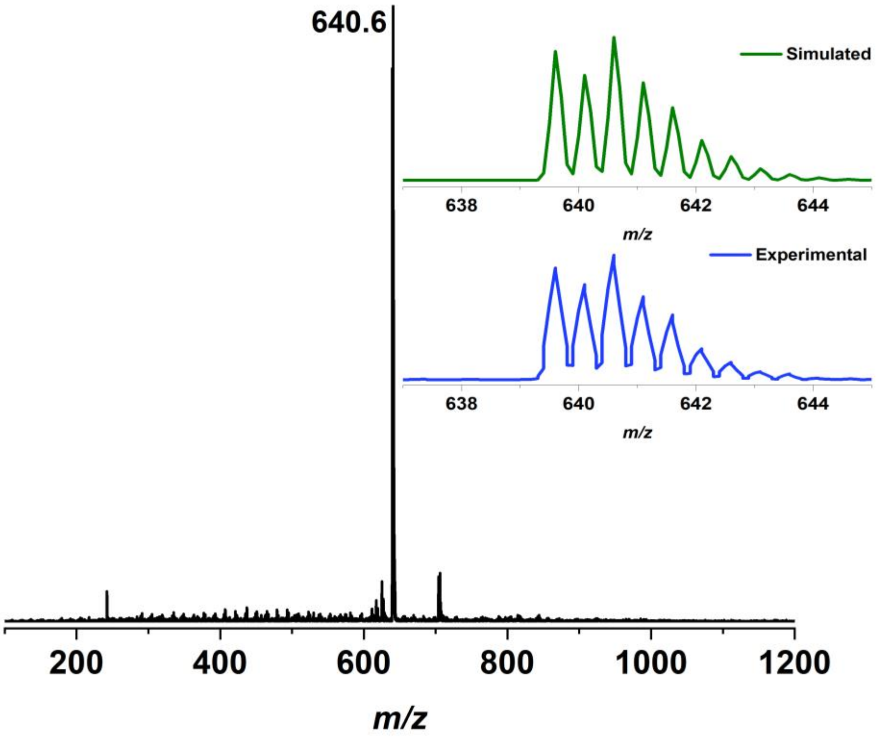

Figure S11. ESI(+) mass spectrum of complex **2** in MeCN. The inset shows the experimental and simulated isotopic distribution pattern for the ion $[(L^{O,O}Ni)_2Co]^{2+}$.

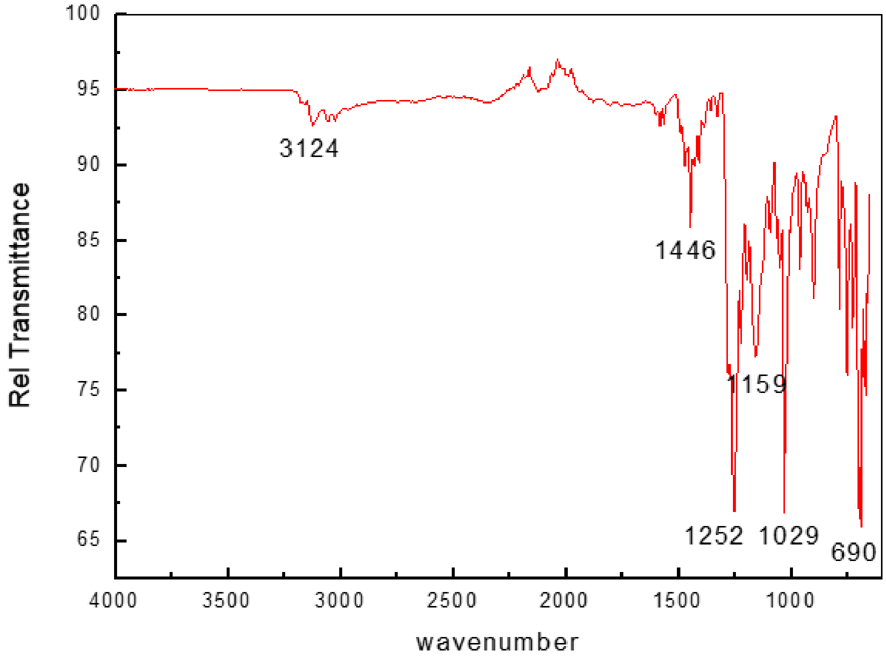

Figure S12. FTIR spectrum of complex **2.**

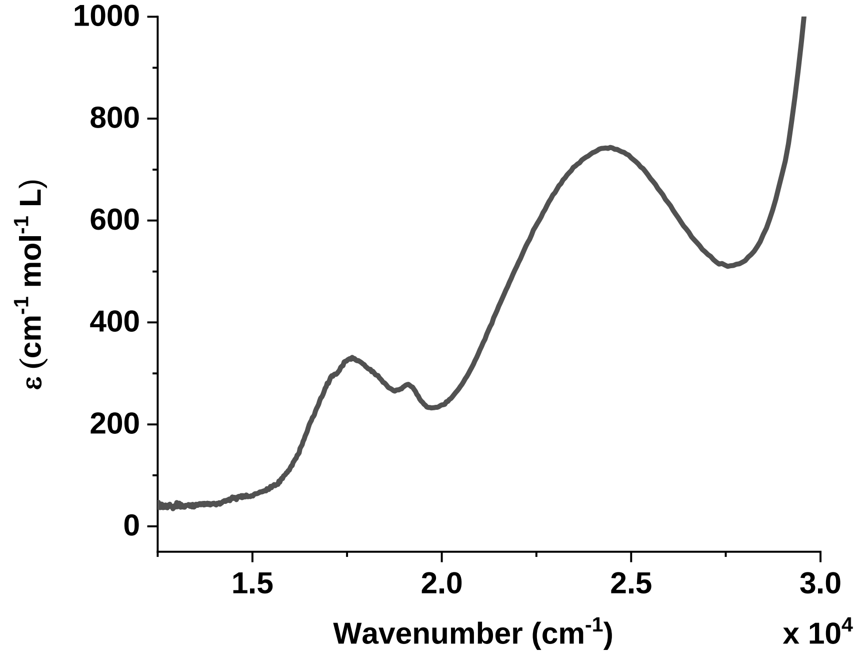

Figure S13. UV-Vis spectrum of complex **2** in MeCN.

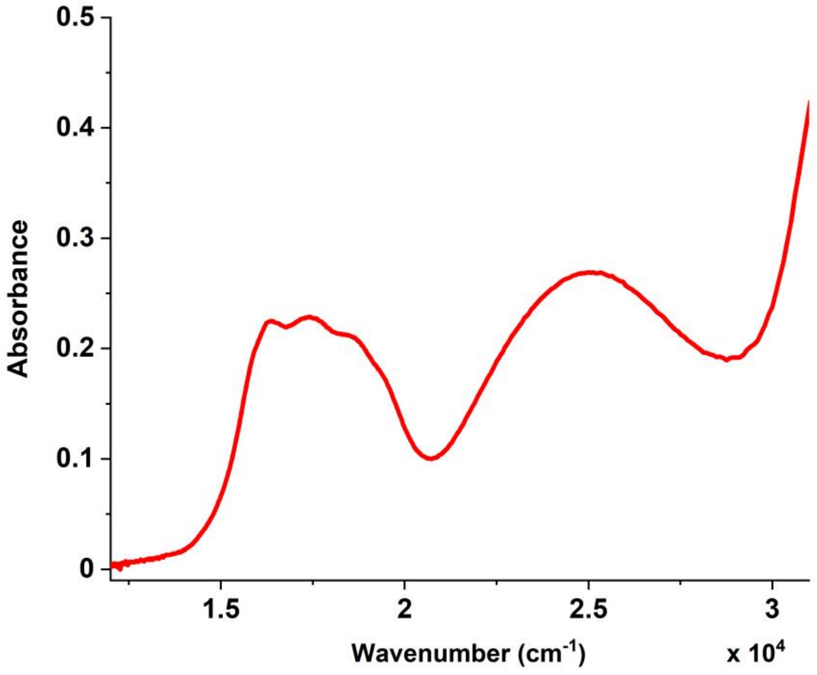

Figure S14. UV-Vis spectrum of a solid sample complex **2** (in KBr)

4. Single Crystal Structure Determinations

Table S1. Crystal data and refinement details for **1** and **2**.

**compound**	**1**	**2**
empirical formula	$C_{39}H_{35}F_3KN_5NiO_5S$	$C_{78}H_{70}CoF_6N_{10}Ni_2O_{10}S_2$
moiety formula	$C_{39}H_{35}F_3KN_5NiO_5S$	$C_{72}H_{64}CoN_8Ni_2O_4^{2+}$, $2(CF_3O_3S^-)$, $2(C_2H_3N)$
formula weight	840.59	1661.91
T [K]	133(2)	100(2)
crystal size [mm³]	0.50 x 0.36 x 0.34	0.49 x 0.34 x 0.12
crystal system	monoclinic	monoclinic
space group	$P2_1/c$ (No. 14)	$P2_1/c$ (No. 14)
a [Å]	11.4622(3)	15.7445(17)
b [Å]	18.2636(6)	16.2910(19)
c [Å]	17.9294(4)	32.842(4)
β [°]	93.256(2)	103.091(3)
V [Å³]	3747.30(18)	8204.9(16)
Z	4	4
ρ [g·cm⁻³]	1.490	1.345
F(000)	1736	3428
μ [mm⁻¹]	0.751	0.779
T_{min} / T_{max}	0.7314 / 0.8486	0.50 / 0.58
θ-range [°]	1.593 - 26.851	2.028 - 28.130
hkl-range	±14, ±23, -21 to 22	-19 to 20, ±21, ±43
measured refl.	51539	224074
unique refl. [R_{int}]	7965 [0.0203]	19861 [0.0882]
observed refl. ($I > 2\sigma(I)$)	7258	14041
data / restr. / param.	7965 / 0 / 497	19861 / 68 / 1015
goodness-of-fit (F^2)	1.042	1.026
*R*1, *wR*2 ($I > 2\sigma(I)$)	0.0297 / 0.0789	0.0728 / 0.1798
*R*1, *wR*2 (all data)	0.0336 / 0.0817	0.1056 / 0.2023
res. el. dens. [e·Å⁻³]	-0.503 / 0.566	-1.329 / 2.351

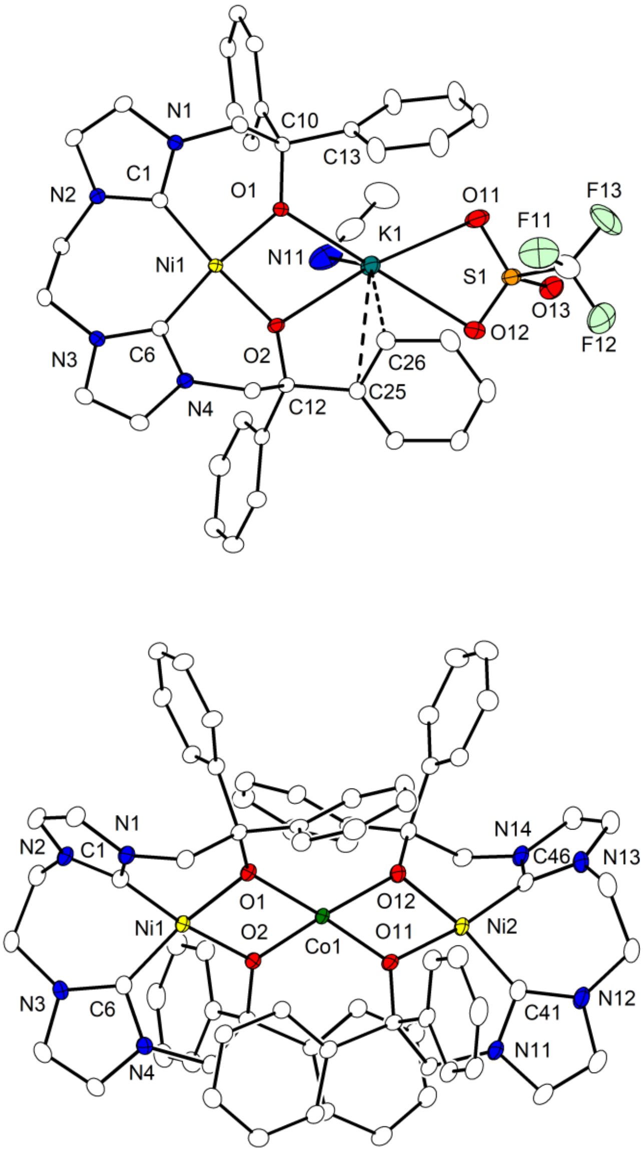

Figure S15. Plot (30% probability thermal ellipsoids) of the molecular structures of **1** (top) and the cationic part of **2** (bottom) (hydrogen atoms omitted for clarity).

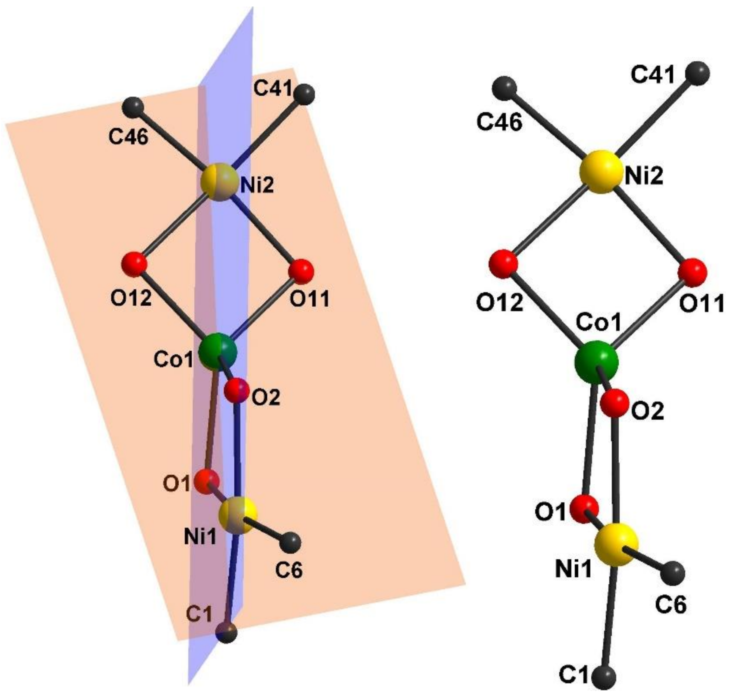

Figure S16. A view of the two intersecting O-Co-O planes in **2**.

Table S2. Selected bond distances (Å) and angles (º) in complex **1**.

Ni(1)-C(1)	1.8462(15)	K(1)-S(1)	3.3683(6)	Ni(1)-O(2)-K(1)	108.02(5)
Ni(1)-O(1)	1.8733(11)	K(1)-C(13)	3.4490(16)	O(1)-K(1)-O(2)	56.75(3)
Ni(1)-O(2)	1.8843(11)	K(1)-C(10)	3.5170(16)	O(1)-K(1)-N(11)	100.57(5)
Ni(1)-C(6)	1.8862(16)	K(1)-C(12)	3.5235(16)	O(2)-K(1)-N(11)	100.32(7)
K(1)-O(1)	2.5967(11)	C(1)-Ni(1)-C(6)	95.38(7)	O(1)-K(1)-O(12)	173.39(4)
K(1)-O(2)	2.6373(11)	O(1)-Ni(1)-C(6)	169.03(6)	O(2)-K(1)-O(12)	121.93(4)
K(1)-N(11)	2.812(2)	O(2)-Ni(1)-C(6)	91.93(6)	N(11)-K(1)-O(12)	86.03(6)
K(1)-O(12)	2.8306(14)	C(1)-Ni(1)-O(1)	90.85(6)	O(1)-K(1)-O(11)	132.15(4)
K(1)-O(11)	2.9083(15)	C(1)-Ni(1)-O(2)	170.56(6)	O(2)-K(1)-O(11)	170.39(4)
K(1)-C(26)	3.0891(18)	O(1)-Ni(1)-O(2)	82.92(5)	N(11)-K(1)-O(11)	75.49(7)
K(1)-C(25)	3.3078(16)	Ni(1)-O(1)-K(1)	110.01(5)	O(12)-K(1)-O(11)	49.76(4)

Table S3. Selected bond distances (Å) and angles (º) in complex **2**.

Ni(1)-C(1)	1.840(4)	Co(1)-O(12)	1.996(3)	C(6)-Ni(1)-O(2)	96.01(14)
Ni(1)-C(6)	1.882(4)	Co(1)-O(11)	1.996(3)	C(1)-Ni(1)-O(1)	92.04(15)
Ni(1)-O(2)	1.923(3)	O(2)-Co(1)-O(1)	79.58(11)	C(6)-Ni(1)-O(1)	168.90(16)
Ni(1)-O(1)	1.953(3)	O(2)-Co(1)-O(12)	130.02(13)	O(2)-Ni(1)-O(1)	82.18(11)
Ni(2)-C(46)	1.846(4)	O(1)-Co(1)-O(12)	125.02(11)	C(46)-Ni(2)-C(41)	90.40(17)
Ni(2)-C(41)	1.875(4)	O(2)-Co(1)-O(11)	121.62(11)	C(46)-Ni(2)-O(11)	172.45(14)
Ni(2)-O(11)	1.923(3)	O(1)-Co(1)-O(11)	128.09(12)	C(41)-Ni(2)-O(11)	96.03(15)
Ni(2)-O(12)	1.947(3)	O(12)-Co(1)-O(11)	79.69(11)	C(46)-Ni(2)-O(12)	91.89(15)
Co(1)-O(2)	1.989(3)	C(1)-Ni(1)-C(6)	90.56(17)	C(41)-Ni(2)-O(12)	167.05(17)
Co(1)-O(1)	1.992(3)	C(1)-Ni(1)-O(2)	172.51(14)	O(11)-Ni(2)-O(12)	82.72(11)

Table S4. SHAPE measures of complex **1**.[1]

Complex **1**	vTBPY-4	SS-4	T-4	SP-4
Ni1	27.89	14.765	27.003	0.67

Table S5. SHAPE measures of complex **2**.[1]

Complex **2**	vTBPY-4	SS-4	T-4	SP-4
Co1	9.698	9.272	6.757	22.793
Ni1	28.547	14.92	27.399	0.644
Ni2	27.626	14.125	26.461	0.782

5. Magnetic Studies

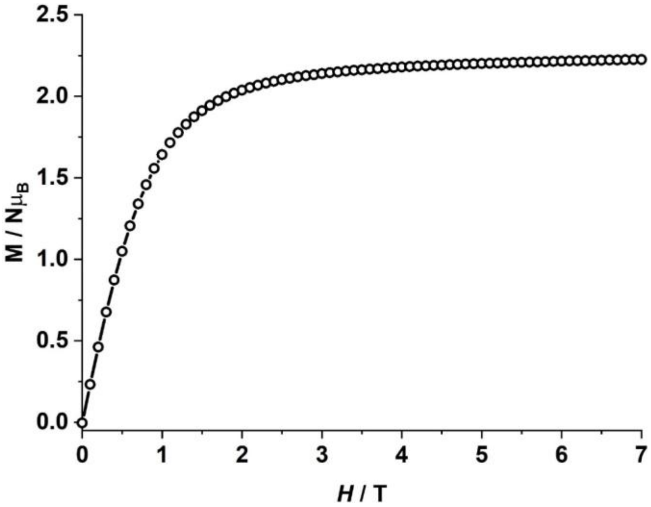

Figure S17. Variable field magnetization at 2.0 K for **2**.

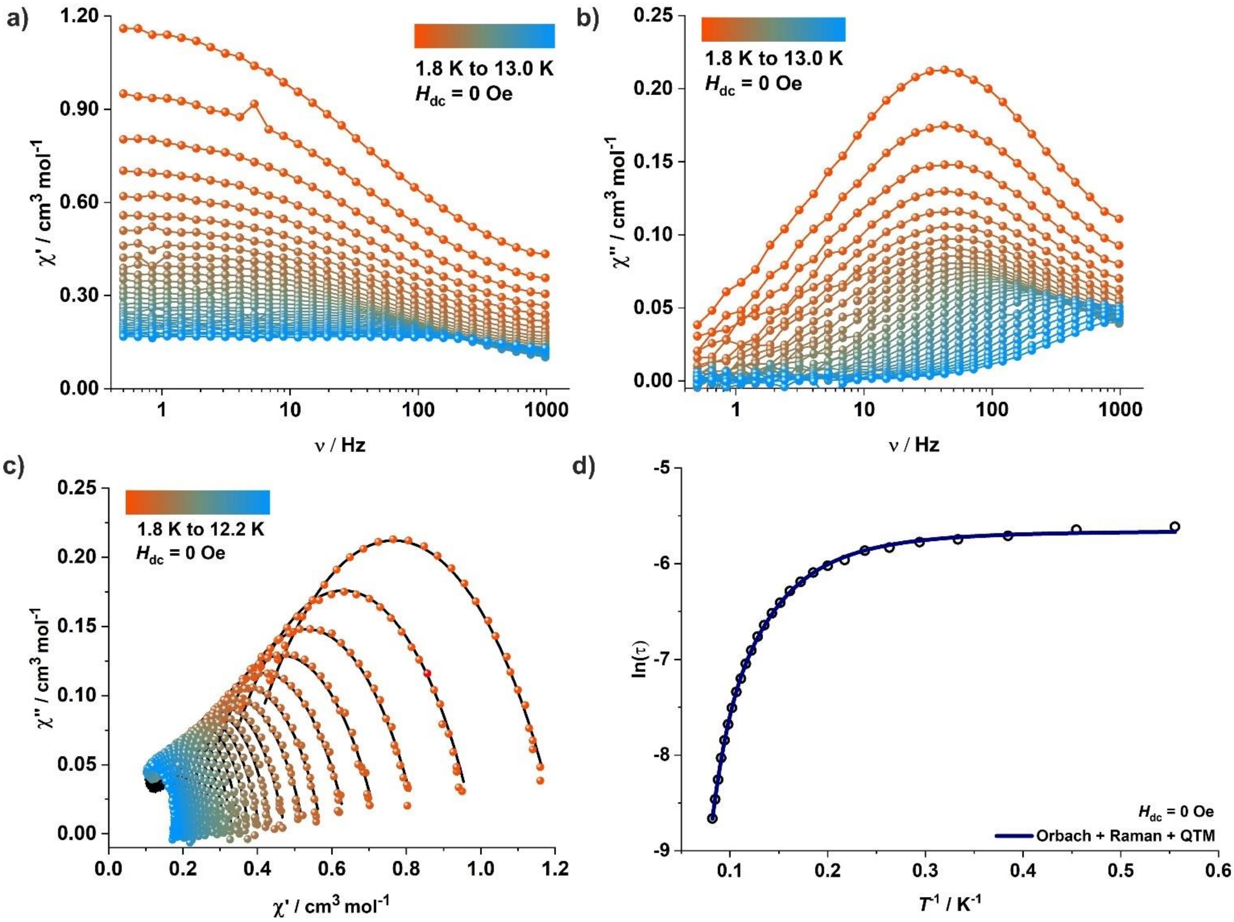

Figure S18. (a) In-phase (χ_M') and (b) out-of-phase (χ_M'') component of the frequency-dependent (0.1–1000 Hz) ac susceptibility measured in an oscillating ac field of 3.0 Oe under zero dc field for complex **2**. (c) Cole-Cole plots for complex **2** under zero field. (d) The plot of the relaxation time τ *versus* T^{-1}. The solid blue line represents the best fit to the relaxation via a combination of Orbach, Raman and QTM relaxation pathways [U_{eff} = 125 K (86.9 cm^{-1}), τ_0 = 1.32 × 10^{-8} s; C = 0.403 s^{-1} K^{-n}, n = 3.54; τ_{QTM} = 0.00349 s].

Table S6. Parameters obtained by fitting the ac susceptibility data for **2** under zero applied dc field.

T (K)	τ	χ_S	χ_T	α	Residual
1.8	3.65E-03	3.24E-01	1.21E+00	4.30E-01	5.66E-05
2.2	3.53E-03	2.73E-01	9.87E-01	4.17E-01	1.46E-04
2.6	3.32E-03	2.30E-01	8.35E-01	4.20E-01	6.19E-05
3.0	3.19E-03	2.01E-01	7.27E-01	4.19E-01	6.36E-05
3.4	3.10E-03	1.84E-01	6.44E-01	4.04E-01	7.50E-05
3.8	2.93E-03	1.68E-01	5.76E-01	3.91E-01	7.51E-05
4.2	2.84E-03	1.52E-01	5.28E-01	3.90E-01	8.16E-05
4.6	2.58E-03	1.46E-01	4.75E-01	3.53E-01	9.54E-05
5.0	2.43E-03	1.37E-01	4.39E-01	3.41E-01	7.44E-05
5.4	2.26E-03	1.29E-01	4.08E-01	3.25E-01	6.99E-05
5.8	2.05E-03	1.23E-01	3.79E-01	3.01E-01	6.33E-05
6.2	1.86E-03	1.18E-01	3.54E-01	2.79E-01	6.01E-05
6.6	1.65E-03	1.12E-01	3.32E-01	2.62E-01	5.18E-05
7.0	1.48E-03	1.08E-01	3.13E-01	2.35E-01	4.53E-05
7.4	1.30E-03	1.03E-01	2.96E-01	2.23E-01	3.86E-05
7.8	1.16E-03	9.88E-02	2.81E-01	2.06E-01	3.45E-05
8.2	1.00E-03	9.54E-02	2.65E-01	1.76E-01	2.85E-05
8.6	8.71E-04	8.95E-02	2.54E-01	1.75E-01	2.58E-05
9.0	7.46E-04	8.59E-02	2.42E-01	1.55E-01	2.09E-05
9.4	6.49E-04	8.20E-02	2.32E-01	1.43E-01	1.74E-05
9.8	5.49E-04	7.73E-02	2.22E-01	1.38E-01	1.50E-05
10.2	4.62E-04	7.43E-02	2.14E-01	1.28E-01	1.38E-05
10.6	3.92E-04	7.20E-02	2.06E-01	1.19E-01	1.12E-05
11.0	3.25E-04	6.96E-02	1.99E-01	1.18E-01	1.15E-05
11.4	2.60E-04	6.43E-02	1.92E-01	1.23E-01	1.04E-05
11.8	2.11E-04	6.23E-02	1.84E-01	1.16E-01	1.54E-05
12.2	1.73E-04	5.88E-02	1.80E-01	1.36E-01	1.42E-05

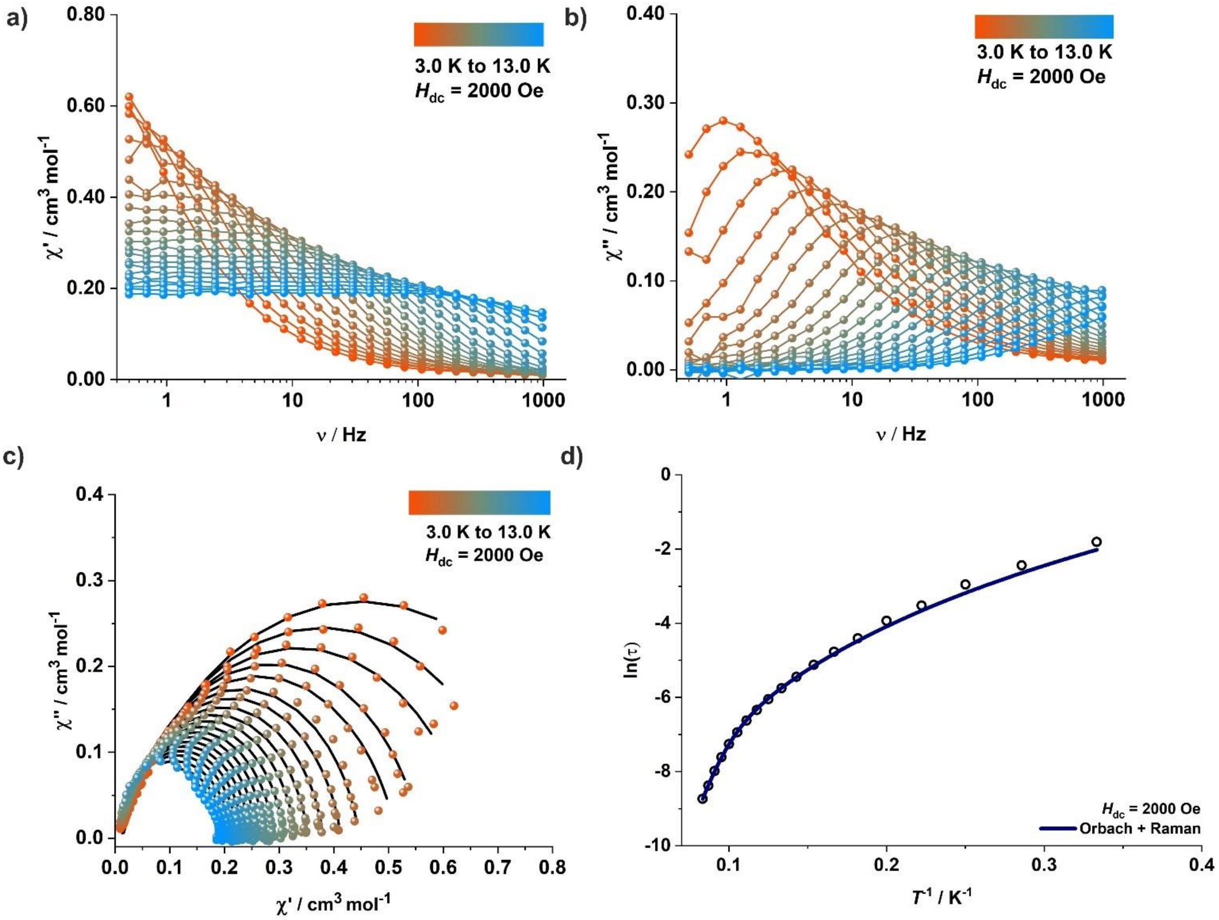

Figure S19. (a) In-phase (χ_M') and (b) out-of-phase (χ_M'') component of the frequency-dependent (0.1–1000 Hz) ac susceptibility measured in an oscillating ac field of 3.0 Oe under an applied dc field of 2000 Oe for complex **2**. (c) Cole-Cole plots for complex **2** under an applied dc field of 2000 Oe. (d) The plot of the relaxation time τ *versus* T^{-1}. The solid blue line represents the best fit to the relaxation via a combination of Orbach and Raman relaxation pathways [U_{eff} = 134 K (93.1 cm^{-1}), τ_0 = 3.40 · 10^{-9} s; C = 0.088 s^{-1} K^{-n}, n = 4.05].

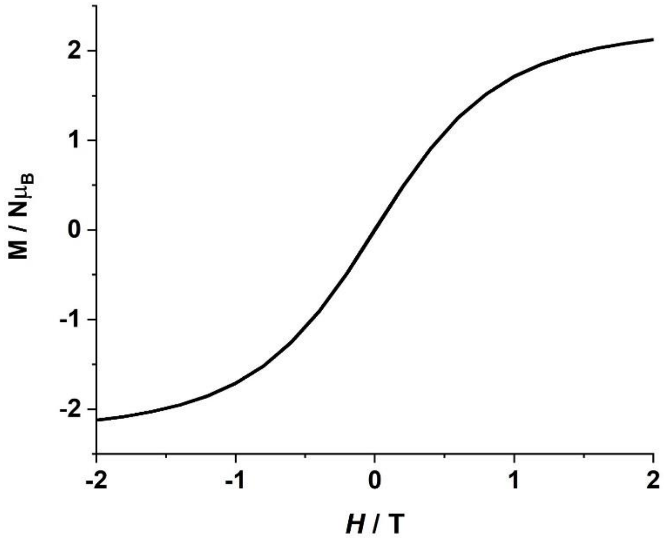

Figure S20. Variable field magnetization for **2** at a sweep rate of 100 Oe/s at 1.8 K.

Table S7. Parameters obtained by fitting the ac susceptibility data for **2** under an applied dc field of 2000 Oe.

T (K)	τ	χ_S	χ_T	α	Residual
3	1.65E-01	1.06E-02	8.87E-01	2.85E-01	9.22E-04
3.5	8.74E-02	1.28E-02	7.47E-01	2.50E-01	1.97E-03
4	5.21E-02	1.06E-02	6.58E-01	2.36E-01	1.39E-03
4.5	2.96E-02	1.41E-02	5.60E-01	1.86E-01	1.54E-03
5	1.95E-02	1.26E-02	5.15E-01	1.80E-01	4.06E-03
5.5	1.22E-02	1.45E-02	4.47E-01	1.39E-01	2.70E-03
6	8.51E-03	1.31E-02	4.13E-01	1.29E-01	8.27E-04
6.5	5.97E-03	1.19E-02	3.81E-01	1.19E-01	6.08E-04
7	4.31E-03	1.21E-02	3.53E-01	1.07E-01	5.91E-04
7.5	3.17E-03	1.14E-02	3.30E-01	9.55E-02	4.56E-04
8	2.36E-03	1.04E-02	3.08E-01	8.58E-02	4.05E-04
8.5	1.76E-03	1.02E-02	2.90E-01	7.59E-02	4.08E-04
9	1.33E-03	9.61E-03	2.74E-01	6.82E-02	3.26E-04
9.5	9.67E-04	7.56E-03	2.60E-01	7.07E-02	7.60E-04
10	7.05E-04	6.25E-03	2.48E-01	7.19E-02	2.02E-04
10.5	4.93E-04	6.62E-03	2.35E-01	7.10E-02	1.44E-04
11	3.40E-04	3.24E-03	2.26E-01	8.68E-02	2.07E-04
11.5	2.30E-04	2.89E-03	2.15E-01	1.02E-01	3.41E-04
12	1.61E-04	9.45E-03	2.06E-01	1.17E-01	2.06E-04
12.5	1.02E-04	1.96E-03	1.99E-01	1.53E-01	1.80E-04
13	8.48E-05	2.16E-02	1.91E-01	1.39E-01	1.84E-04

Table S8. Selected examples of four-coordinate Co(II) SIMs reported in the literature.

**Complex Donor Atoms**	**Donor Atoms**	**Bite Angle (°)**	**Dihedral Angle (°)**	***D*** **(cm^{-1})**	***E/D*** **(cm^{-1})**	***H***$_{dc}$ **(Oe)**	***U*****$_{eff}$ (cm^{-1})**	**τ_0 (s)**	**Reference**
$[LNiCoNiL](OTf)_2$ (**2**)	$\{CoO_4\}$	79.53	84.32	-74.3	0	0	86.9	1.32×10^{-8}	***This work***
						2000	93.1	3.40×10^{-9}	***This work***
$(Ph_4P)_2[Co(OPh)_4]\cdot(CH_3CN)$	$\{CoO_4\}$	107.77	84.30	-11.1	0	1400	21	7.0×10^{-10}	2
$K(Ph_4P)[Co(OPh)_4]$	$\{CoO_4\}$	104.9	85.77	-23.8	0	-	-	-	2
$K(Ph_4P)[Co_{0.06}Zn_{0.94}(OPh)_4]$						0	34.0	1.0×10^{-9}	2
$[Co^{II}Co^{III}{}_4L^1{}_2(\mu\text{-}OH)_2(\mu_{1,3}\text{-}O_2CCH_3)_2](ClO_4)_4\cdot H_2O$	$\{CoO_4\}$	101.15	80.28	-23.6	0.03	1000	20.8	9.1×10^{-8}	3
$[Co^{II}Co^{III}{}_4L^1{}_2(\mu\text{-}OH)_2(\mu_{1,3}\text{-}O_2CC_2H_5)_2](ClO_4)_4\cdot H_2O$	$\{CoO_4\}$	98.45	80.53	-24.3	0	1000	22.9	4.3×10^{-8}	3
$[Co^{II}Co^{III}{}_4L^2{}_2(\mu_{1,3}\text{-}O_2CCH_3)_2(\mu\text{-}OH)_2](ClO_4)_4\cdot 4H_2O$	$\{CoO_4\}$	94.05	79.5	-31.3	0.11	500	37.5	3.6×10^{-9}	4
$[Co^{II}Co^{III}{}_4L^2{}_2(\mu_{1,3}\text{-}O_2CC_2H_5)_2(\mu\text{-}OH)(\mu\text{-}OMe)](ClO_4)_4\cdot 5H_2O$	$\{CoO_4\}$	96.15	82.3	-21.9	0.08	3000	15.4	4.7×10^{-7}	4
$[TBA]_2[L^3{}_2Co]$	$\{CoN_4\}$	83.75	87.88	-113	0	0	226	1.46×10^{-10}	5
$[TBA]_2[L^3{}_2Co]$ (frozen solution)						0	226	7.38×10^{-11}	5
$(HNEt_3)_2[Co(bmsab)_2]$	$\{CoN_4\}$	80.65	85.19	−115	0	0	230	7.63×10^{-11}	6
$K_2[Co(bmsab)_2]$	$\{CoN_4\}$	80.72, 80.41	83.28, 87.30	−100	0	0	200	3.03×10^{-9}	7
$(HNEt_3)_2[Co(btsab)_2]$	$\{CoN_4\}$	81.30	84.03	−110	0	0	220	1.1×10^{-10}	7
$[K(18C6)]_2[Co(bmsab)_2]$	$\{CoN_4\}$	81.05	86.62	−130	0	0	260	5.0×10^{-9}	7
$[Co\{(N^tBu)_3SMe\}_2]$	$\{CoN_4\}$	71.46	87.47	- 81.3	0	0	159	6.09×10^{-10}	8-9
$[Co\{(N^tBu)_2SPh\}_2]$	$\{CoN_4\}$	72.65	78.74	- 114	0	0	283	2.67×10^{-9}	9
$[Co\{(N^tBu)_3SPh\}_2]$	$\{CoN_4\}$	70.83	88.52	- 75.5	0	0	213	1.76×10^{-11}	9
$[Co\{(N^tBu)_3SCH_2PPh_2\}_2]$	$\{CoN_4\}$	71.40	85.42	- 79.3	0	0	199	3.08×10^{-11}	9
$(HNEt_3)_2[Co(L^4)_2]\cdot H_2O$	$\{CoN_4\}$	81.32	87.10	−144.1	0.0	0	46.0	5.40×10^{-6}	10
$(Bu_4N)_2[Co(L^5)_2]\cdot H_2O$	$\{CoN_4\}$	83.38	87.49	−130.8	0.005	0	58.4	2.47×10^{-6}	10
$(HNEt_3)_2[CoL^6]$	$\{CoN_4\}$	81.36, 81.86	89.32, 88.87	-128.2	0.005	0	30.5	1.13×10^{-5}	11
$Co[R_1(C_6N_2H_5)R_2]_2$	$\{CoN_4\}$	81.86	71.31	-58.5	0	2600	117	8.96×10^{-10}	12
$Co[R_3(C_6N_2H_5)R_4]_2$	$\{CoN_4\}$	81.74	76.35	-91.9	0	0	183.8	1.96×10^{-10}	12

$Co[R_5(C_6N_2H_5)R_6]_2$	$\{CoN_4\}$	81.79	82.06	-64.5	0	0	129	6.53×10^{-10}	12
$Co[R_7(C_6N_2H_5)R_8]_2$	$\{CoN_4\}$	82.14	89.10	-57.7	0	0	115.4	6.77×10^{-9}	12
$Co[R_9(C_6N_2H_5)R_{10}]_2$	$\{CoN_4\}$	82.15	83.66	-54.1	0	0	108.2	7.01×10^{-9}	12
$Co[R_{11}(C_6N_2H_5)R_{12}]_2$	$\{CoN_4\}$	81.79	85.78	-50.5	0	0	101	8.14×10^{-9}	12
$[Co(half\text{-}Pc)_2]$	$\{CoN_4\}$	91.02	89.96	-27.9	0	0	54.0	3.17×10^{-10}	13
$[CoL^7{}_2](ClO_4)_2$	$\{CoN_4\}$	83.95	69.38	-41.2	0.18	1000	46.9	1.96×10^{-8}	14
$(Bu_4N)_2[Co(C_3S_5)_2]$	$\{CoS_4\}$	94.09	76.50	-187	0	0	-	-	15
$(Ph_4P)_2[Co(C_3S_5)_2]$	$\{CoS_4\}$	94.05	79.70	-161	0	0	33.9	4.5×10^{-6}	15-16
$(PPN)_2[Co(C_3S_5)_2]$	$\{CoS_4\}$	93.21	81.82	-177	0	0	-	-	15
$[K(18C6)]_2[Co(C_3S_5)_2]$	$\{CoS_4\}$	93.95	83.08	-166	0	0	-	-	15
$C_{16}H_{52}B_{20}CoN_2S_4$	$\{CoS_4\}$	95.59	89.53	-71.6	0.0038	0	26.8	3.3×10^{-6}	17
$(Ph_4P)_2[Co(SPh)_4]$	$\{CoS_4\}$	95.6	93.8	-62.0	0	0	21	1.0×10^{-6}	2, 18-19
$[Co(L^8)_4](NO_3)_2$	$\{CoS_4\}$	91.52, 91.03	78.12/83.34	-61.7	0	0	19.5	7.59×10^{-7}	20
$[Co(L^9)_4](ClO4)_2$	$\{CoS_4\}$	95.66	88.24	-80.7	0	0	32.0	2.24×10^{-6}	20
$[Co(L^{10})_4](ClO4)_2$	$\{CoS_4\}$	99.23, 99.43	87.67/89.75	-70.8	0	2000	18.7	1.55×10^{-6}	20
$[Co(L^{11})_4](ClO4)_2$	$\{CoS_4\}$	104.72	85.49	-21.3	0	2000	13.2	3.21×10^{-8}	20
$[Co(L^{12})_4]Br_2$	$\{CoS_4\}$	103.05	87.79	-5.9	0.06	-	-	-	21
$[Co(L^{12})_4]I_2$	$\{CoS_4\}$	103.80	88.97	-5.1	0.06	-	-	-	21
$[Co(L^{12})_4]((SiF_6)$	$\{CoS_4\}$	106.53	87.36	-12.2	0.16	0	34.8	5×10^{-7}	21
$(Ph_4P)_2[Co(SePh)_4]$	$\{CoSe_4\}$	94.3	86.29	-83.0	0	0	19	3.0×10^{-6}	2
$Co[(TeP^iPr_2)_2N]_2$	$\{CoTe_4\}$	104.97	89.75	-45.1	0.10	0	16	2×10^{-7}	22

Bite angle = X-Co-X angles for chelating ligands. In the case of monodentate ligands, the smallest X-Co-X angles have been considered as bite angles; Dihedral angle = angle between planes defined by X-Co-X of the respective chelating ligands. In the case of monodentate ligands, the dihedral angle is defined by the angle between planes with the smallest bite angles. H_3L^1 = 2,6-bis-[{2-(2-hydroxyethylthio)ethylimino}methyl]-4-methylphenol; H_3L^2 = 2,6-bis((2-(2-hydroxyethylamino)ethylimino)methyl)-4-methylphenol; H_2L^3 = N,N'-bis(4-chlorophenyl)oxanilide; bmsab = 1,2-bis(methanesulfonamido)benzene; btsab = 1,2-bis(toluenesulfonamido)benzene; H_2L^4 = N,N′-bis(p-toluenesulfony1)oxamide; H_2L^5 = N,N′-diphenyloxamide; H_2L^6 = N,N′-bis(methanesulfonyl)oxamide; R_1 = H, R_2 = 4-*tert*-butylphenylsulfonyl; R_3 = H, R_4 = 5-(dimethylamino)naphthalen-1-ylsulfonyl; R_5 = H, R_6 = mesitylsulfonyl; R_7 = H, R_8 = tosyl; R_9 = H, R_{10} = naphthalen-1-ylsulfonyl; R_{11} = Me, R_{12} = 4-tert-butylphenylsulfonyl; L^7 = 2,9-diphenyl-1,10-phenanthroline; $C_3S_5{}^{2-}$ = 4,5-dimercapto-1,3-dithiole-2-thionate; L^8 = thiourea, L^9 = 1,3-dibutylthiourea, L^{10} = 1,3-phenylethylthiourea, L^{11} = 1,1,3,3-tetramethylthiourea); L^{12} = thiourea

Table S9. Selected examples of prominent two to six-coordinate cobalt and iron-based SIMs reported in the literature.

**Complex**	**Coordination Number**	**Donor Atoms**	***D*** **(cm^{-1})**	***E*/*D*** **(cm^{-1})**	***H*$_{dc}$ (Oe)**	***U*$_{eff}$ (cm^{-1})**	**τ_o (s)**	**Reference**
[Co(C(SiMe$_2$ONaph)$_3$)$_2$]	2	{CoC$_2$}	-	-	0	450	1.79×10^{-9} s	***23***
[(IPr)CoNDmp]	2	{CoCN}	-	-	0	297	7.5×10^{-11}	***24***
[(cyIPr)CoNDmp]	2	{CoCN}	-	-	0	288	8.4×10^{-10}	***24***
[(sIPr)CoNDmp]	2	{CoCN}	-	-	0	413	1.2×10^{-10}	***24***
Fe[C(SiMe$_3$)$_3$]$_2$	2	{FeC$_2$}	-	-	500	146	4×10^{-9}	***25***
[K(crypt-222)][Fe(C(SiMe$_3$)$_3$)$_2$]	2	{FeC$_2$}	-	-	0	226	1.3×10^{-9}	***26***
Fe[N(SiMe$_3$)(Dipp)]$_2$	2	{FeN$_2$}	-	-	500	181	1×10^{-11}	***25***
Fe[N(H)Ar′]$_2$	2	{FeN$_2$}	-	-	1800	109	5×10^{-9}	***25***
Fe[N(H)Ar*]$_2$	2	{FeN$_2$}	-	-	875	104	4×10^{-8}	***25***
Fe(OAr′)$_2$	2	{FeO$_2$}	-	-	2500	43	3×10^{-7}	***25***
[Na(THF)$_6$][Co(OAr)$_3$]	3	{CoO$_3$}	−85.4	-0.11	1500	26.0	3.04×10^{-8}	***27***
[(THF)$_3$NaCo(OAr)$_3$]	3	{CoO$_3$}	−80.6	*0.15*	-	-	-	***27***
[LNiCoNiL](OTf)$_2$ (**2**)	4	{CoO$_4$}	-74.3	0	0	86.9	1.32×10^{-8}	***This work***
					2000	93.1	3.40×10^{-9}	***This work***
[^{F}L$_2$Co](TBA)$_2$	4	{CoN$_4$}	-143	0	0	286	2.25×10^{-11}	***28***
[TBA]$_2$[L$^3_2$Co]	4	{CoN$_4$}	-113	0	0	226	1.46×10^{-10}	5
(HNEt$_3$)$_2$[Co(bmsab)$_2$]	4	{CoN$_4$}	−115	0	0	230	7.63×10^{-11}	6
K$_2$[Co(bmsab)$_2$]	4	{CoN$_4$}	−100	0	0	200	3.03×10^{-9}	7
(HNEt$_3$)$_2$[Co(btsab)$_2$]	4	{CoN$_4$}	−110	0	0	220	1.1×10^{-10}	7
[K(18C6)]$_2$[Co(bmsab)$_2$]	4	{CoN$_4$}	−130	0	0	260	5.0×10^{-9}	7
[Co{(N^{t}Bu)$_3$SMe}$_2$]	4	{CoN$_4$}	- 81.3	0	0	159	6.09×10^{-10}	8-9
[Co{(N^{t}Bu)$_2$SPh}$_2$]	4	{CoN$_4$}	- 114	0	0	283	2.67×10^{-9}	9
[Co{(N^{t}Bu)$_3$SPh}$_2$]	4	{CoN$_4$}	- 75.5	0	0	213	1.76×10^{-11}	9
[Co{(N^{t}Bu)$_3$SCH$_2$PPh$_2$}$_2$]	4	{CoN$_4$}	- 79.3	0	0	199	3.08×10^{-11}	9
(HNEt$_3$)$_2$[Co(L^4)$_2$]·H$_2$O	4	{CoN$_4$}	−144.1	0.0	0	46.0	5.40×10^{-6}	10
(Bu$_4$N)$_2$[Co(L^5)$_2$]·H$_2$O	4	{CoN$_4$}	−130.8	0.005	0	58.4	2.47×10^{-6}	10
(HNEt$_3$)$_2$[CoL^6]	4	{CoN$_4$}	-128.2	0.005	0	30.5	1.13×10^{-5}	11
Co[R$_1$(C$_6$N$_2$H$_5$)R$_2$]$_2$	4	{CoN$_4$}	-91.9	0	0	183.8	1.96×10^{-10}	12
(Bu$_4$N)$_2$[Co(C$_3$S$_5$)$_2$]	4	{CoS$_4$}	-187	0	0	-	-	15

$(Ph_4P)_2[Co(C_3S_5)_2]$	4	$\{CoS_4\}$	-161	0	0	33.9	4.5×10^{-6}	15-16
$(PPN)_2[Co(C_3S_5)_2]$	4	$\{CoS_4\}$	-177	0	0	-	-	15
$[K(18C6)]_2[Co(C_3S_5)_2]$	4	$\{CoS_4\}$	-166	0	0	-	-	15
$[Co(L^8)_4](NO_3)_2$	4	$\{CoS_4\}$	-61.7	0	0	19.5	7.59×10^{-7}	20
$[Co(L^9)_4](ClO4)_2$	4	$\{CoS_4\}$	-80.7	0	0	32.0	2.24×10^{-6}	20
$C_{16}H_{52}B_{20}CoN_2S_4$	4	$\{CoS_4\}$	-71.6	0.0038	0	26.8	3.3×10^{-6}	17
$[Co(L^{10})_4](ClO4)_2$	4	$\{CoS_4\}$	-70.8	0	2000	18.7	1.55×10^{-6}	20
$(Ph_4P)_2[Co(SPh)_4]$	4	$\{CoS_4\}$	-62.0	0	0	21	1.0×10^{-6}	2, 18-19
$(Ph_4P)_2[Co(SePh)_4]$	4	$\{CoSe_4\}$	-83.0	0	0	19	3.0×10^{-6}	2
$Co[(TeP^iPr_2)_2N]_2$	4	$\{CoTe_4\}$	-45.1	0.10	0	16	2×10^{-7}	22
$K[(tpa^{Mes})Fe]$	4	$\{FeN_4\}$	−39.6	0.01	0	42	2×10^{-9}	29
$[(PMe_3)_2FeCl_3]$	5	$\{FeP_2Cl_3\}$	−50	0	0	81	1.1×10^{-10}	30
$[Co(tppm^*)][BPh_4]_2$	6	$\{CoN_6\}$	−97.2	0	0	192	2.6×10^{-12}	31
$[Co(hpy)][BPh4]_2·3CH_2Cl_2$	6	$\{CoN_6\}$	−107.5	*0.03*	0	-	-	31
$[Co(L^{11})]$ $[ZnCl_4]·CH_3OH$	6	$\{CoN_6\}$	−87.2	0	2000	24	1.6×10^{-6}	32
$[Co(L^{11})]$ $[ClO_4]·CH_3OH$	6	$\{CoN_6\}$	−116.6	0.03	1000	26.8	1.7×10^{-6}	32
$[Co(L^{11})]$ $[ClO_4]·2CH_3OH$	6	$\{CoN_6\}$	−127.6	0.001	2000	27.3	1.85×10^{-6}	32
$[Co(PzOx)_3(BC_6H_5)]Cl·CHCl_3$	6	$\{CoN_6\}$	−82	0.003	0	152	2.07×10^{-9}	33
$[Co(bpp\text{-}COOMe)_2](ClO_4)_2$	6	$\{CoN_6\}$	−57.5	0.27	1000	30.3	1.2×10^{-7}	34
$[CoTp^{py}]PF_6$	6	$\{CoN_6\}$	-156.5	0.01	0	52.8	1.56×10^{-6}	35
$[Co(tppm^*)][BPh_4]_2$	6	$\{CoN_6\}$	−97.2	*0*	0	192	2.6×10^{-12}	31
$[Co(hpy)][BPh_4]_2·3CH_2Cl_2$	6	$\{CoN_6\}$	−107.5	*0.033*	-	-	-	31
$[Co^{II}(Tpm)_2][ClO_4]_2$	6	$\{CoN_6\}$	-92	*0.114*	3000	30.6	2.0×10^{-7}	36
$[Co^{II}(Tpm)_2][BPh_4]_2·2MeCN$	6	$\{CoN_6\}$	-93	*0.124*	1500	42.5	1.0×10^{-7}	36
$(HNEt_3)(Co^{II}Co^{III}{}_3L^{11}{}_6)$	6	$\{CoO_6\}$	−115	0.024	0	75.8	1.7×10^{-7}	37

Naph = naphthyl; IPr = 1,3-bis(2,6-diisopropylphenyl)imidazol-2-ylidene); dmp = 2,6-dimesitylphenyl; cyIPr = 1,3-bis(2,6-diisopropylphenyl)-tetrahydro-benzoimidazol-2-ylidene); sIPr = 1,3-bis(2,6-diisoproplphenyl)-4,5-dihydro-imidazol-2-ylidene; Dipp = C_6H_3-2,6-$Pr^i{}_2$; Ar′ = C_6H_3-2,6-$(C_6H_3\text{-}2,6\text{-}Pr^i{}_2)_2$; Ar* = C_6H_3-2,6-$(C_6H_2\text{-}2,4,6\text{-}Pr^i{}_2)_2$; OAr^- = 2,6-di-*tert*-butylphenoxo; $H_2{}^FL$ = *N,N′*-bis(perfluorophenyl)oxalanilide; H_2L^3 = N,N'-bis(4-chlorophenyl)oxanilide; bmsab = 1,2-bis(methanesulfonamido)benzene; btsab = 1,2-bis(toluenesulfonamido)benzene; H_2L^4 = N,N′-bis(p-toluenesulfony1)oxamide; H_2L^5 = N,N′-diphenyloxamide; H_2L^6 = N,N′-bis(methanesulfonyl)oxamide; R_1 = H, R_2 = 5-(dimethylamino)naphthalen-1-ylsulfonyl; $C_3S_5{}^{2-}$ = 4,5-dimercapto-1,3-dithiole-2-thionate; L^8 = thiourea, L^9 = 1,3-dibutylthiourea, L^{10} = 1,3-phenylethylthiourea, tpa^{Mes} = tris((5-mesityl-1H-pyrrol-2-yl)methyl)amine; tppm* = 6,6′,6″-(methoxymethanetriyl)tris(2-(1H-pyrazol-1-yl)pyridine; hpy = tris(2,2′-bipyrid-6-yl)methanol; L^{11} = tris(pyridylhydrazonyl)phosphorylsulfide; bpp-COOMe=methyl 2,6-di(pyrazol-1-yl(pyridine-4-carboxylate; hpy = tris(2,2′-bipyrid-6-yl)methanol; Tp^{py} = tri(3-pyridylpyrazolyl)borate; tppm* = 6,6′,6″-(methoxymethanetriyl)tris(2-(1H-pyrazol-1-yl)pyridine; Tpm = tris(pyrazol-1-yl)methane; H_2L^{11} = *R*-4-bromo-2-((2-hydroxy-1-phenylethylimino)methyl)phenol

6. Theoretical Calculations and Analysis

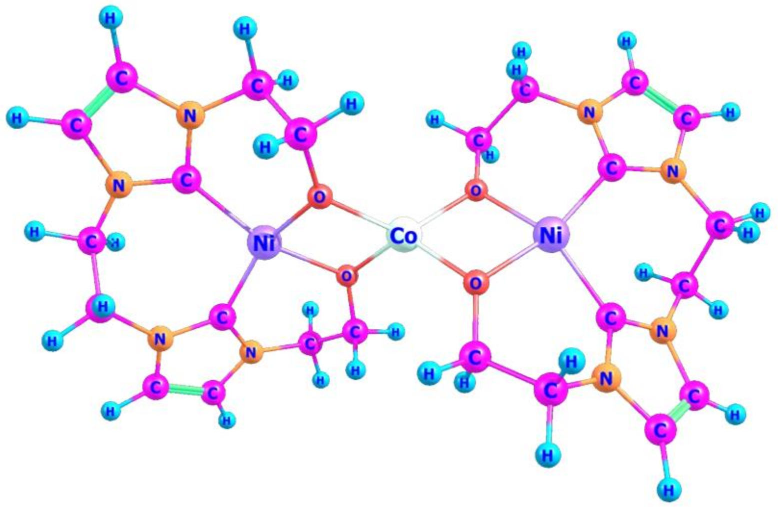

Figure S21. The truncated model complex used in the analysis of the distributions of local spins and relative energetics based on spin-unrestricted DFT geometry optimizations.

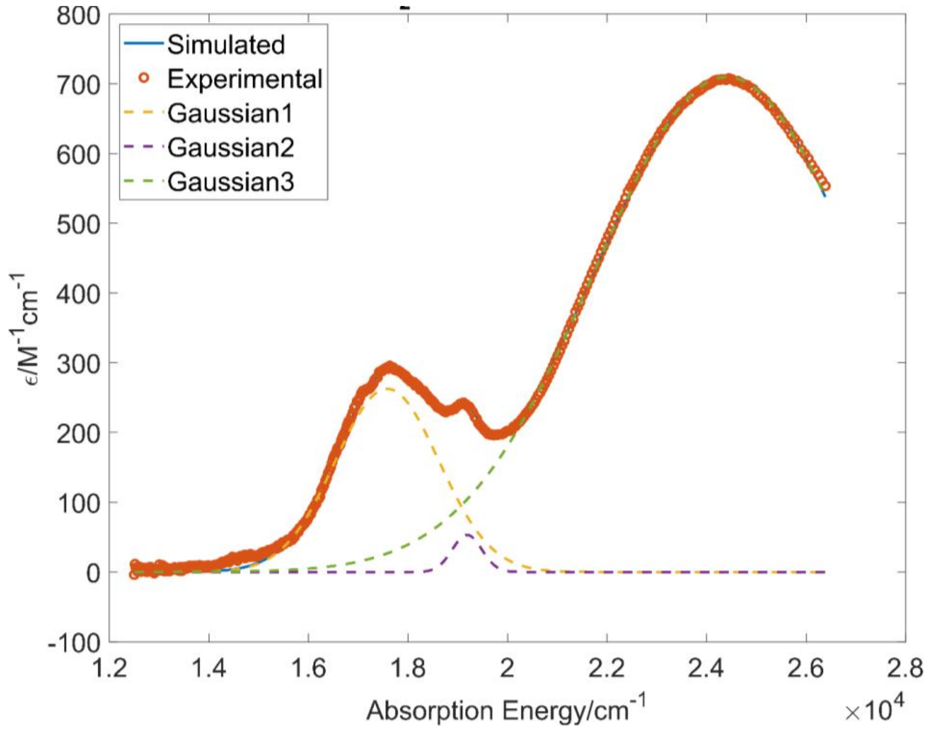

Figure S22. Deconvolution of the UV-VIS absorption spectrum of **2** into overlapping d-d transitions with in the form of three Gauss functions, $g(x) = I_o \exp[-\frac{(x-\mu)^2}{2\sigma^2}]$ with $(\mu, \sigma, I_o) =$ (17582,1041,262), (19187,297,53) and (24404,2662,710) from in the order of increasing energies in cm^{-1} and half-width-at-half-maximum , $HWHM = \sigma\sqrt{2\ln 2} =$ 1226, 350 and 3134 cm^{-1}, respectively.

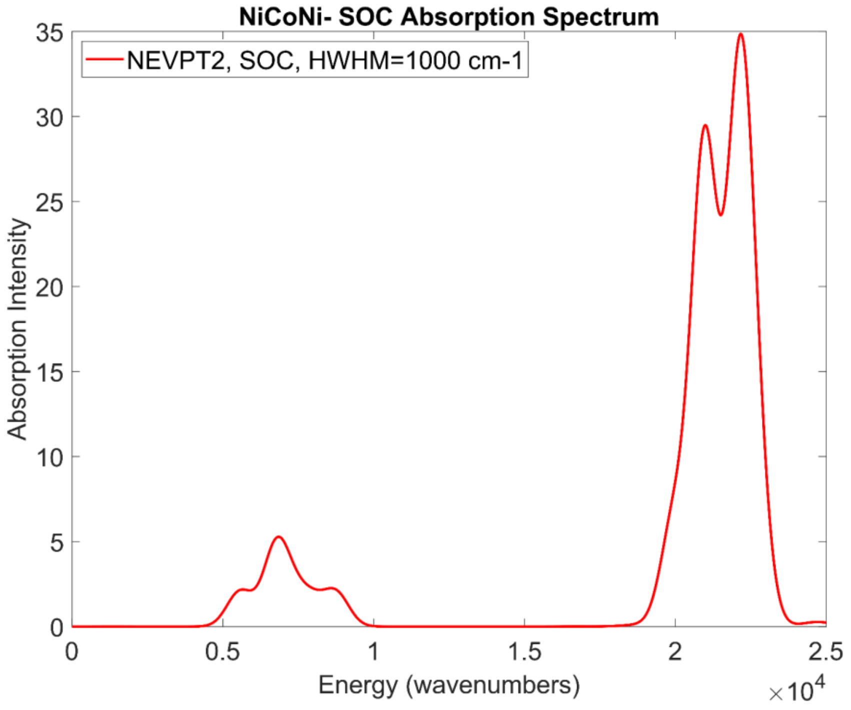

Figure S23. Spin-orbit coupling CASSCF/NEVPT2 absorption spectrum due to Co^{II} centered d-d transitions in **2**.

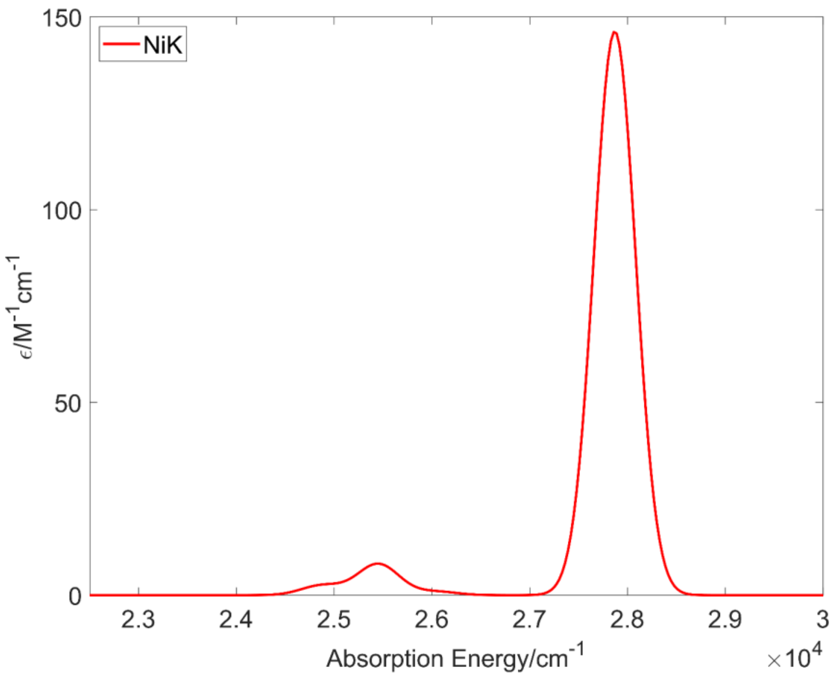

Figure S24. Spin-orbit coupling CASSCF/NEVPT2 absorption spectrum due to Ni^{II} centered d-d transitions in **1**.

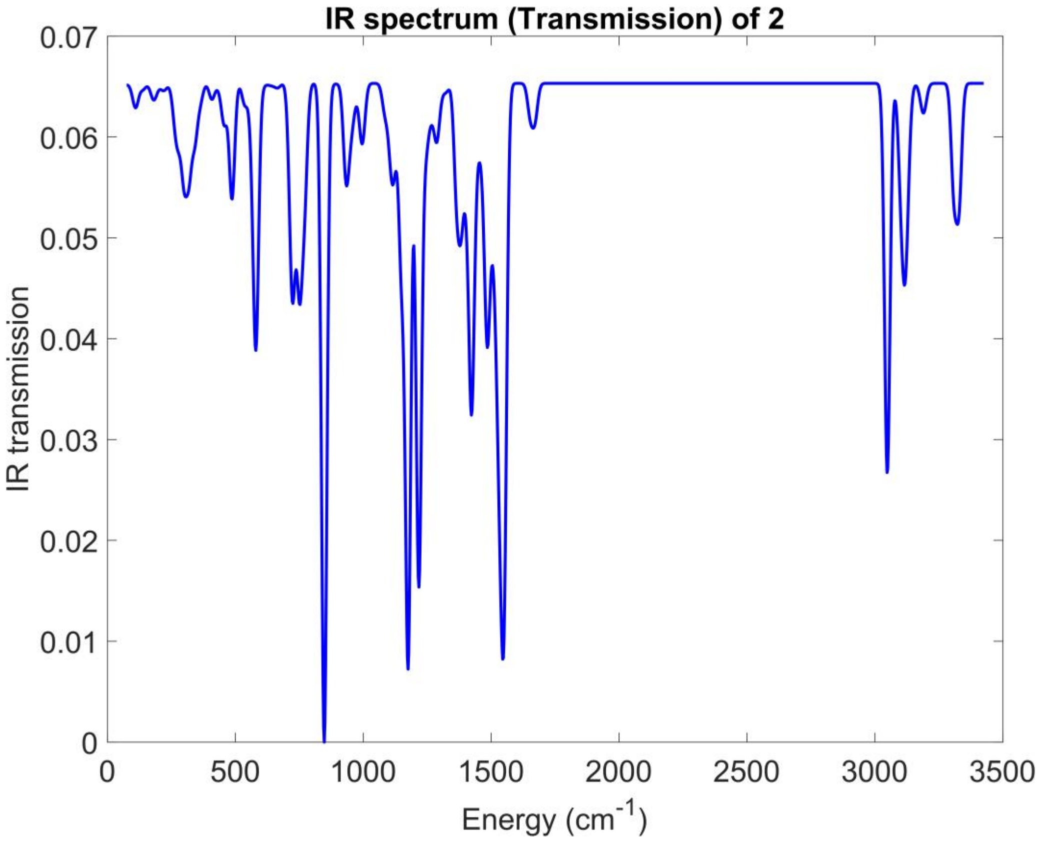

Figure S25. Computed IR Spectrum of **2** in the energy range from 0 to 3500 cm^{-1}.

Table S10. Energies of d-d transitions(spin-free) ΔE and oscillator strengths f_{osc} of the {*cis*-Ni^{II}O$_2$C$_2$} chromophore in **1**.

$\Delta E / cm^{-1}(nm)$	$f_{osc} *10^5$
24824(403)	1.4
25399(394)	3.4
25991(385)	31.1
27803(360)	61.9

Table S11. Energies of d-d transitions (spin-free) and oscillator strengths f$_{osc}$ of the {Co^{II}O$_4$} chromophore in **2**.

	$\Delta E / cm^{-1}(nm)$	$f_{osc} *10^5$
$^4A_2(^4F) \rightarrow {^4T_2}(^4F)$	960(10415)<br>4298(2327)<br>5036 (1986)	0.0<br>0.0<br>1.3
$^4A_2(^4F) \rightarrow {^4T_1}(^4F)$	5484 (1823)<br>5894 (1697)<br>6953 (1438)	0.0<br>1.1<br>0.9
$^4A_2(^4F) \rightarrow {^4T_1}(^4P)$	21223(471)<br>22845(438)<br>23877(419)	3.2<br>16.9<br>21.7

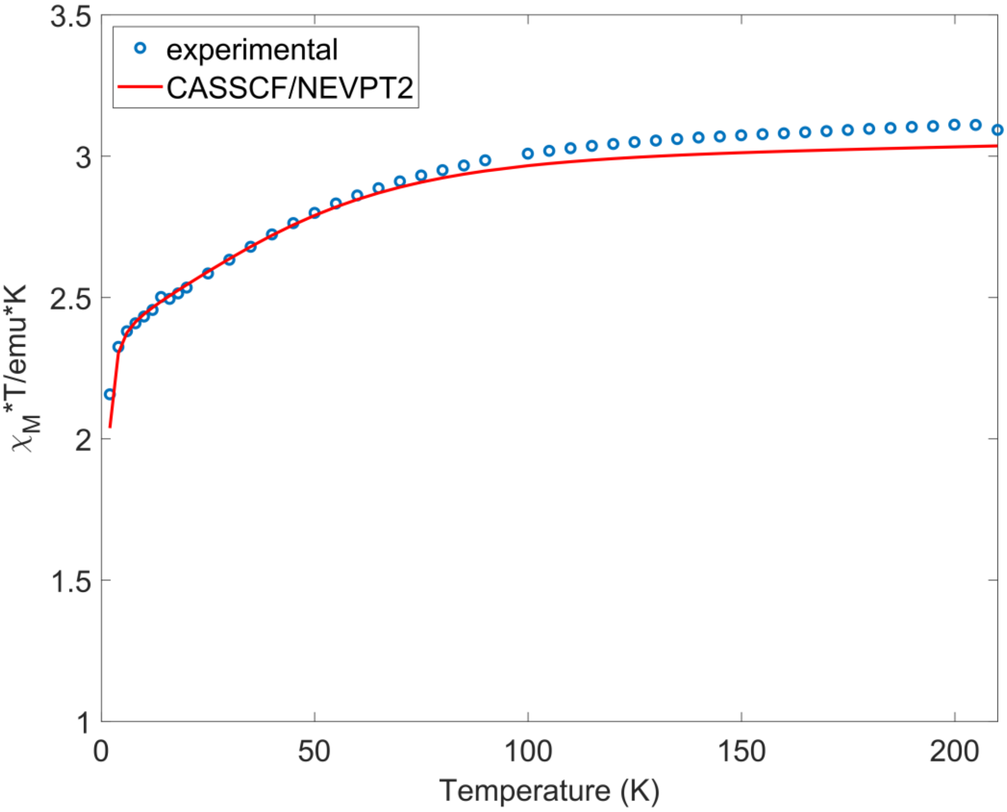

Figure S26. Comparison between experimental magnetic susceptibility data and the computed magnetic susceptibility using CASSCF/NEVPT2 ab-initio calculations.

Below we include the ORCA input files for the correlated CASSCF/NEVPT2 calculations along with the input files for the DFT geometry optimizations probing the valence/local spin distributions and their relative energies included in Table 1 of the main text.

Correlated CASSCF/NEVPT2 calculation of the entire complex **2** without truncation.

```
!DKH DKH-def2-TZVP AutoAux  NoFrozenCore PAL8

%rel method DKH
picturechange 2
end

%scf
MaxCore 26000
end

%casscf
nel 7
norb 5
mult 4,2
nroots 10,40
trafostep ri
actorbs dorbs
orbstep superci
                           switchstep diis
                          shiftup 1
                          shiftdn 1
gtol 1e-6
etol 1e-11
PTMethod DLPNO_NEVPT2
ci
nguessmat 4000
maxiter 500
end
rel
printlevel 3
dosoc true
gtensor true
end
end

*xyz 2 4
 Co     0.000000    0.000000    0.000000
  O      1.782485    0.178403    0.870889
  O      1.037494   -1.353147   -1.023763
  O     -1.068448    1.413586   -0.918456
  O     -1.735401   -0.177718    0.969190
  Ni     2.786171   -1.026691   -0.293272
  Ni    -2.775391    1.073202   -0.099741
```

N	4.670573	0.492738	1.140041
N	5.315393	-1.552858	1.047402
N	4.946145	-2.513180	-1.818359
N	3.235329	-1.845624	-2.925004
N	-3.376399	1.864668	-2.695422
N	-5.032684	2.520006	-1.505031
N	-5.218266	1.611880	1.383250
N	-4.591506	-0.433960	1.445596
C	4.351869	-0.711623	0.620482
C	5.823578	0.395203	1.914517
H	6.235013	1.095700	2.407013
C	6.241182	-0.871878	1.833010
H	7.020607	-1.237758	2.234402
C	5.438407	-2.916236	0.602089
H	4.557260	-3.364873	0.651000
H	6.067482	-3.404705	1.190103
C	5.948274	-2.941421	-0.828057
H	6.738739	-2.349276	-0.896288
H	6.241469	-3.861325	-1.046955
C	3.737069	-1.913916	-1.654002
C	5.189793	-2.788693	-3.159833
H	5.972044	-3.189354	-3.520440
C	4.109653	-2.381272	-3.850754
H	3.974991	-2.448554	-4.788755
C	3.737398	1.608166	1.107605
H	3.597902	1.903717	0.173122
H	4.104351	2.369575	1.623069
C	2.386660	1.153468	1.725496
C	1.473200	2.368793	1.913429
C	1.869800	3.663015	1.597734
H	2.699861	3.802238	1.157123
C	1.074223	4.756174	1.914900
H	1.361111	5.632727	1.687174
C	-0.135952	4.572232	2.562126
H	-0.676451	5.319191	2.791068
C	-0.546635	3.297396	2.869664
H	-1.380476	3.166156	3.305540
C	0.241491	2.197021	2.551633
H	-0.059110	1.322569	2.769540
C	2.752325	0.568263	3.102509
C	3.204321	1.439717	4.109829
H	3.151563	2.379789	3.983410
C	3.730214	0.923635	5.294910
H	4.031899	1.518517	5.971341
C	3.818216	-0.436289	5.498070
H	4.187786	-0.781105	6.302423
C	3.362200	-1.287266	4.517108
H	3.409071	-2.226328	4.652995
C	2.831232	-0.789641	3.324062
H	2.521587	-1.393418	2.659179
C	1.881611	-1.434064	-3.261684
H	1.662684	-1.737549	-4.178221
H	1.822842	-0.445982	-3.243355
C	0.873714	-2.022625	-2.275892
C	-0.559919	-1.859927	-2.816723
C	-1.641034	-2.070260	-1.951560

H	-1.480839	-2.234327	-1.029649
C	-2.947994	-2.041165	-2.429661
H	-3.671376	-2.187344	-1.831475
C	-3.204294	-1.800172	-3.774461
H	-4.097689	-1.776240	-4.096586
C	-2.135714	-1.594123	-4.644487
H	-2.301067	-1.439456	-5.567102
C	-0.839774	-1.613330	-4.174383
H	-0.122552	-1.457344	-4.777495
C	1.103007	-3.537916	-2.132519
C	1.245983	-4.338994	-3.254071
H	1.235864	-3.937600	-4.115044
C	1.401968	-5.699667	-3.150364
H	1.510422	-6.225589	-3.934020
C	1.401423	-6.304096	-1.909879
H	1.491622	-7.246937	-1.836209
C	1.267366	-5.519032	-0.765743
H	1.280155	-5.927497	0.091871
C	1.116052	-4.150197	-0.872745
H	1.020448	-3.622011	-0.088896
C	-3.797375	1.955018	-1.401356
C	-4.337706	2.309314	-3.585505
H	-4.273128	2.319887	-4.533231
C	-5.383307	2.724328	-2.844425
H	-6.200143	3.085564	-3.168150
C	-5.945177	3.012890	-0.449472
H	-6.764424	2.457058	-0.449824
H	-6.211505	3.941689	-0.665075
C	-5.341312	2.987818	0.928820
H	-4.448476	3.415131	0.910619
H	-5.915218	3.496700	1.554709
C	-4.295982	0.756733	0.898463
C	-6.084655	0.960648	2.244543
H	-6.815782	1.347803	2.711512
C	-5.694017	-0.321812	2.293421
H	-6.090355	-1.017308	2.805015
C	-2.043672	1.443203	-3.100718
H	-1.880374	1.719417	-4.037280
H	-1.981590	0.456148	-3.056446
C	-0.972530	2.067035	-2.186740
C	-1.168509	3.581169	-2.040328
C	-1.428469	4.376691	-3.170805
H	-1.555906	3.964399	-4.017127
C	-1.500652	5.760090	-3.061212
H	-1.670834	6.285475	-3.834216
C	-1.327524	6.376183	-1.841359
H	-1.376355	7.322234	-1.769897
C	-1.079146	5.593645	-0.709347
H	-0.956382	6.011477	0.134928
C	-1.010537	4.209546	-0.810207
H	-0.854317	3.687957	-0.031733
C	0.419597	1.894089	-2.818227
C	1.556744	2.006856	-2.012050
H	1.459085	2.102745	-1.071970
C	2.838014	1.979845	-2.576713
H	3.602499	2.034567	-2.015409

```
  C      2.997319    1.874003   -3.946688
  H      3.867216    1.866053   -4.328408
  C      1.878501    1.779694   -4.757343
  H      1.983070    1.713667   -5.699246
  C      0.619915    1.780900   -4.210402
  H     -0.134430    1.703697   -4.782678
  C     -3.693390   -1.569279    1.348830
  H     -3.628979   -1.866704    0.406767
  H     -4.044385   -2.321598    1.888190
  C     -2.288335   -1.159626    1.868613
  C     -2.515504   -0.590956    3.283500
  C     -2.676716    0.756098    3.503358
  H     -2.502282    1.367059    2.797114
```

Inputfile for the CASSCF/NEVPT2 calculation of the Ni-precursor complex 1 using the X-ray geometry.

```
!DKH DKH-def2-TZVP AutoAux  NoFrozenCore PAL16 notrah

%rel method DKH
picturechange 2
end

%scf
MaxCore 8000
end

%casscf
nel 8
norb 5
mult 3,1
nroots 10,15
trafostep ri
actorbs dorbs
orbstep superci
                           switchstep diis
                          shiftup 1
                          shiftdn 1
maxiter 400
PTMethod SC_NEVPT2
ci
nguessmat 4000
maxiter 500
end
rel
printlevel 3
dosoc true
gtensor true
end
end

*xyz 1 1
28       0.000000000      0.000000000      0.000000000
19       2.727453000     -1.913204000     -1.575187000
8        1.771283000     -0.484095000      0.370611000
```

8	0.171339000	-1.304880000	-1.348567000
7	0.853439000	1.383540000	2.336833000
7	-0.503927000	2.598289000	1.220632000
7	-2.822121000	1.105897000	-0.239150000
7	-2.547959000	-0.975642000	-0.636827000
6	0.095632000	1.386517000	1.215280000
6	0.699246000	2.556264000	3.051759000
1	1.117950000	2.778715000	3.874983000
6	-0.160395000	3.321747000	2.353391000
1	-0.470934000	4.187807000	2.590017000
6	-1.339831000	3.084594000	0.130208000
1	-0.918704000	2.846236000	-0.733453000
1	-1.395867000	4.071796000	0.178915000
6	-2.730137000	2.507300000	0.178539000
1	-3.072553000	2.584463000	1.104243000
1	-3.318348000	3.050149000	-0.404067000
6	-1.858419000	0.145268000	-0.288018000
6	-4.064420000	0.584216000	-0.564294000
1	-4.885151000	1.061462000	-0.597875000
6	-3.891250000	-0.719002000	-0.822365000
1	-4.559831000	-1.342192000	-1.081438000
6	1.766453000	0.298609000	2.663570000
1	1.253878000	-0.493099000	2.964530000
1	2.369100000	0.576070000	3.398384000
6	2.596844000	-0.059083000	1.410538000
6	-1.934313000	-2.287498000	-0.790055000
1	-2.611483000	-2.937737000	-1.104261000
1	-1.589722000	-2.598618000	0.084329000
6	-0.775779000	-2.210206000	-1.808018000
6	3.499819000	-1.277338000	1.725550000
6	2.940024000	-2.411033000	2.285548000
1	2.025038000	-2.396623000	2.540701000
6	3.676873000	-3.567466000	2.484780000
1	3.272464000	-4.321479000	2.897619000
6	4.994974000	-3.627078000	2.085761000
1	5.500579000	-4.422221000	2.206518000
6	5.564835000	-2.518405000	1.511013000
1	6.470712000	-2.550037000	1.226593000
6	4.829567000	-1.346904000	1.339992000
1	5.245556000	-0.585184000	0.953700000
6	3.438149000	1.160761000	0.993225000
6	3.413639000	1.591745000	-0.332877000
1	2.849370000	1.149985000	-0.956529000
6	4.201118000	2.657609000	-0.759266000
1	4.177298000	2.929755000	-1.669128000
6	5.018759000	3.322112000	0.138603000
1	5.553222000	4.052638000	-0.149773000
6	5.050562000	2.912003000	1.462020000
1	5.608472000	3.364885000	2.083416000
6	4.269513000	1.839893000	1.886404000
1	4.302729000	1.566907000	2.795729000
6	-0.105200000	-3.602897000	-1.825238000
6	0.615798000	-3.984004000	-0.683493000
1	0.642629000	-3.402801000	0.067485000
6	1.290344000	-5.195648000	-0.632835000
1	1.774321000	-5.437147000	0.148162000

```
6         1.258574000     -6.052466000     -1.719088000
1         1.720880000     -6.881798000     -1.687476000
6         0.549667000     -5.693787000     -2.850737000
1         0.526690000     -6.279775000     -3.598117000
6        -0.131918000     -4.478126000     -2.903186000
1        -0.619727000     -4.246287000     -3.684720000
6        -1.336426000     -1.792573000     -3.183596000
6        -2.457323000     -2.398504000     -3.753440000
1        -2.889874000     -3.108812000     -3.294239000
6        -2.949932000     -1.977181000     -4.983935000
1        -3.715245000     -2.398997000     -5.356604000
6        -2.328499000     -0.943681000     -5.669361000
1        -2.662729000     -0.655609000     -6.510683000
6        -1.216216000     -0.337347000     -5.113301000
1        -0.784277000      0.370495000     -5.576870000
6        -0.725014000     -0.755913000     -3.880443000
1         0.037924000     -0.327942000     -3.509957000
*
```

Input file for the calculation of the electronic energy levels of Ni^{2+} in **2** using the X-ray geometry in which Co(II) and one Ni(II) has been replaced by two diamagnetic Zn^{2+} ions. In this calculations the authors used the following truncated model complex while preserving the structure of all atoms as given by the X-ray data a optimizing the geometries of the terminal fragments only:

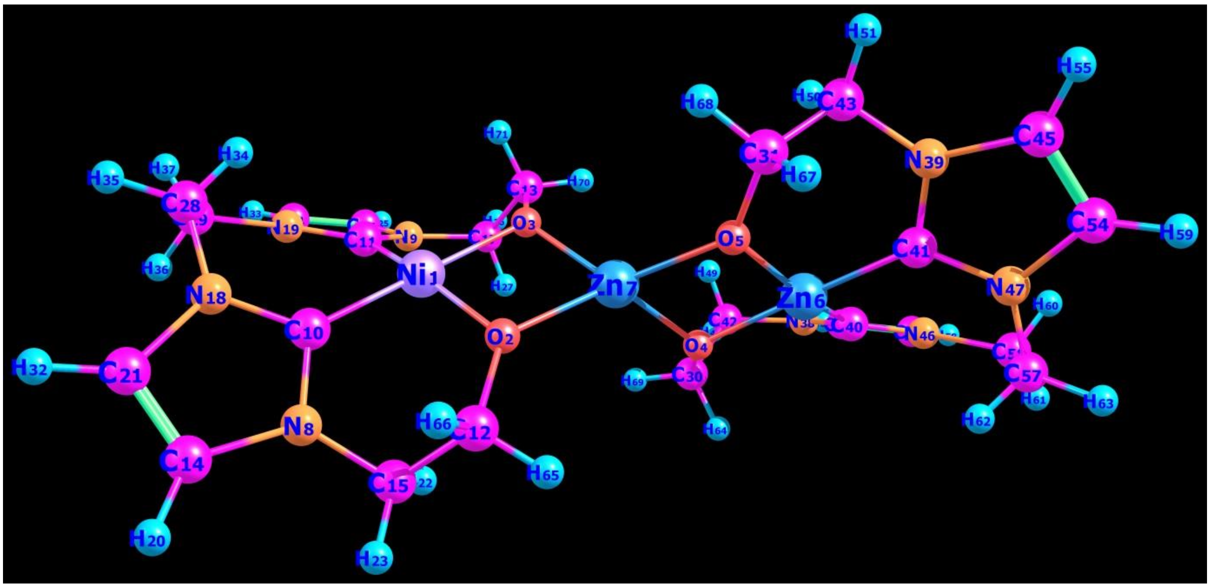

Figure S27. Truncated model complex used to probe the electronic structure of one Ni^{2+} in the presence of two closed shell Zn^{2+} ions replacing Co^{2+} and one Ni^{2+}.

```
!DKH DKH-def2-TZVP AutoAux  NoFrozenCore PAL16 notrah

%rel method DKH
picturechange 2
end

%scf
```

```
MaxCore 8000
end

%casscf
nel 8
norb 5
mult 3,1
nroots 10,15
trafostep ri
actorbs dorbs
orbstep superci
                           switchstep diis
                          shiftup 1
                          shiftdn 1
maxiter 400
PTMethod SC_NEVPT2
ci
nguessmat 4000
maxiter 500
end
rel
printlevel 3
dosoc true
gtensor true
end
end

*xyz 2 1
28       0.000000000      0.000000000      0.000000000
8       -1.003680000      1.204680000      1.163480000
8       -1.749110000     -0.326580000     -0.730380000
8       -3.854820000      2.439740000     -0.625290000
8       -4.521600000      0.848830000      1.262710000
30      -5.561320000      2.100240000      0.192610000
30      -2.786410000      1.027250000      0.292530000
7        1.883840000      1.518880000      1.432910000
7        0.448790000     -0.819770000     -2.632040000
6        1.565450000      0.314760000      0.913570000
6        0.950830000     -0.887380000     -1.361010000
6       -0.400000000      2.180660000      2.018470000
6       -1.912490000     -0.996200000     -1.982050000
6        3.037550000      1.422000000      2.207860000
6        0.950450000      2.635070000      1.401500000
6        1.323360000     -1.354160000     -3.557660000
6       -0.904820000     -0.407560000     -2.968030000
7        2.528980000     -0.525890000      1.340980000
7        2.159560000     -1.486400000     -1.525180000
1        3.448920000      2.121560000      2.699700000
6        3.454600000      0.154790000      2.125690000
1        0.811540000      2.929950000      0.466120000
1        1.317930000      3.396280000      1.916780000
6        2.403380000     -1.762270000     -2.866670000
1        1.188750000     -1.422210000     -4.495000000
1       -1.123740000     -0.711270000     -3.885840000
1       -0.963550000      0.580360000     -2.950630000
6        2.651850000     -1.889540000      0.895660000
```

6	3.162430000	-1.915390000	-0.535470000
6	-3.758490000	3.093570000	-1.894530000
6	-5.074350000	-0.133010000	2.160660000
1	4.234720000	-0.210570000	2.527720000
1	3.186130000	-2.162430000	-3.228040000
1	1.770960000	-2.338420000	0.944300000
1	3.281210000	-2.378190000	1.483170000
1	3.952990000	-1.322220000	-0.603980000
1	3.455580000	-2.834590000	-0.754320000
7	-6.162850000	2.891280000	-2.402330000
7	-7.377600000	0.592410000	1.738770000
6	-6.583990000	2.981770000	-1.107870000
6	-7.082310000	1.783610000	1.192090000
6	-4.829730000	2.469690000	-2.808440000
6	-6.479460000	-0.543110000	1.641140000
6	-7.123260000	3.336300000	-3.292810000
6	-8.479740000	0.705320000	2.585950000
7	-7.818200000	3.547100000	-1.213480000
7	-8.003900000	2.639280000	1.676120000
1	-4.666890000	2.746670000	-3.743820000
1	-4.767960000	1.482880000	-2.763700000
1	-6.415200000	-0.839860000	0.699900000
1	-6.830460000	-1.294260000	2.179840000
1	-7.059560000	3.346800000	-4.239910000
6	-8.168720000	3.750500000	-2.553010000
6	-8.869940000	1.987560000	2.536970000
1	-8.876280000	0.008860000	3.097320000
6	-8.730600000	4.039840000	-0.157380000
6	-8.126690000	4.013990000	1.221030000
1	-8.985900000	4.112980000	-2.873380000
1	-9.601480000	2.374910000	3.005380000
1	-9.550630000	3.484570000	-0.155600000
1	-8.998100000	4.969100000	-0.370370000
1	-7.234990000	4.442110000	1.205240000
1	-8.701340000	4.523640000	1.849550000
1	-3.913299523	4.154862018	-1.767670772
1	-1.114858653	2.983317705	2.123910575
1	-0.166051938	1.793797037	2.999288715
1	-5.142859611	0.293497976	3.150507150
1	-4.444146681	-1.008949755	2.205082100
1	-2.782088994	2.963531793	-2.337396955
1	-2.922870702	-0.853226486	-2.335729815
1	-1.738655577	-2.057011839	-1.877839462

*

I

C	-3.090229	1.243150	4.739333
H	-3.210679	2.176738	4.867383
C	-3.327624	0.356089	5.790669
H	-3.608982	0.682788	6.637215
C	-3.153232	-0.982364	5.595379

```
  H     -3.305556   -1.584609    6.314131
  C     -2.752070   -1.481536    4.347792
  H     -2.640446   -2.416460    4.221405
  C     -1.393347   -2.400951    1.937708
  C     -1.821825   -3.663471    1.559667
  H     -2.671102   -3.763645    1.145927
  C     -1.030899   -4.785367    1.775238
  H     -1.336241   -5.639716    1.493483
  C      0.215155   -4.663722    2.406181
  H      0.748186   -5.430523    2.580519
  C      0.647927   -3.411124    2.766501
  H      1.499273   -3.313817    3.176690
  C     -0.129611   -2.286849    2.544948
  H      0.192906   -1.431963    2.805047
*
```

Input file for the calculation of the electronic energy levels of Co^{2+} in **2** using the X-ray geometry in which the two Ni(II) ions has been replaced by two diamagnetic Zn^{2+} ions. In this calculations the authors used the following truncated model complex while preserving the structure of all atoms as given by the X-ray data a optimizing the geometries of the terminal fragments only:

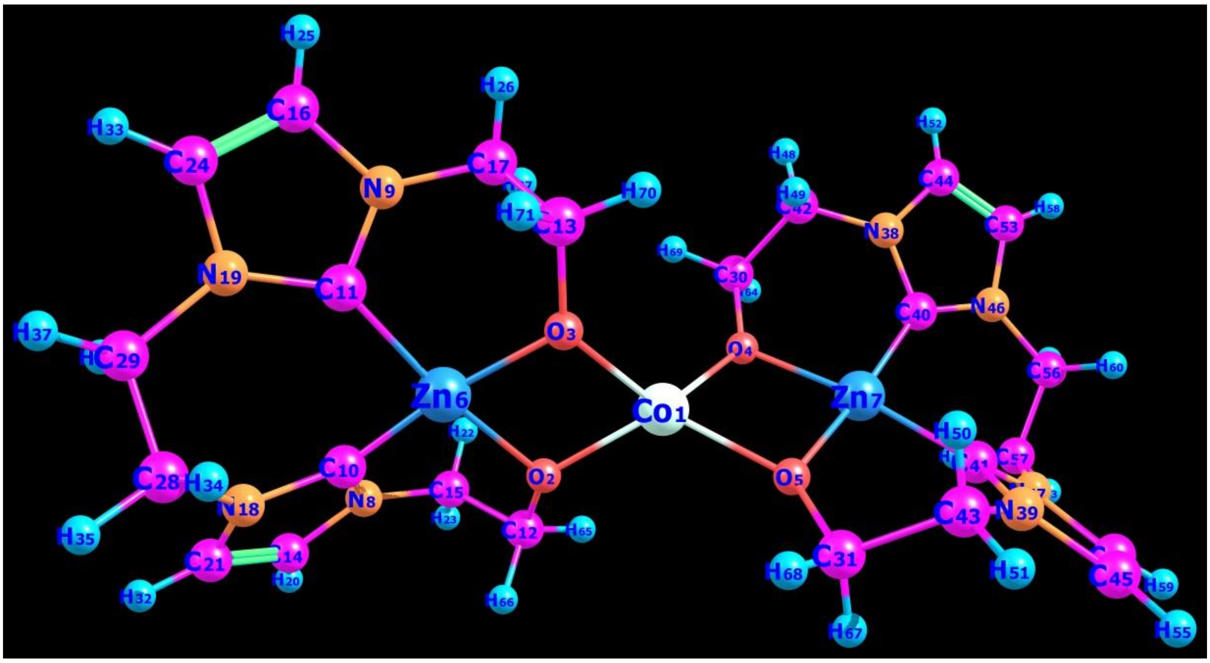

Figure S28. Truncated model complex used to probe the electronic structure of Co^{2+} in the presence of two closed shell Zn^{2+} ions replacing Ni^{2+}.

Preparation of initial guess of orbitals

cozn2nevlft2tscguess.inp

```
!DKH DKH-def2-TZVP AutoAux  NoFrozenCore PAL8 NoIter
%maxcore 3000

%rel method DKH
```

```
picturechange 2
end

#%scf
#MaxCore 26000
#end

%casscf
nel 7
norb 5
mult 4,2
nroots 10,40
trafostep ri
maxiter 1
end

*xyz 2 4
27        0.000000000       0.000000000       0.000000000
8         1.782730000       0.177430000       0.870950000
8         1.037300000      -1.353830000      -1.022910000
8        -1.068410000       1.412490000      -0.917820000
8        -1.735190000      -0.178420000       0.970180000
30        2.786410000      -1.027250000      -0.292530000
30       -2.774910000       1.072990000      -0.099920000
7         4.670250000       0.491630000       1.140380000
7         3.235200000      -1.847020000      -2.924570000
6         4.351860000      -0.712490000       0.621040000
6         3.737240000      -1.914630000      -1.653540000
6         2.386410000       1.153410000       1.725940000
6         0.873920000      -2.023450000      -2.274580000
6         5.823960000       0.394750000       1.915330000
6         3.736860000       1.607820000       1.108970000
6         4.109770000      -2.381410000      -3.850190000
6         1.881590000      -1.434810000      -3.260560000
7         5.315390000      -1.553140000       1.048450000
7         4.945970000      -2.513650000      -1.817710000
1         6.235330000       1.094310000       2.407170000
6         6.241010000      -0.872460000       1.833160000
1         3.597950000       1.902700000       0.173590000
1         4.104340000       2.369030000       1.624250000
6         5.189790000      -2.789520000      -3.159200000
1         3.975160000      -2.449460000      -4.787530000
1         1.662670000      -1.738520000      -4.178370000
1         1.822860000      -0.446890000      -3.243160000
6         5.438260000      -2.916790000       0.603130000
6         5.948840000      -2.942640000      -0.828000000
6        -0.972080000       2.066320000      -2.187060000
6        -2.287940000      -1.160260000       1.868130000
1         7.021130000      -1.237820000       2.235190000
1         5.972540000      -3.189680000      -3.520570000
1         4.557370000      -3.365670000       0.651770000
1         6.067620000      -3.405440000       1.190640000
1         6.739400000      -2.349470000      -0.896510000
1         6.241990000      -3.861840000      -1.046850000
7        -3.376440000       1.864030000      -2.694860000
7        -4.591190000      -0.434840000       1.446240000
```

```
6       -3.797580000      1.954520000     -1.400400000
6       -4.295900000      0.756360000      0.899560000
6       -2.043320000      1.442440000     -3.100970000
6       -3.693050000     -1.570360000      1.348610000
6       -4.336850000      2.309050000     -3.585340000
6       -5.693330000     -0.321930000      2.293420000
7       -5.031790000      2.519850000     -1.506010000
7       -5.217490000      1.612030000      1.383590000
1       -1.880480000      1.719420000     -4.036350000
1       -1.981550000      0.455630000     -3.056230000
1       -3.628790000     -1.867110000      0.407370000
1       -4.044050000     -2.321510000      1.887310000
1       -4.273150000      2.319550000     -4.532440000
6       -5.382310000      2.723250000     -2.845540000
6       -6.083530000      0.960310000      2.244440000
1       -6.089870000     -1.018390000      2.804790000
6       -5.944190000      3.012590000     -0.449910000
6       -5.340280000      2.986740000      0.928500000
1       -6.199490000      3.085730000     -3.165910000
1       -6.815070000      1.347660000      2.712850000
1       -6.764220000      2.457320000     -0.448130000
1       -6.211690000      3.941850000     -0.662900000
1       -4.448580000      3.414860000      0.912710000
1       -5.914930000      3.496390000      1.557020000
1       -1.126889523      3.127612018     -2.060200772
1        1.671551347      1.956067705      1.831380575
1        2.620358062      0.766547037      2.706758715
1       -2.356449611     -0.733752024      2.857977150
1       -1.657736681     -2.036199755      1.912552100
1        0.004321006      1.936281793     -2.629926955
1       -0.136460702     -1.880476486     -2.628259815
1        1.047754423     -3.084261839     -2.170369462
*
```

Reading the initial guess of orbitals (cozn2nevlft2tscguess.gbw)

rotating orbitals of Co^{2+} outside cas- into the cas space

cozn2nevlft2t.inp:

```
!DKH DKH-def2-TZVP AutoAux  NoFrozenCore PAL16 moread
%moinp "cozn2nevlft2tscguess.gbw"
%maxcore 10000

%rel method DKH
picturechange 2
end

%scf rotate {171,174,90} {172,175,90} end end

%casscf
nel 7
norb 5
mult 4,2
```

```
nroots 10,40
trafostep ri
actorbs dorbs
orbstep superci
                           switchstep diis
                          shiftup 1
                          shiftdn 1
#maxiter 100
##gtol 1e-6
##etol 1e-11
PTMethod SC_NEVPT2
ci
nguessmat 4000
maxiter 500
end
rel
printlevel 3
dosoc true
gtensor true
end
end

*xyz 2 4
27       0.000000000      0.000000000      0.000000000
8        1.782730000      0.177430000      0.870950000
8        1.037300000     -1.353830000     -1.022910000
8       -1.068410000      1.412490000     -0.917820000
8       -1.735190000     -0.178420000      0.970180000
30       2.786410000     -1.027250000     -0.292530000
30      -2.774910000      1.072990000     -0.099920000
7        4.670250000      0.491630000      1.140380000
7        3.235200000     -1.847020000     -2.924570000
6        4.351860000     -0.712490000      0.621040000
6        3.737240000     -1.914630000     -1.653540000
6        2.386410000      1.153410000      1.725940000
6        0.873920000     -2.023450000     -2.274580000
6        5.823960000      0.394750000      1.915330000
6        3.736860000      1.607820000      1.108970000
6        4.109770000     -2.381410000     -3.850190000
6        1.881590000     -1.434810000     -3.260560000
7        5.315390000     -1.553140000      1.048450000
7        4.945970000     -2.513650000     -1.817710000
1        6.235330000      1.094310000      2.407170000
6        6.241010000     -0.872460000      1.833160000
1        3.597950000      1.902700000      0.173590000
1        4.104340000      2.369030000      1.624250000
6        5.189790000     -2.789520000     -3.159200000
1        3.975160000     -2.449460000     -4.787530000
1        1.662670000     -1.738520000     -4.178370000
1        1.822860000     -0.446890000     -3.243160000
6        5.438260000     -2.916790000      0.603130000
6        5.948840000     -2.942640000     -0.828000000
6       -0.972080000      2.066320000     -2.187060000
6       -2.287940000     -1.160260000      1.868130000
1        7.021130000     -1.237820000      2.235190000
1        5.972540000     -3.189680000     -3.520570000
```

```
1        4.557370000     -3.365670000      0.651770000
1        6.067620000     -3.405440000      1.190640000
1        6.739400000     -2.349470000     -0.896510000
1        6.241990000     -3.861840000     -1.046850000
7       -3.376440000      1.864030000     -2.694860000
7       -4.591190000     -0.434840000      1.446240000
6       -3.797580000      1.954520000     -1.400400000
6       -4.295900000      0.756360000      0.899560000
6       -2.043320000      1.442440000     -3.100970000
6       -3.693050000     -1.570360000      1.348610000
6       -4.336850000      2.309050000     -3.585340000
6       -5.693330000     -0.321930000      2.293420000
7       -5.031790000      2.519850000     -1.506010000
7       -5.217490000      1.612030000      1.383590000
1       -1.880480000      1.719420000     -4.036350000
1       -1.981550000      0.455630000     -3.056230000
1       -3.628790000     -1.867110000      0.407370000
1       -4.044050000     -2.321510000      1.887310000
1       -4.273150000      2.319550000     -4.532440000
6       -5.382310000      2.723250000     -2.845540000
6       -6.083530000      0.960310000      2.244440000
1       -6.089870000     -1.018390000      2.804790000
6       -5.944190000      3.012590000     -0.449910000
6       -5.340280000      2.986740000      0.928500000
1       -6.199490000      3.085730000     -3.165910000
1       -6.815070000      1.347660000      2.712850000
1       -6.764220000      2.457320000     -0.448130000
1       -6.211690000      3.941850000     -0.662900000
1       -4.448580000      3.414860000      0.912710000
1       -5.914930000      3.496390000      1.557020000
1       -1.126889523      3.127612018     -2.060200772
1        1.671551347      1.956067705      1.831380575
1        2.620358062      0.766547037      2.706758715
1       -2.356449611     -0.733752024      2.857977150
1       -1.657736681     -2.036199755      1.912552100
1        0.004321006      1.936281793     -2.629926955
1       -0.136460702     -1.880476486     -2.628259815
1        1.047754423     -3.084261839     -2.170369462
*
```

DFT optimization of the entire complex without truncation

```
!UKS wB97X-D4 D3BJ DKH2 DKH-def2-SVP opt  Autoaux

%basis
newgto Co "dkh-def2-tzvp" end
newgto Ni "dkh-def2-tzvp" end
end

%pal nprocs 16 end

%maxcore 8000
```

```
%rel method DKH
picturechange 2
end

%scf maxiter 500 shift shift 0.5 erroff 0 end end

*xyz 2 4
 Co     0.000000    0.000000    0.000000
  O      1.782485    0.178403    0.870889
  O      1.037494   -1.353147   -1.023763
  O     -1.068448    1.413586   -0.918456
  O     -1.735401   -0.177718    0.969190
  Ni     2.786171   -1.026691   -0.293272
  Ni    -2.775391    1.073202   -0.099741
  N      4.670573    0.492738    1.140041
  N      5.315393   -1.552858    1.047402
  N      4.946145   -2.513180   -1.818359
  N      3.235329   -1.845624   -2.925004
  N     -3.376399    1.864668   -2.695422
  N     -5.032684    2.520006   -1.505031
  N     -5.218266    1.611880    1.383250
  N     -4.591506   -0.433960    1.445596
  C      4.351869   -0.711623    0.620482
  C      5.823578    0.395203    1.914517
  H      6.235013    1.095700    2.407013
  C      6.241182   -0.871878    1.833010
  H      7.020607   -1.237758    2.234402
  C      5.438407   -2.916236    0.602089
  H      4.557260   -3.364873    0.651000
  H      6.067482   -3.404705    1.190103
  C      5.948274   -2.941421   -0.828057
  H      6.738739   -2.349276   -0.896288
  H      6.241469   -3.861325   -1.046955
  C      3.737069   -1.913916   -1.654002
  C      5.189793   -2.788693   -3.159833
  H      5.972044   -3.189354   -3.520440
  C      4.109653   -2.381272   -3.850754
  H      3.974991   -2.448554   -4.788755
  C      3.737398    1.608166    1.107605
  H      3.597902    1.903717    0.173122
  H      4.104351    2.369575    1.623069
  C      2.386660    1.153468    1.725496
  C      1.473200    2.368793    1.913429
  C      1.869800    3.663015    1.597734
  H      2.699861    3.802238    1.157123
  C      1.074223    4.756174    1.914900
  H      1.361111    5.632727    1.687174
  C     -0.135952    4.572232    2.562126
  H     -0.676451    5.319191    2.791068
  C     -0.546635    3.297396    2.869664
  H     -1.380476    3.166156    3.305540
  C      0.241491    2.197021    2.551633
  H     -0.059110    1.322569    2.769540
  C      2.752325    0.568263    3.102509
  C      3.204321    1.439717    4.109829
```

H	3.151563	2.379789	3.983410
C	3.730214	0.923635	5.294910
H	4.031899	1.518517	5.971341
C	3.818216	-0.436289	5.498070
H	4.187786	-0.781105	6.302423
C	3.362200	-1.287266	4.517108
H	3.409071	-2.226328	4.652995
C	2.831232	-0.789641	3.324062
H	2.521587	-1.393418	2.659179
C	1.881611	-1.434064	-3.261684
H	1.662684	-1.737549	-4.178221
H	1.822842	-0.445982	-3.243355
C	0.873714	-2.022625	-2.275892
C	-0.559919	-1.859927	-2.816723
C	-1.641034	-2.070260	-1.951560
H	-1.480839	-2.234327	-1.029649
C	-2.947994	-2.041165	-2.429661
H	-3.671376	-2.187344	-1.831475
C	-3.204294	-1.800172	-3.774461
H	-4.097689	-1.776240	-4.096586
C	-2.135714	-1.594123	-4.644487
H	-2.301067	-1.439456	-5.567102
C	-0.839774	-1.613330	-4.174383
H	-0.122552	-1.457344	-4.777495
C	1.103007	-3.537916	-2.132519
C	1.245983	-4.338994	-3.254071
H	1.235864	-3.937600	-4.115044
C	1.401968	-5.699667	-3.150364
H	1.510422	-6.225589	-3.934020
C	1.401423	-6.304096	-1.909879
H	1.491622	-7.246937	-1.836209
C	1.267366	-5.519032	-0.765743
H	1.280155	-5.927497	0.091871
C	1.116052	-4.150197	-0.872745
H	1.020448	-3.622011	-0.088896
C	-3.797375	1.955018	-1.401356
C	-4.337706	2.309314	-3.585505
H	-4.273128	2.319887	-4.533231
C	-5.383307	2.724328	-2.844425
H	-6.200143	3.085564	-3.168150
C	-5.945177	3.012890	-0.449472
H	-6.764424	2.457058	-0.449824
H	-6.211505	3.941689	-0.665075
C	-5.341312	2.987818	0.928820
H	-4.448476	3.415131	0.910619
H	-5.915218	3.496700	1.554709
C	-4.295982	0.756733	0.898463
C	-6.084655	0.960648	2.244543
H	-6.815782	1.347803	2.711512
C	-5.694017	-0.321812	2.293421
H	-6.090355	-1.017308	2.805015
C	-2.043672	1.443203	-3.100718
H	-1.880374	1.719417	-4.037280
H	-1.981590	0.456148	-3.056446
C	-0.972530	2.067035	-2.186740
C	-1.168509	3.581169	-2.040328

```
  C     -1.428469    4.376691   -3.170805
  H     -1.555906    3.964399   -4.017127
  C     -1.500652    5.760090   -3.061212
  H     -1.670834    6.285475   -3.834216
  C     -1.327524    6.376183   -1.841359
  H     -1.376355    7.322234   -1.769897
  C     -1.079146    5.593645   -0.709347
  H     -0.956382    6.011477    0.134928
  C     -1.010537    4.209546   -0.810207
 H     -0.854317    3.687957   -0.031733
  C      0.419597    1.894089   -2.818227
  C      1.556744    2.006856   -2.012050
  H      1.459085    2.102745   -1.071970
  C      2.838014    1.979845   -2.576713
  H      3.602499    2.034567   -2.015409
  C      2.997319    1.874003   -3.946688
  H      3.867216    1.866053   -4.328408
  C      1.878501    1.779694   -4.757343
  H      1.983070    1.713667   -5.699246
  C      0.619915    1.780900   -4.210402
  H     -0.134430    1.703697   -4.782678
  C     -3.693390   -1.569279    1.348830
  H     -3.628979   -1.866704    0.406767
  H     -4.044385   -2.321598    1.888190
  C     -2.288335   -1.159626    1.868613
  C     -2.515504   -0.590956    3.283500
  C     -2.676716    0.756098    3.503358
  H     -2.502282    1.367059    2.797114
  C     -3.090229    1.243150    4.739333
  H     -3.210679    2.176738    4.867383
  C     -3.327624    0.356089    5.790669
  H     -3.608982    0.682788    6.637215
  C     -3.153232   -0.982364    5.595379
  H     -3.305556   -1.584609    6.314131
  C     -2.752070   -1.481536    4.347792
  H     -2.640446   -2.416460    4.221405
  C     -1.393347   -2.400951    1.937708
  C     -1.821825   -3.663471    1.559667
  H     -2.671102   -3.763645    1.145927
  C     -1.030899   -4.785367    1.775238
  H     -1.336241   -5.639716    1.493483
  C      0.215155   -4.663722    2.406181
  H      0.748186   -5.430523    2.580519
  C      0.647927   -3.411124    2.766501
  H      1.499273   -3.313817    3.176690
  C     -0.129611   -2.286849    2.544948
  H      0.192906   -1.431963    2.805047
*
```

xyz file of the optimized geometry:

151

Coordinates from ORCA-job coni2nevlft2opt

```
  Co  0.01567280752068      0.00611071929467     -0.07743058655135
```

O	1.78548287997566	0.27945602476186	0.77839494516315
O	1.04716986161428	-1.34733119691646	-1.09363239145836
O	-1.06076504145759	1.39473348814606	-0.99936944077393
O	-1.69557441818677	-0.26952056133573	0.88802166517803
Ni	2.83405016743709	-0.96633338149734	-0.35208143891904
Ni	-2.80115302920703	1.00811904102153	-0.14815510719080
N	4.64105482948348	0.68238905645643	1.08244881983765
N	5.31904330184489	-1.35230435199484	1.08034451331486
N	4.84523883519819	-2.82596264276432	-1.62979753656972
N	3.22874057626418	-2.14222135998244	-2.86476193295877
N	-3.36007460340841	2.18490907454125	-2.62208671724620
N	-4.87737208422274	2.89051328910409	-1.27786157626410
N	-5.18980206560601	1.39310370249738	1.43616930390164
N	-4.53214565279255	-0.64757339276149	1.36548576413925
C	4.36820890897909	-0.53803965955370	0.58296644000213
C	5.74895815166641	0.63640792756394	1.91649107674962
H	6.11632667591648	1.50923780122105	2.44371086851025
C	6.18624650801238	-0.64949246221984	1.90939296929512
H	7.01903295424326	-1.12037562130457	2.41947671749193
C	5.32966845615316	-2.78631688726438	0.83488166298019
H	4.32371612201384	-3.18439264780399	1.01602292569742
H	6.00941563694753	-3.24832139110657	1.55837252554189
C	5.80342819534116	-3.12777557083070	-0.56312201399528
H	6.74514150020474	-2.60385308376004	-0.77399350209653
H	5.99926308014740	-4.20556778404054	-0.60710414758077
C	3.73375920140863	-2.04463749040220	-1.61266485647688
C	5.01750181770035	-3.40948530795152	-2.87961273251678
H	5.84383973610741	-4.07699510899446	-3.09641588946469
C	3.99169646958640	-2.98321679885138	-3.65332074060082
H	3.73368110644858	-3.21138347661433	-4.68076922532758
C	3.67154971155291	1.76572647033367	1.01143426855267
H	3.53842719429350	2.06645993365475	-0.03021434024696

H	4.07050387464736	2.61010753809855	1.57916484746424
C	2.32503539011717	1.28483670126642	1.61188060433860
C	1.35816823799631	2.46596418320534	1.75325994940444
C	1.62610916311466	3.74303319775921	1.24391710522298
H	2.49586620030693	3.93018474358825	0.61654542058323
C	0.80043774036913	4.82637782309889	1.55418260906090
H	1.03579354387879	5.81322089661466	1.15722251236099
C	-0.30965286103177	4.64466337111179	2.37634061462749
H	-0.93214854349104	5.49520148183250	2.65419825200694
C	-0.61507611915103	3.36554314407981	2.84649887866727
H	-1.47261840595735	3.21799938589590	3.50079498507082
C	0.20967447540215	2.28503084168400	2.53440549021226
H	-0.00369744382612	1.30395701910044	2.95667449300593
C	2.66450825647886	0.73926182999390	3.01064958482429
C	2.96686958089931	1.62710983902458	4.05252480914519
H	2.85427267121532	2.70167121278411	3.90071238157293
C	3.39399006296817	1.15064197193290	5.29205021742420
H	3.61775568064207	1.85365479749902	6.09436225061575
C	3.52794820351992	-0.22379816712220	5.50749840975972
H	3.86013425182087	-0.59736020248979	6.47584902378224
C	3.22595572757499	-1.11231288205788	4.47554800151455
H	3.32429160383483	-2.18600727198963	4.64082196158635
C	2.79729053273992	-0.63136794392737	3.23490958510054
H	2.55717357382785	-1.31869972188715	2.42831119338789
C	1.93622344954465	-1.60798445334265	-3.27768619650532
H	1.76038170704430	-1.99143786131452	-4.28374223446007
H	1.97206961298218	-0.51852336603466	-3.31094877618706
C	0.83698309401650	-2.05170911873335	-2.29375756542107
C	-0.56249182808857	-1.78952664678076	-2.86710258351532
C	-1.66475134942526	-1.98411309984688	-2.02357569256465
H	-1.48444115798260	-2.23697786186592	-0.98044038132430
C	-2.96688199681215	-1.91038679988379	-2.51986002573444

H	-3.81936883579681	-2.07887323741180	-1.86231051526644
C	-3.19510220352501	-1.66002832243585	-3.87629759046171
H	-4.21167000137955	-1.63192120594000	-4.26769388409903
C	-2.10652332288985	-1.45582846840376	-4.72252670401234
H	-2.26591294042380	-1.25997842551330	-5.78303092688037
C	-0.80293970002102	-1.50922377360466	-4.21779496466528
H	0.01806998595446	-1.33744484768892	-4.90944549551911
C	0.98504749617705	-3.57031834555020	-2.09050440735455
C	0.74356298292061	-4.44062026147882	-3.16379858068661
H	0.35074975410532	-4.05144674183326	-4.10412485358927
C	0.98149426699982	-5.80821274253041	-3.03970597458506
H	0.78413663326009	-6.47218462141590	-3.88129465510706
C	1.46960393322315	-6.32635829995947	-1.83600468335964
H	1.66080627879308	-7.39483852256279	-1.73712509877716
C	1.69170137679799	-5.46791249826188	-0.76048218349361
H	2.04926965017746	-5.86814881148587	0.18807931347091
C	1.44253896824952	-4.09658361591620	-0.88336815010279
H	1.58415278576776	-3.43137649016908	-0.03403802648221
C	-3.77535370071970	2.09915216667660	-1.33687773940287
C	-4.16908985232191	3.03014439450356	-3.35858196173899
H	-3.98381383671202	3.24998908654417	-4.40335595519648
C	-5.13223166089569	3.47101948394714	-2.51528460734222
H	-5.96557193983816	4.14565808935954	-2.67605232980842
C	-5.75373733786179	3.20333692026125	-0.14597085702755
H	-6.72045145319464	2.70599852202138	-0.30040169827236
H	-5.92417950419849	4.28627871092996	-0.16371580998999
C	-5.19761543944490	2.83128254636349	1.21370547653645
H	-4.17675925250342	3.21323358824654	1.33717272708112
H	-5.82379897480108	3.29033771485210	1.98579040227550
C	-4.27722968609146	0.57861422232666	0.87221352726110
C	-6.01490883560442	0.68326899992934	2.30171702436436
H	-6.81238205189202	1.15234650495101	2.86693624059838

C	-5.58972688632205	-0.60641552757310	2.26271861914593
H	-5.93480587470647	-1.48479549572885	2.79582186860273
C	-2.10290980743870	1.63459826796790	-3.11645481448794
H	-1.99805060725614	1.99503845538340	-4.14068921036703
H	-2.14726003180887	0.54490287426029	-3.12419817254250
C	-0.93588234287976	2.08876814941681	-2.21759616552134
C	-1.07284111321803	3.60870128113423	-2.01539150253494
C	-0.92091977033111	4.47189034838754	-3.11034801369308
H	-0.60548123795284	4.07700583688996	-4.07698413734047
C	-1.14912282664961	5.84022615845386	-2.97611268617262
H	-1.02158511655983	6.49871102488975	-3.83530004204930
C	-1.53759987270588	6.36594559667891	-1.73988237474773
H	-1.71941240854707	7.43519987985991	-1.63223948840306
C	-1.67357813776229	5.51386971346494	-0.64501765383162
H	-1.95399748903950	5.91995421188623	0.32686821446521
C	-1.43562157774345	4.14168334523564	-0.77938128032909
H	-1.51309614073200	3.48023800593148	0.08095641313398
C	0.42296439080635	1.82560777871905	-2.88155445169137
C	1.57590979933806	1.97023776881085	-2.09843759171503
H	1.46242989266281	2.18371182189675	-1.03768106657021
C	2.84594028342789	1.88833939321628	-2.67090918141807
H	3.73807419971411	2.00878587471538	-2.05690525470771
C	2.99095345483359	1.68398093444828	-4.04603930918139
H	3.98258724494319	1.64831144292923	-4.49631476013736
C	1.85133113527775	1.53255389580707	-4.83438475856656
H	1.94550917201856	1.37270096709112	-5.90862439736022
C	0.58015362116571	1.59106972509601	-4.25323073316207
H	-0.28224673420350	1.46063466963669	-4.90249814296520
C	-3.58200020571711	-1.73948914755472	1.20981034504432
H	-3.51890280462732	-2.01687521596545	0.15512819325807
H	-3.95462808167434	-2.59143073193133	1.78399371666250
C	-2.19359860278586	-1.28729301224518	1.73210520248290

C	-2.43603118910503	-0.77214256999004	3.16257116840147
C	-2.55253885476618	0.59327546938709	3.42453989546772
H	-2.36586683105337	1.29741073456983	2.61857496169785
C	-2.89799876774177	1.04747341505017	4.70068098621631
H	-2.98484488483506	2.11720425114284	4.89544142716052
C	-3.13120867308626	0.13706573158918	5.73131093912310
H	-3.39787017490744	0.48991511468831	6.72730022850899
C	-3.01327089962413	-1.23231048280316	5.47803973783258
H	-3.18447391933461	-1.95237728469299	6.27816022791226
C	-2.66946412670966	-1.68219065850818	4.20306934004689
H	-2.56876918390516	-2.75319436216367	4.02128065144345
C	-1.23225606115521	-2.47970034473840	1.78687065807768
C	-1.55494990481644	-3.75271338166423	1.30015360946913
H	-2.47326820470763	-3.92867378969629	0.74243629713191
C	-0.72139667046981	-4.84644927845209	1.54754395603084
H	-0.99840610547739	-5.83069829283311	1.17157889842565
C	0.44873842615087	-4.67944008705799	2.28497826317077
H	1.07720913208693	-5.53874873668770	2.51840022940324
C	0.80816499218845	-3.40387701408495	2.72541196471757
H	1.71517499470846	-3.26698063953979	3.31210301884953
C	-0.02320472604409	-2.31291269283146	2.47389687484664
H	0.23415280641079	-1.33390604258243	2.87576120925477

Using this geometry harmonic frequencies have been calculated using the following input file:

```
!UKS wB97X-D4 D3BJ DKH2 DKH-def2-SVP freq  Autoaux moread
%moinp "coni2nevlft2opt.gbw"

%basis
newgto Co "dkh-def2-tzvp" end
newgto Ni "dkh-def2-tzvp" end
end

%pal nprocs 8 end

%maxcore  100000

%rel method DKH
```

```
picturechange 2
end

%scf maxiter 500 shift shift 0.5 erroff 0 end end

*xyzfile 2 4 coni2nevlft2opt.xyz
```

Computed IR frequencies and intensities are:

```
-----------
IR SPECTRUM
-----------

 Mode   freq       eps      Int      T**2         TX        TY        TZ
       cm**-1   L/(mol*cm) km/mol    a.u.
----------------------------------------------------------------------------
  6:     20.98   0.000000    0.00  0.000007  ( 0.000798  0.002562  0.000273)
  7:     25.95   0.000140    0.71  0.001684  ( 0.008127  0.019650 -0.035103)
  8:     26.45   0.000142    0.72  0.001678  (-0.016324 -0.031051 -0.021157)
  9:     34.15   0.000107    0.54  0.000979  (-0.001079  0.002111 -0.031193)
 10:     34.96   0.000020    0.10  0.000182  (-0.012509 -0.000160  0.005027)
 11:     40.23   0.000043    0.22  0.000332  ( 0.000464  0.001769 -0.018125)
 12:     41.11   0.000140    0.71  0.001060  ( 0.002860  0.032393  0.001687)
 13:     44.71   0.000251    1.27  0.001753  ( 0.030864  0.028275 -0.001153)
 14:     46.80   0.000016    0.08  0.000108  (-0.009830 -0.001677 -0.002931)
 15:     48.35   0.000006    0.03  0.000039  ( 0.003119 -0.003255 -0.004375)
 16:     53.49   0.000170    0.86  0.000994  (-0.004413 -0.003200 -0.031053)
 17:     57.22   0.000025    0.12  0.000135  ( 0.000880  0.002014  0.011394)
 18:     57.90   0.000038    0.19  0.000203  (-0.007446 -0.010124  0.006681)
 19:     61.50   0.000022    0.11  0.000113  (-0.000182  0.000368  0.010624)
 20:     64.87   0.000210    1.06  0.001010  (-0.012136 -0.006463 -0.028651)
 21:     66.64   0.000517    2.61  0.002419  ( 0.037570  0.030636 -0.008329)
 22:     68.91   0.000033    0.17  0.000149  (-0.009706  0.007265  0.001410)
 23:     70.88   0.000026    0.13  0.000115  (-0.001044 -0.010678  0.000414)
 24:     71.99   0.000033    0.17  0.000144  (-0.006668  0.009848 -0.001472)
 25:     76.10   0.000009    0.04  0.000036  (-0.004403 -0.004059  0.000340)
 26:     76.65   0.000048    0.24  0.000195  ( 0.011600  0.007770  0.000387)
 27:     82.17   0.000108    0.55  0.000410  (-0.001039 -0.000413  0.020211)
 28:     86.15   0.000021    0.10  0.000075  (-0.005988 -0.003613 -0.005068)
 29:     88.00   0.000023    0.12  0.000082  (-0.007403 -0.005214 -0.000616)
 30:     90.44   0.000627    3.17  0.002162  ( 0.041222  0.021365 -0.002533)
 31:     96.45   0.000102    0.52  0.000331  ( 0.000807 -0.018125 -0.001437)
 32:    100.38   0.000006    0.03  0.000020  (-0.002930 -0.000539 -0.003312)
 33:    104.30   0.000013    0.07  0.000039  (-0.001996 -0.000843 -0.005852)
 34:    106.62   0.000248    1.25  0.000726  (-0.022780 -0.014276  0.001825)
 35:    113.79   0.000010    0.05  0.000028  (-0.000991 -0.000550 -0.005139)
 36:    119.89   0.000059    0.30  0.000153  ( 0.006705 -0.010406 -0.000126)
 37:    121.71   0.000155    0.78  0.000398  ( 0.000507  0.003625 -0.019616)
 38:    128.77   0.000298    1.51  0.000723  ( 0.005356  0.025250  0.007531)
 39:    131.09   0.000739    3.73  0.001758  ( 0.000480 -0.004757  0.041658)
 40:    134.81   0.000250    1.26  0.000579  ( 0.021566  0.010484 -0.002041)
 41:    141.09   0.000081    0.41  0.000179  ( 0.000838  0.001071  0.013305)
 42:    148.08   0.000006    0.03  0.000012  ( 0.001323 -0.001992  0.002550)
 43:    151.49   0.000074    0.37  0.000151  (-0.011303  0.002110  0.004391)
 44:    152.75   0.000120    0.61  0.000245  (-0.002752 -0.000114 -0.015411)
 45:    155.57   0.000001    0.00  0.000001  ( 0.000440 -0.000462 -0.000814)
 46:    172.23   0.000122    0.62  0.000221  (-0.012643  0.007679 -0.001364)
 47:    174.37   0.000222    1.12  0.000398  (-0.001351 -0.001090 -0.019865)
 48:    177.63   0.000179    0.91  0.000315  (-0.002914  0.004268  0.016983)
 49:    180.62   0.001198    6.06  0.002070  ( 0.022914 -0.039307 -0.000235)
 50:    190.84   0.003594   18.16  0.005877  ( 0.035493  0.067935  0.001612)
 51:    196.24   0.000497    2.51  0.000790  (-0.017329  0.022078 -0.001616)
 52:    203.19   0.004050   20.47  0.006220  (-0.001563  0.001963 -0.078826)
 53:    211.86   0.000330    1.67  0.000486  (-0.000083  0.000551 -0.022048)
 54:    226.58   0.000195    0.99  0.000269  ( 0.005825  0.015309 -0.000560)
 55:    240.20   0.000825    4.17  0.001072  ( 0.001367  0.008966 -0.031456)
 56:    241.45   0.001266    6.40  0.001636  ( 0.011001  0.038700  0.004166)
 57:    246.98   0.000356    1.80  0.000450  ( 0.004074 -0.000425 -0.020810)
 58:    247.40   0.000218    1.10  0.000275  ( 0.016316 -0.001405  0.002539)
 59:    254.04   0.000659    3.33  0.000810  ( 0.001190  0.001062  0.028418)
 60:    255.13   0.001322    6.68  0.001617  (-0.038048 -0.012786  0.002369)
 61:    257.42   0.000009    0.04  0.000010  (-0.000491 -0.001450 -0.002848)
 62:    260.61   0.000207    1.04  0.000248  ( 0.002800 -0.015456 -0.000925)
```

63:	269.96	0.000268	1.35	0.000309	(-0.002331 0.001117 -0.017395)
64:	271.31	0.000892	4.51	0.001025	(-0.030787 -0.008608 0.001882)
65:	274.98	0.000250	1.26	0.000283	(-0.003663 -0.001274 -0.016380)
66:	278.00	0.001214	6.14	0.001363	(-0.030510 -0.020698 0.001990)
67:	282.55	0.000055	0.28	0.000061	(0.000090 0.000161 -0.007823)
68:	291.08	0.000350	1.77	0.000375	(0.006556 -0.018205 -0.000617)
69:	299.66	0.000130	0.66	0.000135	(0.000392 0.001219 -0.011556)
70:	307.41	0.000026	0.13	0.000026	(-0.003769 -0.000945 -0.003351)
71:	309.64	0.001038	5.24	0.001046	(-0.001291 -0.000919 -0.032302)
72:	314.55	0.000804	4.06	0.000797	(0.009574 0.026552 -0.000789)
73:	315.84	0.000407	2.06	0.000402	(-0.000544 -0.001306 0.019997)
74:	330.13	0.000048	0.24	0.000046	(0.004690 0.004756 -0.001027)
75:	330.25	0.000222	1.12	0.000209	(0.010971 0.009420 -0.000535)
76:	334.94	0.000424	2.14	0.000395	(0.013367 -0.014685 -0.000783)
77:	337.59	0.000005	0.03	0.000005	(0.000766 -0.001963 0.000535)
78:	345.58	0.002360	11.92	0.002131	(-0.022738 0.039826 0.005261)
79:	347.18	0.001094	5.53	0.000984	(-0.005170 0.005829 -0.030381)
80:	349.41	0.002162	10.93	0.001931	(-0.028025 -0.033760 0.002490)
81:	355.86	0.001007	5.09	0.000883	(0.002113 -0.000606 0.029640)
82:	360.36	0.002069	10.46	0.001792	(0.041437 -0.008328 -0.002379)
83:	367.97	0.000222	1.12	0.000188	(0.009367 0.002870 0.009604)
84:	369.66	0.001987	10.04	0.001677	(0.036019 0.019168 -0.003532)
85:	382.87	0.000272	1.37	0.000222	(-0.005092 0.009504 -0.010270)
86:	384.12	0.000348	1.76	0.000283	(-0.008729 0.012958 0.006233)
87:	397.41	0.000002	0.01	0.000002	(-0.000432 0.000706 0.001055)
88:	408.29	0.003539	17.89	0.002705	(-0.039463 0.033830 0.001844)
89:	417.58	0.000244	1.23	0.000182	(-0.002041 0.013335 0.000413)
90:	420.94	0.000014	0.07	0.000010	(-0.000463 0.000449 -0.003105)
91:	422.24	0.000049	0.25	0.000036	(-0.004858 -0.003511 0.000589)
92:	423.58	0.000042	0.21	0.000031	(0.000631 0.001487 0.005336)
93:	430.50	0.000057	0.29	0.000041	(0.000450 -0.001552 0.006205)
94:	430.66	0.000053	0.27	0.000038	(0.001549 -0.001921 -0.005673)
95:	434.16	0.000396	2.00	0.000284	(-0.001516 0.006059 -0.015667)
96:	435.27	0.001969	9.95	0.001412	(-0.018062 0.032947 0.000272)
97:	436.45	0.000264	1.33	0.000188	(0.008735 0.010510 0.001306)
98:	438.23	0.000004	0.02	0.000003	(0.000025 0.001757 -0.000150)
99:	443.07	0.003765	19.03	0.002652	(0.025822 0.044250 0.005192)
100:	447.49	0.004788	24.19	0.003339	(-0.000104 -0.002069 0.057744)
101:	468.85	0.001492	7.54	0.000993	(0.003630 -0.001759 -0.031250)
102:	470.58	0.001376	6.95	0.000913	(-0.026452 0.014271 -0.003041)
103:	484.62	0.001969	9.95	0.001268	(-0.016489 -0.031525 0.001520)
104:	487.02	0.002214	11.19	0.001419	(0.000629 0.000197 0.037660)
105:	492.58	0.000009	0.05	0.000006	(-0.000302 -0.001604 -0.001815)
106:	498.98	0.009858	49.82	0.006165	(-0.067092 0.040742 0.001981)
107:	521.02	0.000039	0.20	0.000023	(0.000428 -0.000800 -0.004758)
108:	521.79	0.000026	0.13	0.000016	(-0.002578 0.002975 -0.000495)
109:	534.88	0.000063	0.32	0.000037	(-0.003897 0.000315 0.004623)
110:	535.65	0.000106	0.53	0.000062	(-0.007425 -0.000703 -0.002450)
111:	540.22	0.001445	7.30	0.000834	(0.000295 0.000645 0.028879)
112:	540.81	0.000080	0.41	0.000046	(0.001613 -0.005490 0.003689)
113:	590.29	0.001303	6.58	0.000689	(-0.007533 0.017357 -0.018185)
114:	591.15	0.001248	6.31	0.000659	(-0.003754 0.011624 0.022579)
115:	595.79	0.000102	0.52	0.000054	(-0.002013 0.002321 0.006643)
116:	598.58	0.002213	11.18	0.001154	(0.023402 -0.024614 -0.000242)
117:	621.44	0.000632	3.19	0.000317	(0.015221 -0.000493 -0.009241)
118:	622.02	0.002942	14.87	0.001476	(-0.005311 0.000637 -0.038043)
119:	624.30	0.004600	23.24	0.002299	(-0.014310 -0.045763 -0.000417)
120:	624.85	0.000837	4.23	0.000418	(0.006403 0.019236 -0.002679)
121:	634.88	0.000602	3.04	0.000296	(0.016976 0.002742 -0.000043)
122:	635.46	0.000087	0.44	0.000043	(0.002113 0.001180 0.006058)
123:	635.76	0.000111	0.56	0.000054	(-0.005436 -0.004070 0.002866)
124:	635.83	0.001521	7.68	0.000746	(-0.020650 -0.017874 0.000654)
125:	636.18	0.000072	0.36	0.000035	(-0.004390 -0.004005 -0.000270)
126:	636.39	0.000232	1.17	0.000114	(-0.001040 -0.001050 0.010571)
127:	636.48	0.000005	0.03	0.000003	(0.000006 0.000066 0.001620)
128:	637.34	0.000054	0.27	0.000027	(0.004024 0.003158 -0.000620)
129:	642.37	0.000007	0.03	0.000003	(0.001765 0.000035 0.000383)
130:	643.69	0.001239	6.26	0.000600	(-0.023386 0.007275 0.000820)
131:	645.67	0.002551	12.89	0.001233	(0.013070 0.032584 0.000716)
132:	646.38	0.000383	1.94	0.000185	(0.000962 -0.013255 -0.002878)
133:	646.95	0.000417	2.11	0.000201	(0.006950 0.001209 -0.012304)
134:	647.41	0.003574	18.06	0.001723	(0.035200 -0.021978 0.000806)
135:	661.93	0.000181	0.91	0.000085	(-0.002641 0.002688 0.008432)
136:	662.66	0.002063	10.43	0.000972	(0.023864 -0.020055 -0.000005)
137:	674.42	0.000054	0.27	0.000025	(-0.001349 0.001370 0.004595)
138:	675.46	0.002446	12.36	0.001130	(0.027405 -0.019446 -0.000901)
139:	692.58	0.000588	2.97	0.000265	(-0.015820 -0.003008 -0.002384)

140:	692.69	0.000446	2.26	0.000201	(0.013419	0.002910	-0.003548)
141:	712.09	0.003987	20.15	0.001747	(-0.011238	-0.040225	-0.001712)
142:	712.37	0.000864	4.37	0.000379	(-0.000738	-0.007608	0.017891)
143:	720.53	0.022103	111.70	0.009573	(0.028236	0.093665	0.001544)
144:	723.54	0.001285	6.50	0.000554	(0.003987	0.006055	-0.022402)
145:	725.00	0.024308	122.84	0.010463	(-0.100675	-0.017279	0.005383)
146:	726.44	0.011282	57.02	0.004847	(0.006250	0.001027	0.069329)
147:	728.93	0.000151	0.76	0.000065	(-0.005584	-0.004903	0.003081)
148:	729.86	0.011077	55.98	0.004736	(-0.000805	0.000583	-0.068813)
149:	735.18	0.003648	18.43	0.001548	(0.038872	-0.000389	-0.006098)
150:	735.67	0.001917	9.69	0.000813	(0.027600	0.000753	0.007131)
151:	739.98	0.001585	8.01	0.000668	(-0.021373	-0.014545	-0.000024)
152:	740.67	0.001289	6.51	0.000543	(-0.000490	-0.001064	0.023271)
153:	747.69	0.000646	3.26	0.000270	(0.010231	-0.000385	-0.012834)
154:	747.72	0.004572	23.11	0.001908	(-0.043653	-0.000503	-0.001529)
155:	751.76	0.033853	171.08	0.014053	(-0.115617	-0.026051	0.002609)
156:	751.79	0.002976	15.04	0.001235	(-0.002039	-0.000689	0.035079)
157:	760.06	0.000464	2.35	0.000191	(0.003241	0.000580	0.013411)
158:	760.31	0.000169	0.85	0.000069	(0.002381	0.000623	-0.007951)
159:	762.92	0.002896	14.64	0.001185	(0.006612	0.032794	0.008095)
160:	763.23	0.001406	7.11	0.000575	(-0.002124	-0.014862	0.018696)
161:	782.32	0.007066	35.71	0.002819	(0.035907	-0.033819	0.019638)
162:	783.07	0.010525	53.19	0.004195	(-0.045660	0.041502	0.019679)
163:	789.00	0.004050	20.47	0.001602	(-0.013665	-0.003146	0.037488)
164:	789.43	0.005302	26.79	0.002096	(-0.043321	-0.006445	-0.013329)
165:	792.27	0.000539	2.72	0.000212	(0.009708	0.002472	-0.010577)
166:	794.29	0.006996	35.35	0.002749	(-0.046785	-0.023656	-0.000229)
167:	804.26	0.002851	14.41	0.001106	(-0.016969	0.017177	0.022877)
168:	805.01	0.005027	25.41	0.001949	(0.031586	-0.028829	0.010958)
169:	817.24	0.000480	2.42	0.000183	(0.008391	-0.010362	-0.002315)
170:	818.61	0.000622	3.15	0.000237	(-0.001098	-0.001648	-0.015275)
171:	819.51	0.005078	25.66	0.001934	(0.016367	0.040691	-0.003144)
172:	821.98	0.004792	24.22	0.001819	(0.002504	0.002964	0.042478)
173:	869.22	0.000101	0.51	0.000036	(0.003851	0.004636	-0.000188)
174:	872.31	0.000086	0.43	0.000031	(-0.000536	0.000138	-0.005506)
175:	873.95	0.000027	0.13	0.000010	(0.002806	0.000026	-0.001289)
176:	876.60	0.000016	0.08	0.000006	(0.000633	0.000408	0.002303)
177:	883.61	0.000059	0.30	0.000021	(0.003936	-0.001087	-0.002033)
178:	883.97	0.000051	0.26	0.000018	(-0.002976	0.001231	-0.002786)
179:	886.45	0.000010	0.05	0.000003	(0.000153	-0.000021	0.001836)
180:	886.67	0.000020	0.10	0.000007	(0.002627	-0.000036	-0.000094)
181:	890.08	0.000009	0.05	0.000003	(0.001209	0.000735	0.001078)
182:	890.56	0.000514	2.60	0.000180	(-0.011113	-0.007499	0.000645)
183:	891.51	0.000076	0.38	0.000027	(-0.003557	-0.000335	-0.003712)
184:	891.82	0.000055	0.28	0.000019	(-0.002598	-0.000029	0.003513)
185:	933.48	0.008759	44.26	0.002928	(-0.053279	-0.009435	0.000694)
186:	938.90	0.002690	13.59	0.000894	(0.000634	0.001751	-0.029842)
187:	944.75	0.006944	35.09	0.002294	(-0.009185	0.046788	-0.004505)
188:	948.29	0.004757	24.04	0.001565	(0.000556	-0.007106	-0.038919)
189:	953.55	0.000250	1.26	0.000082	(0.008449	0.002928	0.001304)
190:	954.47	0.000159	0.80	0.000052	(0.001102	-0.000311	0.007122)
191:	957.19	0.005218	26.37	0.001701	(0.041009	0.003946	-0.001933)
192:	959.98	0.000144	0.73	0.000047	(0.002041	0.001844	0.006271)
193:	960.78	0.003166	16.00	0.001028	(-0.020631	-0.020628	0.013314)
194:	961.37	0.002132	10.78	0.000692	(0.013066	0.011111	0.019949)
195:	966.63	0.000916	4.63	0.000296	(-0.000609	-0.013136	-0.011083)
196:	967.12	0.000706	3.57	0.000228	(-0.001091	0.010682	-0.010604)
197:	968.25	0.000002	0.01	0.000001	(0.000247	-0.000276	0.000743)
198:	970.33	0.000900	4.55	0.000290	(-0.014144	0.009410	0.000992)
199:	984.58	0.000284	1.43	0.000090	(-0.003731	-0.001768	0.008539)
200:	986.12	0.002208	11.16	0.000699	(0.023459	0.012161	0.000750)
201:	1000.08	0.000091	0.46	0.000028	(0.004548	0.002725	-0.000490)
202:	1000.17	0.000055	0.28	0.000017	(0.000467	0.000236	0.004098)
203:	1002.33	0.001473	7.44	0.000459	(0.001922	0.006464	-0.020323)
204:	1003.30	0.003201	16.18	0.000996	(0.011067	0.029096	0.005161)
205:	1010.76	0.000010	0.05	0.000003	(-0.000703	0.001438	-0.000697)
206:	1011.60	0.000019	0.09	0.000006	(0.001005	-0.002138	0.000430)
207:	1015.55	0.000344	1.74	0.000106	(0.008201	-0.005430	-0.002993)
208:	1016.44	0.000239	1.21	0.000074	(0.003101	-0.001981	0.007745)
209:	1022.52	0.000339	1.71	0.000103	(-0.001926	-0.009450	0.003227)
210:	1023.24	0.000313	1.58	0.000095	(0.002032	0.008783	0.003755)
211:	1026.44	0.000664	3.36	0.000202	(-0.000975	0.000589	-0.014162)
212:	1026.75	0.001187	6.00	0.000361	(-0.004384	0.018461	0.000803)
213:	1026.88	0.000268	1.35	0.000081	(0.000054	0.001917	-0.008812)
214:	1027.22	0.000464	2.35	0.000141	(0.010424	0.005669	0.000484)
215:	1029.13	0.000646	3.27	0.000196	(-0.003186	-0.001491	-0.013550)
216:	1029.23	0.001923	9.72	0.000583	(0.021637	0.010449	-0.002362)

217:	1031.06	0.001026	5.18	0.000311	(-0.015563	0.008235	0.000694)
218:	1031.97	0.000031	0.16	0.000009	(-0.000217	0.000303	-0.003044)
219:	1032.26	0.000063	0.32	0.000019	(0.003397	-0.002682	0.000456)
220:	1033.77	0.000093	0.47	0.000028	(0.000350	-0.000867	0.005216)
221:	1035.89	0.000108	0.54	0.000032	(-0.004624	0.003273	0.000553)
222:	1036.10	0.000072	0.36	0.000022	(0.003630	-0.002890	0.000231)
223:	1041.43	0.000121	0.61	0.000036	(0.000720	-0.000164	-0.005973)
224:	1041.52	0.000098	0.49	0.000029	(0.002831	-0.001587	0.004334)
225:	1044.56	0.000272	1.38	0.000081	(-0.008434	0.002858	-0.001425)
226:	1044.99	0.000138	0.70	0.000041	(-0.002723	0.001195	0.005691)
227:	1045.92	0.000528	2.67	0.000157	(-0.012456	-0.000267	0.001486)
228:	1045.98	0.000091	0.46	0.000027	(0.002872	0.000199	0.004335)
229:	1057.21	0.005518	27.89	0.001629	(0.037291	-0.015201	0.002663)
230:	1058.53	0.001275	6.44	0.000376	(-0.005982	0.002762	0.018233)
231:	1067.95	0.002269	11.47	0.000663	(0.004709	-0.025254	0.001747)
232:	1068.36	0.000990	5.00	0.000289	(0.000456	-0.002680	-0.016784)
233:	1075.85	0.001980	10.01	0.000574	(0.021703	0.001586	-0.010043)
234:	1076.25	0.003199	16.17	0.000928	(-0.029726	-0.001011	-0.006553)
235:	1079.96	0.000915	4.62	0.000264	(0.007840	-0.014172	0.001409)
236:	1080.67	0.000669	3.38	0.000193	(-0.000583	-0.001484	-0.013808)
237:	1082.08	0.000409	2.07	0.000118	(0.008534	-0.005949	0.003135)
238:	1082.37	0.002604	13.16	0.000751	(0.023117	-0.014236	-0.003703)
239:	1084.70	0.000439	2.22	0.000126	(-0.000566	0.001172	0.011159)
240:	1085.01	0.000534	2.70	0.000154	(0.008125	-0.009358	-0.000048)
241:	1085.37	0.000090	0.46	0.000026	(-0.001850	0.002709	-0.003900)
242:	1085.60	0.000152	0.77	0.000044	(-0.006450	-0.000752	-0.001263)
243:	1099.65	0.011737	59.31	0.003331	(0.057043	0.008638	-0.001479)
244:	1101.05	0.000207	1.05	0.000059	(0.001972	0.000741	0.007363)
245:	1102.36	0.000280	1.41	0.000079	(0.005537	0.002980	0.006296)
246:	1102.56	0.000570	2.88	0.000161	(-0.002332	-0.002071	0.012308)
247:	1103.75	0.000114	0.58	0.000032	(0.002698	-0.002148	0.004523)
248:	1104.85	0.001005	5.08	0.000284	(-0.015426	0.006684	0.001105)
249:	1110.90	0.002239	11.31	0.000629	(0.017153	-0.014257	0.011464)
250:	1111.31	0.001440	7.28	0.000404	(0.010895	-0.006946	-0.015405)
251:	1113.79	0.013613	68.80	0.003814	(0.024042	0.056882	0.000825)
252:	1114.60	0.017407	87.97	0.004873	(0.000419	-0.001999	0.069780)
253:	1121.49	0.000047	0.24	0.000013	(0.001881	-0.000780	-0.003001)
254:	1122.16	0.001307	6.61	0.000364	(-0.012703	0.014204	-0.000669)
255:	1124.61	0.003927	19.85	0.001090	(0.021755	0.022568	-0.010351)
256:	1125.21	0.004434	22.41	0.001230	(-0.008063	-0.008383	-0.033083)
257:	1130.82	0.000502	2.54	0.000138	(-0.000486	0.000731	-0.011733)
258:	1131.45	0.000501	2.53	0.000138	(-0.000669	0.011628	0.001553)
259:	1138.53	0.004196	21.20	0.001150	(0.025348	-0.022525	-0.000404)
260:	1141.87	0.000760	3.84	0.000208	(-0.000427	0.000544	0.014400)
261:	1149.46	0.008078	40.82	0.002193	(0.001091	-0.001331	0.046798)
262:	1150.34	0.003113	15.73	0.000844	(-0.001562	0.028953	0.001917)
263:	1166.76	0.002970	15.01	0.000794	(0.004216	0.027765	0.002361)
264:	1168.81	0.013652	68.99	0.003645	(0.001458	-0.001442	0.060338)
265:	1174.65	0.006223	31.45	0.001653	(-0.005345	-0.040304	-0.000427)
266:	1176.34	0.000187	0.95	0.000050	(-0.000114	-0.002977	-0.006385)
267:	1180.55	0.000032	0.16	0.000008	(0.000120	-0.002560	-0.001362)
268:	1180.93	0.001645	8.31	0.000435	(-0.015391	0.014007	-0.001297)
269:	1181.65	0.002568	12.98	0.000678	(-0.006609	-0.024494	0.005881)
270:	1181.88	0.001498	7.57	0.000395	(-0.001842	-0.015889	-0.011813)
271:	1184.13	0.000651	3.29	0.000172	(0.000118	0.000578	-0.013085)
272:	1184.56	0.000165	0.83	0.000043	(-0.000362	0.006398	0.001529)
273:	1186.45	0.001938	9.80	0.000510	(0.000199	0.022526	0.001543)
274:	1187.81	0.001219	6.16	0.000320	(0.000484	0.000059	0.017888)
275:	1205.22	0.000387	1.96	0.000100	(0.008682	0.004835	0.001244)
276:	1205.54	0.000116	0.58	0.000030	(-0.001628	-0.000836	0.005153)
277:	1207.33	0.000302	1.53	0.000078	(-0.003218	0.003795	0.007304)
278:	1208.06	0.000401	2.03	0.000104	(-0.006155	0.007111	-0.003893)
279:	1212.49	0.000506	2.56	0.000130	(-0.007774	-0.005548	0.006251)
280:	1212.78	0.000577	2.91	0.000148	(-0.002092	-0.005161	-0.010833)
281:	1215.79	0.007007	35.41	0.001799	(0.030302	-0.029672	0.000006)
282:	1216.57	0.000188	0.95	0.000048	(-0.003977	0.005226	0.002244)
283:	1221.23	0.000109	0.55	0.000028	(-0.000483	-0.004908	-0.001858)
284:	1221.77	0.001079	5.45	0.000276	(0.008248	0.014401	-0.000409)
285:	1229.44	0.002712	13.71	0.000688	(-0.002529	0.001388	0.026078)
286:	1230.28	0.003314	16.75	0.000841	(-0.028256	0.005697	-0.003137)
287:	1233.32	0.000737	3.72	0.000186	(0.001873	-0.002483	-0.013294)
288:	1237.53	0.000541	2.73	0.000136	(0.009297	-0.007062	-0.000091)
289:	1245.96	0.011094	56.06	0.002779	(-0.000851	0.002269	-0.052656)
290:	1246.11	0.007844	39.64	0.001964	(0.004977	0.043987	0.002216)
291:	1250.38	0.000869	4.39	0.000217	(0.006234	0.013336	-0.000500)
292:	1250.95	0.000173	0.87	0.000043	(0.002179	0.005663	0.002522)
293:	1275.73	0.000213	1.07	0.000052	(-0.003624	0.005723	0.002479)

294:	1275.87	0.000808	4.09	0.000198	(-0.000920 0.001017 -0.013995)
295:	1278.94	0.007536	38.08	0.001839	(0.038457 0.018885 -0.001781)
296:	1280.41	0.004157	21.01	0.001013	(0.003158 -0.000389 0.031670)
297:	1285.88	0.002910	14.70	0.000706	(0.006151 -0.013498 -0.022048)
298:	1286.96	0.016823	85.02	0.004079	(0.027036 -0.057742 0.003782)
299:	1306.40	0.011450	57.86	0.002735	(-0.003193 -0.007859 0.051605)
300:	1307.02	0.003428	17.32	0.000819	(-0.013542 -0.022149 -0.012024)
301:	1312.89	0.009021	45.59	0.002144	(-0.032158 -0.032965 -0.004826)
302:	1314.96	0.001768	8.94	0.000420	(-0.005033 -0.006937 0.018606)
303:	1323.05	0.000937	4.73	0.000221	(-0.012466 0.008094 0.000124)
304:	1323.32	0.000198	1.00	0.000047	(0.005109 -0.004338 -0.001347)
305:	1339.40	0.000852	4.30	0.000198	(-0.012360 -0.006231 0.002623)
306:	1340.43	0.000401	2.03	0.000093	(-0.004784 -0.002686 -0.007959)
307:	1341.95	0.000505	2.55	0.000117	(-0.001224 0.008532 0.006572)
308:	1342.39	0.001808	9.14	0.000420	(0.001630 -0.020186 0.003183)
309:	1345.16	0.001026	5.19	0.000238	(-0.011605 -0.009982 -0.001928)
310:	1346.17	0.002392	12.09	0.000555	(-0.000403 -0.001180 0.023517)
311:	1346.68	0.001095	5.53	0.000254	(0.006141 -0.014639 -0.001327)
312:	1347.51	0.000507	2.56	0.000118	(-0.001103 0.001316 -0.010704)
313:	1361.48	0.000061	0.31	0.000014	(0.000135 0.000575 0.003706)
314:	1361.53	0.000041	0.21	0.000010	(-0.000727 -0.000225 0.002989)
315:	1363.44	0.000829	4.19	0.000190	(-0.013188 0.003835 0.001058)
316:	1363.62	0.000723	3.65	0.000165	(0.012388 -0.003405 -0.000529)
317:	1364.64	0.003577	18.07	0.000818	(0.026836 -0.009445 -0.002914)
318:	1365.48	0.001052	5.32	0.000241	(0.014157 -0.003999 0.004909)
319:	1368.28	0.000194	0.98	0.000044	(-0.002608 0.005776 0.001997)
320:	1368.59	0.000392	1.98	0.000089	(-0.004057 0.001749 -0.008353)
321:	1369.84	0.003969	20.06	0.000904	(0.030008 -0.000632 -0.001790)
322:	1370.63	0.001604	8.11	0.000365	(0.019077 -0.000119 -0.001100)
323:	1383.59	0.005219	26.37	0.001177	(0.000306 0.000931 -0.034294)
324:	1384.42	0.001768	8.94	0.000399	(-0.019091 -0.005545 -0.001829)
325:	1387.79	0.002094	10.58	0.000471	(0.011817 0.017954 0.002963)
326:	1388.92	0.000998	5.04	0.000224	(0.001178 0.002094 -0.014779)
327:	1405.46	0.001913	9.67	0.000425	(-0.013646 0.015411 -0.001024)
328:	1405.75	0.002447	12.37	0.000543	(0.014815 -0.017917 -0.001647)
329:	1438.76	0.001991	10.06	0.000432	(-0.010385 -0.008265 0.015991)
330:	1439.03	0.002438	12.32	0.000529	(-0.007712 -0.005604 -0.020924)
331:	1444.06	0.000422	2.13	0.000091	(0.001828 0.000446 0.009360)
332:	1444.14	0.002843	14.37	0.000614	(0.023663 0.007320 -0.000846)
333:	1450.63	0.000238	1.20	0.000051	(-0.007036 -0.000525 -0.001181)
334:	1450.75	0.000138	0.70	0.000030	(0.005311 0.000418 -0.001149)
335:	1467.64	0.003948	19.95	0.000839	(-0.000018 0.028974 -0.000058)
336:	1467.98	0.000541	2.73	0.000115	(0.000267 -0.010719 0.000008)
337:	1482.55	0.003345	16.90	0.000704	(0.015787 0.010108 -0.018779)
338:	1483.48	0.003865	19.53	0.000813	(0.011990 0.007672 0.024707)
339:	1488.88	0.004789	24.20	0.001004	(0.027830 -0.005956 0.013918)
340:	1489.07	0.003675	18.57	0.000770	(0.023362 -0.003993 -0.014440)
341:	1490.10	0.000935	4.72	0.000196	(0.012473 -0.006066 0.001844)
342:	1490.71	0.001989	10.05	0.000416	(-0.018884 0.007076 0.003126)
343:	1498.29	0.000062	0.31	0.000013	(0.000976 0.002826 0.001977)
344:	1498.52	0.000110	0.56	0.000023	(0.000760 -0.004709 0.000445)
345:	1501.99	0.001678	8.48	0.000349	(-0.013828 -0.012431 0.001682)
346:	1502.19	0.001651	8.34	0.000343	(-0.014065 -0.011916 -0.001769)
347:	1503.43	0.005491	27.75	0.001140	(0.013287 0.031035 -0.000199)
348:	1504.53	0.000045	0.23	0.000009	(0.000802 0.001519 -0.002522)
349:	1505.05	0.001758	8.88	0.000365	(-0.000845 0.000412 -0.019069)
350:	1505.58	0.008279	41.84	0.001716	(-0.004714 -0.000032 0.041156)
351:	1505.86	0.004020	20.32	0.000833	(-0.009411 0.000714 -0.027278)
352:	1506.09	0.000739	3.74	0.000153	(0.011221 -0.004998 -0.001510)
353:	1522.13	0.003073	15.53	0.000630	(0.005350 0.015218 -0.019230)
354:	1523.35	0.005033	25.43	0.001031	(-0.001523 -0.011212 -0.030049)
355:	1532.25	0.005197	26.27	0.001059	(-0.017105 -0.018059 -0.020971)
356:	1533.03	0.005324	26.90	0.001084	(-0.011649 -0.014212 0.027314)
357:	1539.27	0.017602	88.95	0.003569	(0.056004 -0.016274 0.012934)
358:	1540.31	0.010696	54.05	0.002167	(-0.039580 0.012432 0.021114)
359:	1552.20	0.009478	47.90	0.001905	(0.026109 -0.034766 -0.003892)
360:	1552.80	0.025802	130.39	0.005185	(-0.043361 0.057484 -0.000897)
361:	1553.15	0.009268	46.83	0.001862	(0.016188 -0.040000 -0.000032)
362:	1553.26	0.000269	1.36	0.000054	(-0.005195 0.004837 -0.001915)
363:	1555.17	0.005404	27.31	0.001084	(0.016627 -0.021177 0.018959)
364:	1555.31	0.006557	33.13	0.001316	(-0.017666 0.024405 0.020195)
365:	1561.21	0.000239	1.21	0.000048	(-0.004621 0.005086 0.000790)
366:	1561.70	0.000081	0.41	0.000016	(0.000934 -0.001778 0.003497)
367:	1562.86	0.001243	6.28	0.000248	(0.015199 0.004144 0.000316)
368:	1563.10	0.000271	1.37	0.000054	(0.002337 0.000885 -0.006919)
369:	1653.01	0.001731	8.75	0.000327	(-0.011415 -0.005162 0.013031)
370:	1653.74	0.001731	8.75	0.000327	(-0.012020 -0.004805 -0.012614)

371:	1671.42	0.001390	7.02	0.000260	(0.005861 -0.008499 0.012366)
372:	1672.10	0.001438	7.27	0.000268	(0.004730 -0.008145 -0.013405)
373:	1687.50	0.000243	1.23	0.000045	(0.003171 -0.003887 0.004444)
374:	1687.57	0.000240	1.21	0.000044	(-0.002906 0.004066 0.004398)
375:	1690.22	0.000546	2.76	0.000101	(0.002660 0.009601 -0.001232)
376:	1690.75	0.000125	0.63	0.000023	(-0.001197 -0.003385 -0.003178)
377:	1692.62	0.000136	0.69	0.000025	(0.002903 -0.001219 -0.003883)
378:	1692.75	0.000176	0.89	0.000032	(-0.002720 0.000881 -0.004921)
379:	1693.41	0.000118	0.59	0.000022	(0.003706 -0.002810 0.000196)
380:	1693.57	0.000034	0.17	0.000006	(-0.001328 0.001398 0.001575)
381:	1710.18	0.000915	4.62	0.000167	(-0.004715 0.012028 -0.000105)
382:	1710.44	0.000073	0.37	0.000013	(0.000920 -0.003096 -0.001695)
383:	1712.25	0.000154	0.78	0.000028	(0.000163 -0.000108 0.005302)
384:	1713.08	0.000019	0.09	0.000003	(0.001798 -0.000381 0.000147)
385:	1713.70	0.000127	0.64	0.000023	(0.004791 0.000003 -0.000334)
386:	1713.84	0.000024	0.12	0.000004	(0.000348 -0.000170 0.002075)
387:	1714.75	0.000650	3.28	0.000118	(0.010863 0.000426 -0.000326)
388:	1715.09	0.000018	0.09	0.000003	(-0.000001 -0.000166 -0.001811)
389:	3111.01	0.001848	9.34	0.000185	(0.012525 -0.004263 -0.003218)
390:	3111.18	0.001710	8.64	0.000172	(0.012256 -0.003881 0.002498)
391:	3127.08	0.000477	2.41	0.000048	(0.000081 -0.006348 -0.002704)
392:	3127.25	0.000507	2.56	0.000051	(0.000331 -0.006561 0.002734)
393:	3161.05	0.001175	5.94	0.000116	(0.003496 -0.010174 -0.000517)
394:	3161.26	0.001337	6.75	0.000132	(0.003535 -0.010927 0.000219)
395:	3174.56	0.000239	1.21	0.000023	(0.000930 0.004742 -0.000338)
396:	3175.15	0.000245	1.24	0.000024	(-0.000784 -0.004831 -0.000375)
397:	3180.66	0.000993	5.02	0.000097	(0.004776 0.006484 0.005708)
398:	3181.42	0.001306	6.60	0.000128	(-0.006205 -0.008534 0.004091)
399:	3195.32	0.000096	0.49	0.000009	(0.003020 -0.000151 -0.000517)
400:	3195.43	0.000109	0.55	0.000011	(0.003231 -0.000148 0.000444)
401:	3214.33	0.000596	3.01	0.000058	(-0.000146 0.006989 -0.003000)
402:	3215.00	0.000662	3.34	0.000064	(0.000651 0.007377 0.003065)
403:	3215.07	0.000494	2.50	0.000048	(0.001758 -0.005321 0.004069)
404:	3215.67	0.000447	2.26	0.000043	(0.001467 -0.004222 -0.004840)
405:	3218.07	0.000448	2.26	0.000043	(-0.002342 -0.005897 -0.001775)
406:	3218.37	0.000364	1.84	0.000035	(-0.002112 -0.005061 0.002278)
407:	3223.68	0.000186	0.94	0.000018	(0.000401 0.002266 -0.003561)
408:	3223.79	0.000548	2.77	0.000053	(0.006522 -0.001169 -0.003030)
409:	3223.92	0.000554	2.80	0.000054	(-0.003905 0.001646 -0.005975)
410:	3224.20	0.000327	1.65	0.000032	(0.000171 -0.002510 -0.005030)
411:	3225.80	0.000062	0.31	0.000006	(-0.001944 -0.001366 0.000590)
412:	3226.27	0.000094	0.48	0.000009	(0.002731 0.000768 0.001031)
413:	3230.56	0.000326	1.65	0.000031	(0.005469 0.000234 -0.001237)
414:	3230.77	0.002271	11.47	0.000219	(-0.000901 0.000619 -0.014769)
415:	3234.20	0.000256	1.30	0.000025	(0.000079 0.004840 -0.001145)
416:	3234.33	0.000177	0.90	0.000017	(0.000517 0.004048 0.000678)
417:	3234.44	0.000904	4.57	0.000087	(0.001077 0.009214 0.001075)
418:	3234.59	0.001020	5.15	0.000098	(-0.000689 0.000787 0.009862)
419:	3234.60	0.000234	1.18	0.000023	(-0.000257 -0.002082 -0.004266)
420:	3234.95	0.000372	1.88	0.000036	(0.000268 -0.002348 0.005502)
421:	3235.75	0.000362	1.83	0.000035	(0.002474 -0.002312 0.004839)
422:	3236.34	0.000229	1.16	0.000022	(-0.001691 0.002010 0.003902)
423:	3238.35	0.001691	8.54	0.000163	(0.010305 -0.007483 -0.000848)
424:	3239.41	0.000171	0.87	0.000016	(-0.002231 0.001217 -0.003167)
425:	3245.02	0.001042	5.27	0.000100	(-0.000606 -0.009480 -0.003164)
426:	3245.37	0.000958	4.84	0.000092	(-0.000627 -0.009277 0.002379)
427:	3245.42	0.000373	1.88	0.000036	(-0.005273 0.002366 -0.001562)
428:	3245.68	0.001593	8.05	0.000153	(-0.012099 -0.002403 0.001014)
429:	3247.25	0.000310	1.56	0.000030	(-0.003949 0.000016 -0.003761)
430:	3247.27	0.001137	5.75	0.000109	(0.001078 0.000104 -0.010398)
431:	3248.01	0.001074	5.43	0.000103	(0.007020 0.004441 0.005849)
432:	3248.74	0.001072	5.42	0.000103	(0.005450 0.003996 -0.007567)
433:	3250.81	0.000378	1.91	0.000036	(-0.004651 0.001837 -0.003358)
434:	3251.68	0.000358	1.81	0.000034	(0.004614 -0.001205 -0.003412)
435:	3252.34	0.000710	3.59	0.000068	(-0.007057 0.002656 -0.003354)
436:	3253.45	0.000474	2.39	0.000045	(-0.004614 0.004838 -0.000867)
437:	3253.69	0.001363	6.89	0.000131	(0.008021 -0.008136 -0.000471)
438:	3254.18	0.000509	2.57	0.000049	(-0.005213 0.003269 0.003313)
439:	3255.47	0.000099	0.50	0.000010	(-0.001838 -0.001485 0.001988)
440:	3256.78	0.000137	0.69	0.000013	(0.002374 -0.002071 -0.001794)
441:	3261.20	0.000416	2.10	0.000040	(-0.001760 0.005692 0.002088)
442:	3263.59	0.000610	3.08	0.000058	(0.000874 -0.003545 -0.006708)
443:	3263.63	0.000675	3.41	0.000065	(-0.001782 0.004391 -0.006490)
444:	3264.62	0.001364	6.90	0.000130	(0.003141 0.010975 -0.000327)
445:	3310.34	0.002170	10.97	0.000205	(0.012246 -0.005960 0.004368)
446:	3310.42	0.001884	9.52	0.000178	(0.010772 -0.005614 -0.005478)
447:	3311.49	0.002175	10.99	0.000205	(0.006332 -0.012783 -0.001191)

```
448:   3311.64   0.002260   11.42  0.000213  ( 0.006701 -0.012948  0.000632)
449:   3331.74   0.002865   14.48  0.000268  ( 0.011188  0.005355 -0.010701)
450:   3331.94   0.003185   16.10  0.000298  ( 0.002438  0.009065  0.014498)
451:   3332.02   0.001830    9.25  0.000171  ( 0.012958 -0.000889  0.001639)
452:   3332.18   0.002059   10.41  0.000193  ( 0.006355 -0.006998  0.010172)
```

The following input files have been used to probe the relative energeties of the possible local spin arrangements of **2** yielding information for the discussion based on Table 1 in the main text. In this series of geometry optimizations the authors used the broken symmetry approach along with a truncated model complex shown in Figure S29 below. In thiese calculations basis sets of DKH quality (DKH-def2-SVP for atoms other than Ni and Co) and DKH-def2-TZVP for Ni and Co have been used along the exchange correlation functional wB97X-D4 and D3BJ for non-bonding interactions.

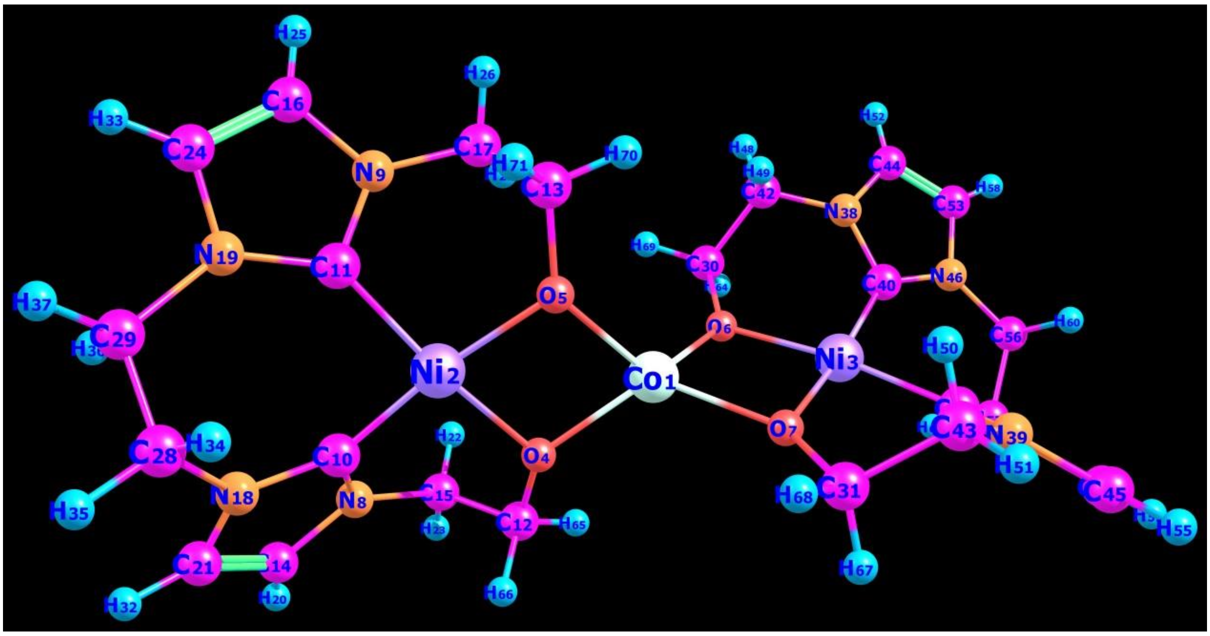

Figure S29. The truncated model complex for the NiCoNi employed in the study of the local spin energetics and the valence formulations of Ni and Co.

Total spin Ms=1/2, spin-distribution on NiCoNi [0,1/2,0].

Input file - single point calculation using the truncated geometry

coni2wb97xtdub.inp:

```
!UKS wB97X-D4 D3BJ DKH2 DKH-def2-SVP Autoaux

%basis
newgto Co "dkh-def2-tzvp" end
newgto Ni "dkh-def2-tzvp" end
end

%pal nprocs 16 end
```

```
%maxcore 8000

%rel method DKH
picturechange 2
end

%scf maxiter 500 shift shift 0.5 erroff 0 end end

*xyz 2 2
27       0.000000000      0.000000000      0.000000000
28       2.786410000     -1.027250000     -0.292530000
28      -2.774910000      1.072990000     -0.099920000
8        1.782730000      0.177430000      0.870950000
8        1.037300000     -1.353830000     -1.022910000
8       -1.068410000      1.412490000     -0.917820000
8       -1.735190000     -0.178420000      0.970180000
7        4.670250000      0.491630000      1.140380000
7        3.235200000     -1.847020000     -2.924570000
6        4.351860000     -0.712490000      0.621040000
6        3.737240000     -1.914630000     -1.653540000
6        2.386410000      1.153410000      1.725940000
6        0.873920000     -2.023450000     -2.274580000
6        5.823960000      0.394750000      1.915330000
6        3.736860000      1.607820000      1.108970000
6        4.109770000     -2.381410000     -3.850190000
6        1.881590000     -1.434810000     -3.260560000
7        5.315390000     -1.553140000      1.048450000
7        4.945970000     -2.513650000     -1.817710000
1        6.235330000      1.094310000      2.407170000
6        6.241010000     -0.872460000      1.833160000
1        3.597950000      1.902700000      0.173590000
1        4.104340000      2.369030000      1.624250000
6        5.189790000     -2.789520000     -3.159200000
1        3.975160000     -2.449460000     -4.787530000
1        1.662670000     -1.738520000     -4.178370000
1        1.822860000     -0.446890000     -3.243160000
6        5.438260000     -2.916790000      0.603130000
6        5.948840000     -2.942640000     -0.828000000
6       -0.972080000      2.066320000     -2.187060000
6       -2.287940000     -1.160260000      1.868130000
1        7.021130000     -1.237820000      2.235190000
1        5.972540000     -3.189680000     -3.520570000
1        4.557370000     -3.365670000      0.651770000
1        6.067620000     -3.405440000      1.190640000
1        6.739400000     -2.349470000     -0.896510000
1        6.241990000     -3.861840000     -1.046850000
7       -3.376440000      1.864030000     -2.694860000
7       -4.591190000     -0.434840000      1.446240000
6       -3.797580000      1.954520000     -1.400400000
6       -4.295900000      0.756360000      0.899560000
6       -2.043320000      1.442440000     -3.100970000
6       -3.693050000     -1.570360000      1.348610000
6       -4.336850000      2.309050000     -3.585340000
6       -5.693330000     -0.321930000      2.293420000
7       -5.031790000      2.519850000     -1.506010000
```

```
7       -5.217490000      1.612030000      1.383590000
1       -1.880480000      1.719420000     -4.036350000
1       -1.981550000      0.455630000     -3.056230000
1       -3.628790000     -1.867110000      0.407370000
1       -4.044050000     -2.321510000      1.887310000
1       -4.273150000      2.319550000     -4.532440000
6       -5.382310000      2.723250000     -2.845540000
6       -6.083530000      0.960310000      2.244440000
1       -6.089870000     -1.018390000      2.804790000
6       -5.944190000      3.012590000     -0.449910000
6       -5.340280000      2.986740000      0.928500000
1       -6.199490000      3.085730000     -3.165910000
1       -6.815070000      1.347660000      2.712850000
1       -6.764220000      2.457320000     -0.448130000
1       -6.211690000      3.941850000     -0.662900000
1       -4.448580000      3.414860000      0.912710000
1       -5.914930000      3.496390000      1.557020000
1       -1.126889523      3.127612018     -2.060200772
1        1.671551347      1.956067705      1.831380575
1        2.620358062      0.766547037      2.706758715
1       -2.356449611     -0.733752024      2.857977150
1       -1.657736681     -2.036199755      1.912552100
1        0.004321006      1.936281793     -2.629926955
1       -0.136460702     -1.880476486     -2.628259815
1        1.047754423     -3.084261839     -2.170369462
*
```

FINAL SINGLE POINT ENERGY   -6106.888548765292

Reading the geometry and the electron density geometry optimization using the following input:

```
!UKS wB97X-D4 D3BJ DKH2 DKH-def2-SVP opt  Autoaux moread
%moinp "coni2wb97xtdub.gbw"

%basis
newgto Co "dkh-def2-tzvp" end
newgto Ni "dkh-def2-tzvp" end
end

%pal nprocs 16 end

%maxcore 8000

%rel method DKH
picturechange 2
end

%scf maxiter 500 shift shift 0.5 erroff 0 end end

*xyz 2 2
27       0.000000000      0.000000000      0.000000000
28       2.786410000     -1.027250000     -0.292530000
28      -2.774910000      1.072990000     -0.099920000
```

8	1.782730000	0.177430000	0.870950000
8	1.037300000	-1.353830000	-1.022910000
8	-1.068410000	1.412490000	-0.917820000
8	-1.735190000	-0.178420000	0.970180000
7	4.670250000	0.491630000	1.140380000
7	3.235200000	-1.847020000	-2.924570000
6	4.351860000	-0.712490000	0.621040000
6	3.737240000	-1.914630000	-1.653540000
6	2.386410000	1.153410000	1.725940000
6	0.873920000	-2.023450000	-2.274580000
6	5.823960000	0.394750000	1.915330000
6	3.736860000	1.607820000	1.108970000
6	4.109770000	-2.381410000	-3.850190000
6	1.881590000	-1.434810000	-3.260560000
7	5.315390000	-1.553140000	1.048450000
7	4.945970000	-2.513650000	-1.817710000
1	6.235330000	1.094310000	2.407170000
6	6.241010000	-0.872460000	1.833160000
1	3.597950000	1.902700000	0.173590000
1	4.104340000	2.369030000	1.624250000
6	5.189790000	-2.789520000	-3.159200000
1	3.975160000	-2.449460000	-4.787530000
1	1.662670000	-1.738520000	-4.178370000
1	1.822860000	-0.446890000	-3.243160000
6	5.438260000	-2.916790000	0.603130000
6	5.948840000	-2.942640000	-0.828000000
6	-0.972080000	2.066320000	-2.187060000
6	-2.287940000	-1.160260000	1.868130000
1	7.021130000	-1.237820000	2.235190000
1	5.972540000	-3.189680000	-3.520570000
1	4.557370000	-3.365670000	0.651770000
1	6.067620000	-3.405440000	1.190640000
1	6.739400000	-2.349470000	-0.896510000
1	6.241990000	-3.861840000	-1.046850000
7	-3.376440000	1.864030000	-2.694860000
7	-4.591190000	-0.434840000	1.446240000
6	-3.797580000	1.954520000	-1.400400000
6	-4.295900000	0.756360000	0.899560000
6	-2.043320000	1.442440000	-3.100970000
6	-3.693050000	-1.570360000	1.348610000
6	-4.336850000	2.309050000	-3.585340000
6	-5.693330000	-0.321930000	2.293420000
7	-5.031790000	2.519850000	-1.506010000
7	-5.217490000	1.612030000	1.383590000
1	-1.880480000	1.719420000	-4.036350000
1	-1.981550000	0.455630000	-3.056230000
1	-3.628790000	-1.867110000	0.407370000
1	-4.044050000	-2.321510000	1.887310000
1	-4.273150000	2.319550000	-4.532440000
6	-5.382310000	2.723250000	-2.845540000
6	-6.083530000	0.960310000	2.244440000
1	-6.089870000	-1.018390000	2.804790000
6	-5.944190000	3.012590000	-0.449910000
6	-5.340280000	2.986740000	0.928500000
1	-6.199490000	3.085730000	-3.165910000
1	-6.815070000	1.347660000	2.712850000

```
1       -6.764220000      2.457320000     -0.448130000
1       -6.211690000      3.941850000     -0.662900000
1       -4.448580000      3.414860000      0.912710000
1       -5.914930000      3.496390000      1.557020000
1       -1.126889523      3.127612018     -2.060200772
1        1.671551347      1.956067705      1.831380575
1        2.620358062      0.766547037      2.706758715
1       -2.356449611     -0.733752024      2.857977150
1       -1.657736681     -2.036199755      1.912552100
1        0.004321006      1.936281793     -2.629926955
1       -0.136460702     -1.880476486     -2.628259815
1        1.047754423     -3.084261839     -2.170369462
*
```

Optimized geometry:

71

Coordinates from ORCA-job coni2wb97xtdubopt

```
  Co  -0.05503045386216     -0.04635197600678     -0.48725674434699
  Ni  2.79111284642286     -0.98749493504058     -0.54297003327092
  Ni  -2.75945563065292      1.04340974790127     -0.32036718477289
  O   1.41436232547936     -0.31275447848346      0.67982610457349
  O   1.25246846679218     -0.80019496947788     -1.68982150863289
  O   -1.32830726834753      0.86950146967271     -1.54083746733226
  O   -1.39912663233610      0.34862149609898      0.80674671808636
  N   4.02254793350294     -0.05411802050641      1.83379959163312
  N   3.62686847959424     -1.43083871509206     -3.24968890512701
  C   4.03377346793159     -0.98109447206430      0.85106578984117
  C   3.95342781231528     -1.59875762368484     -1.93940990624140
  C   1.62526351200347      0.38879973582873      1.87759612492504
  C   1.18485585124910     -1.31205040628261     -2.98405303787363
  C   4.97160143087905     -0.34761598342637      2.80134952001057
  C   3.01801005021467      1.00746033076552      1.90380888602944
  C   4.62020480543887     -1.90800954138168     -4.08485778796405
  C   2.39135671729404     -0.83541344132633     -3.77068156189395
  N   4.98892545107740     -1.86199694276207      1.20694449800238
  N   5.16974170988563     -2.20976257627889     -1.97581349072975
  H   5.12587360403221      0.27457225957201      3.67583421290911
```

C	5.58869430147846	-1.49099889350614	2.40350572702797
H	3.15140004615980	1.68647034173321	1.05224043545300
H	3.18037524837862	1.57293639968235	2.82783020481924
C	5.59366652609659	-2.39946461044054	-3.28366806031733
H	4.54412591515778	-1.86602972710211	-5.16562069410818
H	2.31338652680837	-1.12508908203057	-4.82435734869274
H	2.46499329580822	0.25834657208896	-3.71109999369654
C	5.33914923846507	-3.02433802316632	0.40398454371915
C	6.04874792646667	-2.62814654493398	-0.87808902986137
C	-1.36761755579457	1.25403870684805	-2.88156083169675
C	-1.56807864753727	-0.40667191314928	1.97803091641223
H	6.38580595346351	-2.06607005709191	2.86193788187378
H	6.53895389010381	-2.87322621271413	-3.52398692709573
H	4.43555211746408	-3.60868120925401	0.19466882339290
H	6.01038308010938	-3.65049952813656	1.00060412094499
H	6.77181033494636	-1.82818908052434	-0.67061347852242
H	6.60781241215751	-3.49471159170776	-1.24719696645115
N	-3.81108324543532	1.35889656929574	-2.94814841234285
N	-3.93937807950347	0.10887276309205	2.06753010443607
C	-4.01010824291505	1.63561713491811	-1.63152616758190
C	-3.93690174072086	1.05952207150346	1.11101252719218
C	-2.62986167042781	0.70667119674318	-3.51893080256053
C	-2.97402052311145	-0.98825870893168	2.06648014472158
C	-4.86106498793644	1.80824441083344	-3.72835511470691
C	-4.84040567543891	0.42232170182928	3.07502440120173
N	-5.20557234873060	2.28740725771425	-1.60793081831744
N	-4.83301033095689	1.97692306985193	1.51905511026526
H	-2.64212450387049	0.89607615811835	-4.59773536817575
H	-2.69391625507129	-0.37689240588861	-3.35289548507181
H	-3.17675911472310	-1.64531714770709	1.21163891617867
H	-3.10383854227183	-1.56585334036649	2.98791989479277
H	-4.88547552846134	1.68260131228370	-4.80496648875670

C	-5.74173327926245	2.39335562343071	-2.88428624415512
C	-5.41210709428335	1.60477870319233	2.72615948323762
H	-4.99588013208537	-0.21371951051907	3.93949231753233
C	-5.96591420758530	2.84129041863632	-0.48107531421578
C	-5.14637341844749	3.17160292467185	0.75078326170094
H	-6.69016282158353	2.88323079822464	-3.07536876007481
H	-6.16280660230983	2.20704255716769	3.22604915280942
H	-6.76791644861002	2.13993693106990	-0.21467884492921
H	-6.43362507348185	3.76293592058314	-0.84497351195622
H	-4.21564247355786	3.68638833206568	0.48575739211114
H	-5.72793141057315	3.84194227719558	1.39221936379731
H	-1.35470279629579	2.35242515364917	-2.96829587957950
H	0.87881277414419	1.18966657877366	1.97911417647619
H	1.52022264178648	-0.28853725416461	2.74180177198964
H	-1.39091854219947	0.22608904443274	2.86410889842361
H	-0.84104994716245	-1.23213622580773	2.00282309580645
H	-0.48938190567056	0.87189850128725	-3.41709840399179
H	0.27194009267462	-0.97186817057855	-3.49280058165195
H	1.16097466643146	-2.41516270222021	-2.97770541963055

FINAL SINGLE POINT ENERGY -6107.201948948021 Hartree

Total spin Ms=3/2, spin-distribution on NiCoNi [0,3/2,0].

Single point calculation

```
!UKS wB97X-D4 D3BJ DKH2 DKH-def2-SVP Autoaux

%basis
newgto Co "dkh-def2-tzvp" end
newgto Ni "dkh-def2-tzvp" end
end

%pal nprocs 16 end

%maxcore 8000

%rel method DKH
picturechange 2
```

```
end

%scf maxiter 500 shift shift 0.5 erroff 0 end end

*xyz 2 4
27       0.000000000      0.000000000      0.000000000
28       2.786410000     -1.027250000     -0.292530000
28      -2.774910000      1.072990000     -0.099920000
8        1.782730000      0.177430000      0.870950000
8        1.037300000     -1.353830000     -1.022910000
8       -1.068410000      1.412490000     -0.917820000
8       -1.735190000     -0.178420000      0.970180000
7        4.670250000      0.491630000      1.140380000
7        3.235200000     -1.847020000     -2.924570000
6        4.351860000     -0.712490000      0.621040000
6        3.737240000     -1.914630000     -1.653540000
6        2.386410000      1.153410000      1.725940000
6        0.873920000     -2.023450000     -2.274580000
6        5.823960000      0.394750000      1.915330000
6        3.736860000      1.607820000      1.108970000
6        4.109770000     -2.381410000     -3.850190000
6        1.881590000     -1.434810000     -3.260560000
7        5.315390000     -1.553140000      1.048450000
7        4.945970000     -2.513650000     -1.817710000
1        6.235330000      1.094310000      2.407170000
6        6.241010000     -0.872460000      1.833160000
1        3.597950000      1.902700000      0.173590000
1        4.104340000      2.369030000      1.624250000
6        5.189790000     -2.789520000     -3.159200000
1        3.975160000     -2.449460000     -4.787530000
1        1.662670000     -1.738520000     -4.178370000
1        1.822860000     -0.446890000     -3.243160000
6        5.438260000     -2.916790000      0.603130000
6        5.948840000     -2.942640000     -0.828000000
6       -0.972080000      2.066320000     -2.187060000
6       -2.287940000     -1.160260000      1.868130000
1        7.021130000     -1.237820000      2.235190000
1        5.972540000     -3.189680000     -3.520570000
1        4.557370000     -3.365670000      0.651770000
1        6.067620000     -3.405440000      1.190640000
1        6.739400000     -2.349470000     -0.896510000
1        6.241990000     -3.861840000     -1.046850000
7       -3.376440000      1.864030000     -2.694860000
7       -4.591190000     -0.434840000      1.446240000
6       -3.797580000      1.954520000     -1.400400000
6       -4.295900000      0.756360000      0.899560000
6       -2.043320000      1.442440000     -3.100970000
6       -3.693050000     -1.570360000      1.348610000
6       -4.336850000      2.309050000     -3.585340000
6       -5.693330000     -0.321930000      2.293420000
7       -5.031790000      2.519850000     -1.506010000
7       -5.217490000      1.612030000      1.383590000
1       -1.880480000      1.719420000     -4.036350000
1       -1.981550000      0.455630000     -3.056230000
1       -3.628790000     -1.867110000      0.407370000
1       -4.044050000     -2.321510000      1.887310000
```

```
1       -4.273150000      2.319550000     -4.532440000
6       -5.382310000      2.723250000     -2.845540000
6       -6.083530000      0.960310000      2.244440000
1       -6.089870000     -1.018390000      2.804790000
6       -5.944190000      3.012590000     -0.449910000
6       -5.340280000      2.986740000      0.928500000
1       -6.199490000      3.085730000     -3.165910000
1       -6.815070000      1.347660000      2.712850000
1       -6.764220000      2.457320000     -0.448130000
1       -6.211690000      3.941850000     -0.662900000
1       -4.448580000      3.414860000      0.912710000
1       -5.914930000      3.496390000      1.557020000
1       -1.126889523      3.127612018     -2.060200772
1        1.671551347      1.956067705      1.831380575
1        2.620358062      0.766547037      2.706758715
1       -2.356449611     -0.733752024      2.857977150
1       -1.657736681     -2.036199755      1.912552100
1        0.004321006      1.936281793     -2.629926955
1       -0.136460702     -1.880476486     -2.628259815
1        1.047754423     -3.084261839     -2.170369462
*
```

Using input geometry and electron density geometry optimization:

```
!UKS wB97X-D4 D3BJ DKH2 DKH-def2-SVP opt  Autoaux moread
%moinp "coni2wb97xt.gbw"

#!UKS B3LYP  D3BJ DKH2 DKH-def2-SVP Autoaux

%basis
newgto Co "dkh-def2-tzvp" end
newgto Ni "dkh-def2-tzvp" end
end

%pal nprocs 16 end

%maxcore 8000

%rel method DKH
picturechange 2
end

%scf maxiter 500 shift shift 0.5 erroff 0 end end

*xyz 2 4
27       0.000000000      0.000000000      0.000000000
28       2.786410000     -1.027250000     -0.292530000
28      -2.774910000      1.072990000     -0.099920000
8        1.782730000      0.177430000      0.870950000
8        1.037300000     -1.353830000     -1.022910000
8       -1.068410000      1.412490000     -0.917820000
8       -1.735190000     -0.178420000      0.970180000
7        4.670250000      0.491630000      1.140380000
7        3.235200000     -1.847020000     -2.924570000
6        4.351860000     -0.712490000      0.621040000
```

6	3.737240000	-1.914630000	-1.653540000
6	2.386410000	1.153410000	1.725940000
6	0.873920000	-2.023450000	-2.274580000
6	5.823960000	0.394750000	1.915330000
6	3.736860000	1.607820000	1.108970000
6	4.109770000	-2.381410000	-3.850190000
6	1.881590000	-1.434810000	-3.260560000
7	5.315390000	-1.553140000	1.048450000
7	4.945970000	-2.513650000	-1.817710000
1	6.235330000	1.094310000	2.407170000
6	6.241010000	-0.872460000	1.833160000
1	3.597950000	1.902700000	0.173590000
1	4.104340000	2.369030000	1.624250000
6	5.189790000	-2.789520000	-3.159200000
1	3.975160000	-2.449460000	-4.787530000
1	1.662670000	-1.738520000	-4.178370000
1	1.822860000	-0.446890000	-3.243160000
6	5.438260000	-2.916790000	0.603130000
6	5.948840000	-2.942640000	-0.828000000
6	-0.972080000	2.066320000	-2.187060000
6	-2.287940000	-1.160260000	1.868130000
1	7.021130000	-1.237820000	2.235190000
1	5.972540000	-3.189680000	-3.520570000
1	4.557370000	-3.365670000	0.651770000
1	6.067620000	-3.405440000	1.190640000
1	6.739400000	-2.349470000	-0.896510000
1	6.241990000	-3.861840000	-1.046850000
7	-3.376440000	1.864030000	-2.694860000
7	-4.591190000	-0.434840000	1.446240000
6	-3.797580000	1.954520000	-1.400400000
6	-4.295900000	0.756360000	0.899560000
6	-2.043320000	1.442440000	-3.100970000
6	-3.693050000	-1.570360000	1.348610000
6	-4.336850000	2.309050000	-3.585340000
6	-5.693330000	-0.321930000	2.293420000
7	-5.031790000	2.519850000	-1.506010000
7	-5.217490000	1.612030000	1.383590000
1	-1.880480000	1.719420000	-4.036350000
1	-1.981550000	0.455630000	-3.056230000
1	-3.628790000	-1.867110000	0.407370000
1	-4.044050000	-2.321510000	1.887310000
1	-4.273150000	2.319550000	-4.532440000
6	-5.382310000	2.723250000	-2.845540000
6	-6.083530000	0.960310000	2.244440000
1	-6.089870000	-1.018390000	2.804790000
6	-5.944190000	3.012590000	-0.449910000
6	-5.340280000	2.986740000	0.928500000
1	-6.199490000	3.085730000	-3.165910000
1	-6.815070000	1.347660000	2.712850000
1	-6.764220000	2.457320000	-0.448130000
1	-6.211690000	3.941850000	-0.662900000
1	-4.448580000	3.414860000	0.912710000
1	-5.914930000	3.496390000	1.557020000
1	-1.126889523	3.127612018	-2.060200772
1	1.671551347	1.956067705	1.831380575
1	2.620358062	0.766547037	2.706758715

```
1       -2.356449611     -0.733752024      2.857977150
1       -1.657736681     -2.036199755      1.912552100
1        0.004321006      1.936281793     -2.629926955
1       -0.136460702     -1.880476486     -2.628259815
1        1.047754423     -3.084261839     -2.170369462
*
```

The optimized geometry is:

71

Coordinates from ORCA-job coni2wb97xtopt

```
Co  -0.04274593418960     -0.09538536291624     -0.51313172833008
Ni   2.83838188994890     -0.95419979817498     -0.52455719142323
Ni  -2.87043491109969      0.90963324276880     -0.33449430026512
O    1.65483509946737      0.31547990758183      0.35865833148559
O    1.18485275011567     -1.26133623607309     -1.50858709650877
O   -1.28153722108351      1.11396532532991     -1.43250931171208
O   -1.70008963365875     -0.43550321437356      0.46415879517227
N    4.36118940535006      0.67561650636365      1.24825995573228
N    3.51233085113707     -2.20071664824857     -2.97620859939257
C    4.20027427209924     -0.53403526464716      0.67171582591969
C    3.87854574605374     -2.06107969755577     -1.67490536853375
C    2.01162853889577      1.33602195510028      1.24799348504841
C    1.08184657233921     -1.91006436499881     -2.74049709877890
C    5.38785871219355      0.64508202518907      2.18025007133812
C    3.44649795467551      1.79175711862716      1.00339038783144
C    4.42066688263201     -2.97363171149690     -3.67689956677347
C    2.31228440797367     -1.61875247245820     -3.58374347534818
N    5.12261479712977     -1.33000631022634      1.24938226678705
N    5.03049290188403     -2.77496329090056     -1.57020514278057
H    5.67964232073750      1.51634636854474      2.75633629165105
C    5.87436442438381     -0.62384782451511      2.18009497568808
H    3.56600766785970      2.13407300254901     -0.03207197341607
H    3.71903151469207      2.61226440764277      1.67608365130835
C    5.37875401520721     -3.33710963189476     -2.79181561326956
H    4.30497707144926     -3.20007750463663     -4.73083474392549
```

H	2.21030507708906	-2.04765678703957	-4.58654494122521
H	2.44976570271766	-0.53397922866699	-3.68233623842130
C	5.25872595377731	-2.74612657635443	0.93832173263212
C	5.90709805039574	-2.98300428928781	-0.41202546060069
C	-1.21518379662870	1.76774332447790	-2.66361431411860
C	-2.05231747369892	-1.41879419368528	1.39646040155542
H	6.67018703055352	-1.08015924346765	2.75845975991078
H	6.26627105435799	-3.94760597076869	-2.91636189845054
H	4.27204746008875	-3.22041917543257	0.99196220750331
H	5.88791900817153	-3.19909831112148	1.71149250270118
H	6.79146415393394	-2.34103315609727	-0.51847929669776
H	6.24279388487100	-4.02543475077620	-0.45488939915599
N	-3.63228804988026	2.20655488049240	-2.73615654357032
N	-4.34861927111865	-0.60767961044048	1.57133318049000
C	-3.91736867540182	2.08109959841195	-1.41293113288857
C	-4.16085665531677	0.58475999645824	0.96798590599938
C	-2.51205733378495	1.56121662048687	-3.42739060513836
C	-3.52829770340199	-1.78158908670105	1.26862745329730
C	-4.53735412515674	3.03740759391042	-3.37164641240392
C	-5.29359342821287	-0.50670855459785	2.58143669089638
N	-5.01392253397120	2.86358842079059	-1.22897995830154
N	-4.98453274458050	1.44059270513747	1.60670085260989
H	-2.45021556908331	1.99694830459157	-4.43051614862777
H	-2.71988890248832	0.48782626331041	-3.52667062214422
H	-3.74943336828344	-2.12132213010444	0.24904045718498
H	-3.80030540499439	-2.57888434992899	1.96876199661493
H	-4.48051154275955	3.26245810785264	-4.43068929324842
C	-5.40924260232837	3.45324229020070	-2.42288575660518
C	-5.70070955245475	0.78957628949791	2.60346112104452
H	-5.59152344168413	-1.35407843865814	3.18908868657883
C	-5.79309410331365	3.12306141733026	-0.01299455847262
C	-5.06279612988469	2.85961888726781	1.28959906762350

H	-6.26473428567056	4.11686052415175	-2.48570201952529
H	-6.41990064798232	1.29849084329677	3.23583416138819
H	-6.71620094018351	2.52967737199125	-0.04952079220322
H	-6.07478738804185	4.18183534397762	-0.04107742083585
H	-4.05031148172110	3.27913106250245	1.26694399870625
H	-5.60848557025188	3.35294974008403	2.10064525713991
H	-1.03519534875297	2.84873156458474	-2.53395776997564
H	1.34704183853398	2.20343067947461	1.11219221716501
H	1.91525598251537	0.99798343431413	2.29396727265498
H	-1.85453610904236	-1.07486770408626	2.42617465774193
H	-1.45812188300831	-2.32980169038589	1.22588474604526
H	-0.38739844299907	1.36359416910463	-3.26823090009987
H	0.19422086920060	-1.55545760199952	-3.28827347512336
H	0.97833666468197	-3.00063066167911	-2.61034066115422

FINAL SINGLE POINT ENERGY -6107.227195048673 Hatree

Total spin Ms=5/2, spin-distribution on NiCoNi [0,3/2,1] or [1,3/2,0].

Input file – single point calculation using the truncated geometry

```
coni2wb97xtsext.inp
!UKS wB97X-D4 D3BJ DKH2 DKH-def2-SVP Autoaux

%basis
newgto Co "dkh-def2-tzvp" end
newgto Ni "dkh-def2-tzvp" end
end

%pal nprocs 16 end

%maxcore 8000

%rel method DKH
picturechange 2
end

%scf maxiter 500 shift shift 0.5 erroff 0 end end

*xyz 2 6
27      0.000000000      0.000000000      0.000000000
```

28	2.786410000	-1.027250000	-0.292530000
28	-2.774910000	1.072990000	-0.099920000
8	1.782730000	0.177430000	0.870950000
8	1.037300000	-1.353830000	-1.022910000
8	-1.068410000	1.412490000	-0.917820000
8	-1.735190000	-0.178420000	0.970180000
7	4.670250000	0.491630000	1.140380000
7	3.235200000	-1.847020000	-2.924570000
6	4.351860000	-0.712490000	0.621040000
6	3.737240000	-1.914630000	-1.653540000
6	2.386410000	1.153410000	1.725940000
6	0.873920000	-2.023450000	-2.274580000
6	5.823960000	0.394750000	1.915330000
6	3.736860000	1.607820000	1.108970000
6	4.109770000	-2.381410000	-3.850190000
6	1.881590000	-1.434810000	-3.260560000
7	5.315390000	-1.553140000	1.048450000
7	4.945970000	-2.513650000	-1.817710000
1	6.235330000	1.094310000	2.407170000
6	6.241010000	-0.872460000	1.833160000
1	3.597950000	1.902700000	0.173590000
1	4.104340000	2.369030000	1.624250000
6	5.189790000	-2.789520000	-3.159200000
1	3.975160000	-2.449460000	-4.787530000
1	1.662670000	-1.738520000	-4.178370000
1	1.822860000	-0.446890000	-3.243160000
6	5.438260000	-2.916790000	0.603130000
6	5.948840000	-2.942640000	-0.828000000
6	-0.972080000	2.066320000	-2.187060000
6	-2.287940000	-1.160260000	1.868130000
1	7.021130000	-1.237820000	2.235190000
1	5.972540000	-3.189680000	-3.520570000
1	4.557370000	-3.365670000	0.651770000
1	6.067620000	-3.405440000	1.190640000
1	6.739400000	-2.349470000	-0.896510000
1	6.241990000	-3.861840000	-1.046850000
7	-3.376440000	1.864030000	-2.694860000
7	-4.591190000	-0.434840000	1.446240000
6	-3.797580000	1.954520000	-1.400400000
6	-4.295900000	0.756360000	0.899560000
6	-2.043320000	1.442440000	-3.100970000
6	-3.693050000	-1.570360000	1.348610000
6	-4.336850000	2.309050000	-3.585340000
6	-5.693330000	-0.321930000	2.293420000
7	-5.031790000	2.519850000	-1.506010000
7	-5.217490000	1.612030000	1.383590000
1	-1.880480000	1.719420000	-4.036350000
1	-1.981550000	0.455630000	-3.056230000
1	-3.628790000	-1.867110000	0.407370000
1	-4.044050000	-2.321510000	1.887310000
1	-4.273150000	2.319550000	-4.532440000
6	-5.382310000	2.723250000	-2.845540000
6	-6.083530000	0.960310000	2.244440000
1	-6.089870000	-1.018390000	2.804790000
6	-5.944190000	3.012590000	-0.449910000
6	-5.340280000	2.986740000	0.928500000

```
1       -6.199490000      3.085730000     -3.165910000
1       -6.815070000      1.347660000      2.712850000
1       -6.764220000      2.457320000     -0.448130000
1       -6.211690000      3.941850000     -0.662900000
1       -4.448580000      3.414860000      0.912710000
1       -5.914930000      3.496390000      1.557020000
1       -1.126889523      3.127612018     -2.060200772
1        1.671551347      1.956067705      1.831380575
1        2.620358062      0.766547037      2.706758715
1       -2.356449611     -0.733752024      2.857977150
1       -1.657736681     -2.036199755      1.912552100
1        0.004321006      1.936281793     -2.629926955
1       -0.136460702     -1.880476486     -2.628259815
1        1.047754423     -3.084261839     -2.170369462
*
```

FINAL SINGLE POINT ENERGY   -6106.872390653029

Optimization:

```
!UKS wB97X-D4 D3BJ DKH2 DKH-def2-SVP opt  Autoaux moread
%moinp "coni2wb97xtsext.gbw"

%basis
newgto Co "dkh-def2-tzvp" end
newgto Ni "dkh-def2-tzvp" end
end

%pal nprocs 16 end

%maxcore 8000

%rel method DKH
picturechange 2
end

%scf maxiter 500 shift shift 0.5 erroff 0 end end

*xyz 2 6
27       0.000000000      0.000000000      0.000000000
28       2.786410000     -1.027250000     -0.292530000
28      -2.774910000      1.072990000     -0.099920000
8        1.782730000      0.177430000      0.870950000
8        1.037300000     -1.353830000     -1.022910000
8       -1.068410000      1.412490000     -0.917820000
8       -1.735190000     -0.178420000      0.970180000
7        4.670250000      0.491630000      1.140380000
7        3.235200000     -1.847020000     -2.924570000
6        4.351860000     -0.712490000      0.621040000
6        3.737240000     -1.914630000     -1.653540000
6        2.386410000      1.153410000      1.725940000
6        0.873920000     -2.023450000     -2.274580000
```

6	5.823960000	0.394750000	1.915330000
6	3.736860000	1.607820000	1.108970000
6	4.109770000	-2.381410000	-3.850190000
6	1.881590000	-1.434810000	-3.260560000
7	5.315390000	-1.553140000	1.048450000
7	4.945970000	-2.513650000	-1.817710000
1	6.235330000	1.094310000	2.407170000
6	6.241010000	-0.872460000	1.833160000
1	3.597950000	1.902700000	0.173590000
1	4.104340000	2.369030000	1.624250000
6	5.189790000	-2.789520000	-3.159200000
1	3.975160000	-2.449460000	-4.787530000
1	1.662670000	-1.738520000	-4.178370000
1	1.822860000	-0.446890000	-3.243160000
6	5.438260000	-2.916790000	0.603130000
6	5.948840000	-2.942640000	-0.828000000
6	-0.972080000	2.066320000	-2.187060000
6	-2.287940000	-1.160260000	1.868130000
1	7.021130000	-1.237820000	2.235190000
1	5.972540000	-3.189680000	-3.520570000
1	4.557370000	-3.365670000	0.651770000
1	6.067620000	-3.405440000	1.190640000
1	6.739400000	-2.349470000	-0.896510000
1	6.241990000	-3.861840000	-1.046850000
7	-3.376440000	1.864030000	-2.694860000
7	-4.591190000	-0.434840000	1.446240000
6	-3.797580000	1.954520000	-1.400400000
6	-4.295900000	0.756360000	0.899560000
6	-2.043320000	1.442440000	-3.100970000
6	-3.693050000	-1.570360000	1.348610000
6	-4.336850000	2.309050000	-3.585340000
6	-5.693330000	-0.321930000	2.293420000
7	-5.031790000	2.519850000	-1.506010000
7	-5.217490000	1.612030000	1.383590000
1	-1.880480000	1.719420000	-4.036350000
1	-1.981550000	0.455630000	-3.056230000
1	-3.628790000	-1.867110000	0.407370000
1	-4.044050000	-2.321510000	1.887310000
1	-4.273150000	2.319550000	-4.532440000
6	-5.382310000	2.723250000	-2.845540000
6	-6.083530000	0.960310000	2.244440000
1	-6.089870000	-1.018390000	2.804790000
6	-5.944190000	3.012590000	-0.449910000
6	-5.340280000	2.986740000	0.928500000
1	-6.199490000	3.085730000	-3.165910000
1	-6.815070000	1.347660000	2.712850000
1	-6.764220000	2.457320000	-0.448130000
1	-6.211690000	3.941850000	-0.662900000
1	-4.448580000	3.414860000	0.912710000
1	-5.914930000	3.496390000	1.557020000
1	-1.126889523	3.127612018	-2.060200772
1	1.671551347	1.956067705	1.831380575
1	2.620358062	0.766547037	2.706758715
1	-2.356449611	-0.733752024	2.857977150
1	-1.657736681	-2.036199755	1.912552100
1	0.004321006	1.936281793	-2.629926955

```
1        -0.136460702     -1.880476486     -2.628259815
1         1.047754423     -3.084261839     -2.170369462
*
```

Optimized geometry:

```
71
Coordinates from ORCA-job coni2wb97xtsextopt
  Co  -0.06594603018430     -0.06780395277610     -0.48605092967489
  Ni  2.80649831665552     -0.96140757181674     -0.56328004844995
  Ni  -2.91507041303596      0.71881765408807     -0.39828222493925
  O   1.58696864553522      0.07215652177971      0.56315154964101
  O   1.21273253840683     -0.96732793356016     -1.65990757831842
  O   -1.25460332527340      1.24179678425903     -1.30554995405252
  O   -1.68309357215537     -0.57334324934850      0.47816979879120
  N   4.25503865834919      0.17870175872881      1.60925080506211
  N   3.54421075469122     -1.70961240530506     -3.20997730161737
  C   4.11358981338665     -0.86111792700601      0.76003839823000
  C   3.87174402124833     -1.82358125890068     -1.89519386516004
  C   1.91540212507488      0.86350456022273      1.66898669876030
  C   1.11968498858813     -1.39429440849380     -2.98442086026232
  C   5.22763090141501     -0.08947874797512      2.56107564237214
  C   3.36284154364707      1.33820199851311      1.58857815221930
  C   4.45679068010864     -2.36254841570723     -4.01872408724920
  C   2.37681261447966     -1.00026062375436     -3.73893509857402
  N   4.99394085974371     -1.78945311375889      1.18580660312011
  N   5.00708081240896     -2.57270058364649     -1.89796275329591
  H   5.49720145294005      0.61963041177707      3.33590485916122
  C   5.69995475117434     -1.33489361389183      2.29231139809477
  H   3.52737548750099      1.90160232308369      0.66167885676681
  H   3.61655639107410      1.98125175374864      2.43818421955577
  C   5.38086598397481     -2.90676995489589     -3.19280494525575
  H   4.36617146826582     -2.38872908644153     -5.09883685437741
  H   2.29280018648177     -1.25426341739148     -4.80120621980536
  H   2.54244813663029      0.08149351123695     -3.65128072211580
  C   5.14135390564348     -3.09571188611851      0.56005905663025
  C   5.84777887956221     -3.01244121620875     -0.77912305357875
  C   -1.14504410607909      1.96560391208227     -2.49957584203130
  C   -1.68393612142656     -0.84188912371317      1.85392606492438
  H   6.45900284151094     -1.93012272885184      2.78797564715509
  H   6.25898370509823     -3.50637831665589     -3.40551044254017
  H   4.15357177108052     -3.56000387354040      0.45814153235384
  H   5.73575471613237     -3.72247545121801      1.23288029982011
  H   6.71600473930464     -2.34476173210744     -0.70093971206482
  H   6.21557028457479     -4.01128536822519     -1.03941331998131
  N   -3.56251939520437      1.92114931665733     -3.03280975682270
  N   -3.98222645197141     -0.00331081095694      2.29677956019930
  C   -4.04353498898694      1.61715335344410     -1.80564314199093
  C   -4.05311979610320      0.84931521908594      1.25147463993444
  C   -2.22221730456363      1.55810834555353     -3.49980756306573
  C   -3.07979208444980     -1.15974337784652      2.36730059893378
  C   -4.48299206243145      2.64842895726514     -3.76940272514290
  C   -4.94350002134748      0.29496315517754      3.24916747393589
  N   -5.27578688341032      2.17662054834562     -1.77208144098732
  N   -5.06947837144720      1.68753681932106      1.55316423457099
  H   -2.05909445057818      2.06167318403881     -4.45928840594649
  H   -2.18569360778514      0.47413865065582     -3.67204150956077
  H   -3.51293929023861     -1.98233024340651      1.78335468880398
  H   -3.02747744698621     -1.47273827946119      3.41620488496576
  H   -4.29119261658953      2.98494895411094     -4.78228816169798
  C   -5.56915153104739      2.81150660107917     -2.97075485589520
```

```
  C   -5.63281169940092      1.36825348963351      2.77939022817398
  H   -5.05436363386602     -0.27144479279670      4.16738815834292
  C   -6.18864304933784      2.16103987024211     -0.62786582007290
  C   -5.57250486378096      2.72754103635567      0.65888962863513
  H   -6.51127862193464      3.31778267190387     -3.15050719748620
  H   -6.45744583397240      1.92535435372106      3.21078488218280
  H   -6.54834363193200      1.13925335995272     -0.45682884319116
  H   -7.05388297519136      2.76800076139353     -0.91308260132813
  H   -4.76401701062164      3.42775835365019      0.41971660202688
  H   -6.33432666333599      3.28357599395239      1.21467915729121
  H   -1.22167324709482      3.04737635683836     -2.30355981335248
  H   1.26540043324653      1.75208445358128      1.70697517852435
  H   1.77479469548498      0.30316746430099      2.60967621638193
  H   -1.28142912065288      0.01088695053166      2.42987738489878
  H   -1.04595577439288     -1.71336041714182      2.07005204487918
  H   -0.16336821683128      1.78639495221484     -2.96682419838516
  H   0.25588997196403     -0.92069150025131     -3.47855334443752
  H   0.97992545925720     -2.48692053035727     -3.04465841663147

FINAL SINGLE POINT ENERGY     -6107.198982016148
```

Total spin Ms=7/2, spin-distribution on NiCoNi [1,3/2,1].

Input file – single point calculation using the truncated geometry

coni2wb97xtoct.inp:

```
!UKS wB97X-D4 D3BJ DKH2 DKH-def2-SVP Autoaux

%basis
newgto Co "dkh-def2-tzvp" end
newgto Ni "dkh-def2-tzvp" end
end

%pal nprocs 16 end

%maxcore 8000

%rel method DKH
picturechange 2
end

%scf maxiter 500 shift shift 0.5 erroff 0 end end

*xyz 2 8
27       0.000000000      0.000000000      0.000000000
28       2.786410000     -1.027250000     -0.292530000
28      -2.774910000      1.072990000     -0.099920000
8        1.782730000      0.177430000      0.870950000
8        1.037300000     -1.353830000     -1.022910000
8       -1.068410000      1.412490000     -0.917820000
8       -1.735190000     -0.178420000      0.970180000
7        4.670250000      0.491630000      1.140380000
7        3.235200000     -1.847020000     -2.924570000
6        4.351860000     -0.712490000      0.621040000
6        3.737240000     -1.914630000     -1.653540000
```

6	2.386410000	1.153410000	1.725940000
6	0.873920000	-2.023450000	-2.274580000
6	5.823960000	0.394750000	1.915330000
6	3.736860000	1.607820000	1.108970000
6	4.109770000	-2.381410000	-3.850190000
6	1.881590000	-1.434810000	-3.260560000
7	5.315390000	-1.553140000	1.048450000
7	4.945970000	-2.513650000	-1.817710000
1	6.235330000	1.094310000	2.407170000
6	6.241010000	-0.872460000	1.833160000
1	3.597950000	1.902700000	0.173590000
1	4.104340000	2.369030000	1.624250000
6	5.189790000	-2.789520000	-3.159200000
1	3.975160000	-2.449460000	-4.787530000
1	1.662670000	-1.738520000	-4.178370000
1	1.822860000	-0.446890000	-3.243160000
6	5.438260000	-2.916790000	0.603130000
6	5.948840000	-2.942640000	-0.828000000
6	-0.972080000	2.066320000	-2.187060000
6	-2.287940000	-1.160260000	1.868130000
1	7.021130000	-1.237820000	2.235190000
1	5.972540000	-3.189680000	-3.520570000
1	4.557370000	-3.365670000	0.651770000
1	6.067620000	-3.405440000	1.190640000
1	6.739400000	-2.349470000	-0.896510000
1	6.241990000	-3.861840000	-1.046850000
7	-3.376440000	1.864030000	-2.694860000
7	-4.591190000	-0.434840000	1.446240000
6	-3.797580000	1.954520000	-1.400400000
6	-4.295900000	0.756360000	0.899560000
6	-2.043320000	1.442440000	-3.100970000
6	-3.693050000	-1.570360000	1.348610000
6	-4.336850000	2.309050000	-3.585340000
6	-5.693330000	-0.321930000	2.293420000
7	-5.031790000	2.519850000	-1.506010000
7	-5.217490000	1.612030000	1.383590000
1	-1.880480000	1.719420000	-4.036350000
1	-1.981550000	0.455630000	-3.056230000
1	-3.628790000	-1.867110000	0.407370000
1	-4.044050000	-2.321510000	1.887310000
1	-4.273150000	2.319550000	-4.532440000
6	-5.382310000	2.723250000	-2.845540000
6	-6.083530000	0.960310000	2.244440000
1	-6.089870000	-1.018390000	2.804790000
6	-5.944190000	3.012590000	-0.449910000
6	-5.340280000	2.986740000	0.928500000
1	-6.199490000	3.085730000	-3.165910000
1	-6.815070000	1.347660000	2.712850000
1	-6.764220000	2.457320000	-0.448130000
1	-6.211690000	3.941850000	-0.662900000
1	-4.448580000	3.414860000	0.912710000
1	-5.914930000	3.496390000	1.557020000
1	-1.126889523	3.127612018	-2.060200772
1	1.671551347	1.956067705	1.831380575
1	2.620358062	0.766547037	2.706758715
1	-2.356449611	-0.733752024	2.857977150

```
1       -1.657736681     -2.036199755      1.912552100
1        0.004321006      1.936281793     -2.629926955
1       -0.136460702     -1.880476486     -2.628259815
1        1.047754423     -3.084261839     -2.170369462
*

FINAL SINGLE POINT ENERGY     -6106.815677234925
```

Geometry optimization:

```
!UKS wB97X-D4 D3BJ DKH2 DKH-def2-SVP opt  Autoaux moread
%moinp "coni2wb97xtoct.gbw"


%basis
newgto Co "dkh-def2-tzvp" end
newgto Ni "dkh-def2-tzvp" end
end

%pal nprocs 16 end

%maxcore 8000

%rel method DKH
picturechange 2
end

%scf maxiter 500 shift shift 0.5 erroff 0 end end

*xyz 2 8
27       0.000000000      0.000000000      0.000000000
28       2.786410000     -1.027250000     -0.292530000
28      -2.774910000      1.072990000     -0.099920000
8        1.782730000      0.177430000      0.870950000
8        1.037300000     -1.353830000     -1.022910000
8       -1.068410000      1.412490000     -0.917820000
8       -1.735190000     -0.178420000      0.970180000
7        4.670250000      0.491630000      1.140380000
7        3.235200000     -1.847020000     -2.924570000
6        4.351860000     -0.712490000      0.621040000
6        3.737240000     -1.914630000     -1.653540000
6        2.386410000      1.153410000      1.725940000
6        0.873920000     -2.023450000     -2.274580000
6        5.823960000      0.394750000      1.915330000
6        3.736860000      1.607820000      1.108970000
6        4.109770000     -2.381410000     -3.850190000
6        1.881590000     -1.434810000     -3.260560000
7        5.315390000     -1.553140000      1.048450000
7        4.945970000     -2.513650000     -1.817710000
1        6.235330000      1.094310000      2.407170000
6        6.241010000     -0.872460000      1.833160000
1        3.597950000      1.902700000      0.173590000
1        4.104340000      2.369030000      1.624250000
6        5.189790000     -2.789520000     -3.159200000
1        3.975160000     -2.449460000     -4.787530000
```

```
1        1.662670000     -1.738520000     -4.178370000
1        1.822860000     -0.446890000     -3.243160000
6        5.438260000     -2.916790000      0.603130000
6        5.948840000     -2.942640000     -0.828000000
6       -0.972080000      2.066320000     -2.187060000
6       -2.287940000     -1.160260000      1.868130000
1        7.021130000     -1.237820000      2.235190000
1        5.972540000     -3.189680000     -3.520570000
1        4.557370000     -3.365670000      0.651770000
1        6.067620000     -3.405440000      1.190640000
1        6.739400000     -2.349470000     -0.896510000
1        6.241990000     -3.861840000     -1.046850000
7       -3.376440000      1.864030000     -2.694860000
7       -4.591190000     -0.434840000      1.446240000
6       -3.797580000      1.954520000     -1.400400000
6       -4.295900000      0.756360000      0.899560000
6       -2.043320000      1.442440000     -3.100970000
6       -3.693050000     -1.570360000      1.348610000
6       -4.336850000      2.309050000     -3.585340000
6       -5.693330000     -0.321930000      2.293420000
7       -5.031790000      2.519850000     -1.506010000
7       -5.217490000      1.612030000      1.383590000
1       -1.880480000      1.719420000     -4.036350000
1       -1.981550000      0.455630000     -3.056230000
1       -3.628790000     -1.867110000      0.407370000
1       -4.044050000     -2.321510000      1.887310000
1       -4.273150000      2.319550000     -4.532440000
6       -5.382310000      2.723250000     -2.845540000
6       -6.083530000      0.960310000      2.244440000
1       -6.089870000     -1.018390000      2.804790000
6       -5.944190000      3.012590000     -0.449910000
6       -5.340280000      2.986740000      0.928500000
1       -6.199490000      3.085730000     -3.165910000
1       -6.815070000      1.347660000      2.712850000
1       -6.764220000      2.457320000     -0.448130000
1       -6.211690000      3.941850000     -0.662900000
1       -4.448580000      3.414860000      0.912710000
1       -5.914930000      3.496390000      1.557020000
1       -1.126889523      3.127612018     -2.060200772
1        1.671551347      1.956067705      1.831380575
1        2.620358062      0.766547037      2.706758715
1       -2.356449611     -0.733752024      2.857977150
1       -1.657736681     -2.036199755      1.912552100
1        0.004321006      1.936281793     -2.629926955
1       -0.136460702     -1.880476486     -2.628259815
1        1.047754423     -3.084261839     -2.170369462
*
```

Optimized geometry:

```
71
Coordinates from ORCA-job coni2wb97xtoctopt
  Co  -0.04976021947411     -0.07857932207027     -0.67810549130494
  Ni  2.84379256691407     -0.78291399876491     -0.67157284532769
  Ni  -2.90737915034363      0.73871827633650     -0.51449303753913
```

O	1.59658400014810	0.51047220758557	0.19518689822067
O	1.16951349735032	-1.33070197535109	-1.53377439346752
O	-1.28282951404944	1.17395079526866	-1.51899887566368
O	-1.66477175861565	-0.61098323717397	0.28088740260374
N	4.02997966744855	0.20023756527495	1.89006190175638
N	3.44122983589197	-2.14727836035752	-3.24966534644575
C	4.07227276812415	-0.73726439009792	0.91902757077250
C	3.94731302556540	-1.76322456642029	-2.05492659788548
C	1.68636287797023	0.93542242942114	1.52437621050478
C	1.03745101080994	-2.15978677396080	-2.65521522567841
C	5.02825571561164	-0.00849116838983	2.82825546876534
C	3.10519722158739	1.34013890252913	1.89763773975492
C	4.35617140859625	-2.89821758388644	-3.96827734956693
C	2.08690273515258	-1.83034143680339	-3.71058056436778
N	5.10696745130072	-1.53889164832158	1.25284115012440
N	5.19221990725415	-2.29627497792768	-2.02483692608432
H	5.16625792965463	0.63360139463183	3.69108133250420
C	5.71173227741550	-1.11247599232854	2.42565936415582
H	3.47164877733118	2.09950565611637	1.19431780491838
H	3.11974760938527	1.76958225624377	2.90565103529958
C	5.46645032438194	-2.99383508752518	-3.19237519259961
H	4.14369322490683	-3.29612314962830	-4.95447836319335
H	1.90465909438529	-2.41040988309002	-4.62220302614580
H	2.03714297687779	-0.76404716789203	-3.96788995421209
C	5.58370628870055	-2.64823534530693	0.43103394218950
C	6.14575721502907	-2.18575304210130	-0.91951142992288
C	-1.18890487310981	1.97490907805502	-2.66385694801999
C	-1.68885987354715	-1.05248373593664	1.60748876153158
H	6.55727410284082	-1.62532021437743	2.87150407632664
H	6.41288890738479	-3.49330349326401	-3.36875827003896
H	4.77277724460721	-3.37011889612673	0.28163393307551
H	6.37045720162372	-3.15232796483931	1.00157077490237
H	6.50367768092952	-1.15180895920903	-0.84402216181878
H	7.00470517167761	-2.80870078154451	-1.18783751923853
N	-3.62852526304406	2.12762110372208	-3.04677860842405
N	-3.93176173546842	-0.13620071629761	2.15096681408954
C	-4.05630524930810	1.78909496718191	-1.80856392515160
C	-3.98248612852399	0.80106549762708	1.18013307901971
C	-2.34676641790609	1.70559564772903	-3.61792444594516
C	-3.10313466612299	-1.34600674582640	2.08696611524696
C	-4.53927980745302	2.94894124789742	-3.68962109428323
C	-4.83026977897329	0.14908517948604	3.16653915716413
N	-5.24611493548728	2.42248179017640	-1.67415968884666
N	-4.92127998436582	1.68060379772186	1.59232122153777
H	-2.20459216571952	2.25640296218899	-4.55442416249077
H	-2.39614376630087	0.63473316185872	-3.85493303584322
H	-3.58333131673866	-2.06968745462525	1.41540129099354
H	-3.07397283589422	-1.77921868159185	3.09295461073716
H	-4.38302604138532	3.32597512651248	-4.69427118753060
C	-5.56513083723965	3.13692318945423	-2.82001126705685
C	-5.45812370876396	1.30215376698478	2.81380275362858
H	-4.94601841906705	-0.48200291912276	4.04088317287323
C	-6.10060985589210	2.39656600743347	-0.48574634466826
C	-5.38105851120725	2.81984768241458	0.80204033872456
H	-6.48037882277592	3.71055192919536	-2.91803981626559
H	-6.22305881865417	1.87799057514251	3.32347695321666

H	-6.53308925066092	1.39598948740393	-0.36460815420868
H	-6.92715135898878	3.08613712039300	-0.68366973982838
H	-4.53090515992560	3.47095323056924	0.56920029571757
H	-6.06793217421181	3.39083661275086	1.43524056692746
H	-1.18007155712453	3.04272196215607	-2.39112568212669
H	1.04102863288482	1.81429324129791	1.68716344296842
H	1.35164921343233	0.14582767290014	2.22182957303517
H	-1.23677671990298	-0.30715568509284	2.28684980401700
H	-1.10857056933760	-1.98408982358631	1.70914207157232
H	-0.25025527522025	1.76770003414135	-3.20149012637336
H	0.04378331969508	-2.03156769111258	-3.11246937435036
H	1.12929395893465	-3.21923023685114	-2.36588892096093

FINAL SINGLE POINT ENERGY -6107.169355108423

Unsing final energies we got the following list:

spin dublet (M_s=1/2) single point
-6106.888548765292 Hartree
spin dublet DFT geometry optimized
FINAL SINGLE POINT ENERGY -6107.201948948021

spin quartet (M_s=3/2) single point
FINAL SINGLE POINT ENERGY -6106.928243568225
spin quartet optimized
FINAL SINGLE POINT ENERGY -6107.227195048673

spin sextet (M_s=5/2) single point
FINAL SINGLE POINT ENERGY -6106.872390653029
spin sextet optimized
FINAL SINGLE POINT ENERGY -6107.198982016148

spin octet (M_s=7/2) single point
FINAL SINGLE POINT ENERGY -6106.815677234925
spin octate optimized geometry
FINAL SINGLE POINT ENERGY -6107.169355108423

Comparing the energy stabilizations between optimized and single point energy for each spin we get the geometric response to the change of spin: -6878, -6561, -7168, -7762 cm^{-1} in this order.

The stabilizations of the spin-arrangements resulting in the M_s=1/2, M_s=5/2 and M_s=7/2 values of the total spin with respect to the M_s=3/2 one results in 5541, 6192 and 12694 cm^{-1}, respectively which are 15.84, 17.70 and 36.29 in kcal/mol and are listed in Table 1 of the main text.

7. References

1. Llunell, M.; Casanova, D.; Cirera, J.; Bofill, J.; Alemany, P.; Alvarez, S., SHAPE program, version 2.1. *SHAPE program, version 2.1, Universitat de Barcelona: Barcelona, Spain* **2013**.
2. Zadrozny, J. M.; Telser, J.; Long, J. R., Slow magnetic relaxation in the tetrahedral cobalt(II) complexes [Co(EPh)4]2− (EO, S, Se). *Polyhedron* **2013,** *64*, 209-217.
3. Das, M.; Basak, D.; Trávníček, Z.; Vančo, J.; Ray, D., Entrapment of a Pseudo-Tetrahedral CoII Center by Thioether Sulfur Bound {Co2(μ-L)} Fragments: Synthesis, Field-Induced Single-Ion Magnetism and Catechol Oxidase Mimicking Activity. *Chem. Asian J.* **2019,** *14* (21), 3898-3914.
4. Chattopadhyay, K.; Heras Ojea, M. J.; Sarkar, A.; Murrie, M.; Rajaraman, G.; Ray, D., Trapping of a Pseudotetrahedral CoIIO4 Core in Mixed-Valence Mixed-Geometry [Co5] Coordination Aggregates: Synthetic Marvel, Structures, and Magnetism. *Inorg. Chem.* **2018,** *57* (21), 13176-13187.
5. Gupta, S. K.; Nielsen, H. H.; Thiel, A. M.; Klahn, E. A.; Feng, E.; Cao, H. B.; Hansen, T. C.; Lelièvre-Berna, E.; Gukasov, A.; Kibalin, I.; Dechert, S.; Demeshko, S.; Overgaard, J.; Meyer, F., Multi-Technique Experimental Benchmarking of the Local Magnetic Anisotropy of a Cobalt(II) Single-Ion Magnet. *JACS Au* **2023,** *3* (2), 429-440.
6. Rechkemmer, Y.; Breitgoff, F. D.; van der Meer, M.; Atanasov, M.; Hakl, M.; Orlita, M.; Neugebauer, P.; Neese, F.; Sarkar, B.; van Slageren, J., A four-coordinate cobalt(II) single-ion magnet with coercivity and a very high energy barrier. *Nat. Commun.* **2016,** *7* (1), 10467.
7. Bamberger, H.; Albold, U.; Dubnická Midlíková, J.; Su, C.-Y.; Deibel, N.; Hunger, D.; Hallmen, P. P.; Neugebauer, P.; Beerhues, J.; Demeshko, S.; Meyer, F.; Sarkar, B.; van Slageren, J., Iron(II), Cobalt(II), and Nickel(II) Complexes of Bis(sulfonamido)benzenes: Redox Properties, Large Zero-Field Splittings, and Single-Ion Magnets. *Inorg. Chem.* **2021,** *60* (5), 2953-2963.
8. Carl, E.; Demeshko, S.; Meyer, F.; Stalke, D., Triimidosulfonates as Acute Bite-Angle Chelates: Slow Relaxation of the Magnetization in Zero Field and Hysteresis Loop of a CoII Complex. *Chem. Eur. J.* **2015,** *21* (28), 10109-10115.
9. Legendre, C. M.; Damgaard-Møller, E.; Overgaard, J.; Stalke, D., The Quest for Optimal 3 d Orbital Splitting in Tetrahedral Cobalt Single-Molecule Magnets Featuring Colossal Anisotropy and Hysteresis. *Eur. J. Inorg. Chem.* **2021,** *2021* (30), 3108-3114.
10. Cui, H.-H.; Lu, F.; Chen, X.-T.; Zhang, Y.-Q.; Tong, W.; Xue, Z.-L., Zero-Field Slow Magnetic Relaxation and Hysteresis Loop in Four-Coordinate CoII Single-Ion Magnets with Strong Easy-Axis Anisotropy. *Inorg. Chem.* **2019,** *58* (19), 12555-12564.
11. Wang, M.; Xu, H. J.; Sun, T. M.; Cui, H. H.; Zhang, Y.-Q.; Chen, L.; Tang, Y. F., Optimal N–Co–N bite angle for enhancing the magnetic anisotropy of zero-field Co(II) single-ion magnets in tetrahedral [N4] coordination environment. *J. Solid State Chem.* **2021,** *299*, 122209.
12. Wu, T.; Zhai, Y.-Q.; Deng, Y.-F.; Chen, W.-P.; Zhang, T.; Zheng, Y.-Z., Correlating magnetic anisotropy with the subtle coordination geometry variation of a series of cobalt(ii)-sulfonamide complexes. *Dalton Trans.* **2019,** *48* (41), 15419-15426.
13. Ishizaki, T.; Fukuda, T.; Akaki, M.; Fuyuhiro, A.; Hagiwara, M.; Ishikawa, N., Synthesis of a Neutral Mononuclear Four-Coordinate Co(II) Complex Having Two Halved Phthalocyanine Ligands That Shows Slow Magnetic Relaxations under Zero Static Magnetic Field. *Inorg. Chem.* **2019,** *58* (8), 5211-5220.
14. Wu, C.-M.; Tsai, J.-E.; Lee, G.-H.; Yang, E.-C., Slow magnetization relaxation in a tetrahedrally coordinated mononuclear Co(ii) complex exclusively ligated with phenanthroline ligands. *Dalton Trans.* **2020,** *49* (46), 16813-16820.
15. Fataftah, M. S.; Coste, S. C.; Vlaisavljevich, B.; Zadrozny, J. M.; Freedman, D. E., Transformation of the coordination complex [Co(C3S5)2]2− from a molecular magnet to a potential qubit. *Chem. Sci.* **2016,** *7* (9), 6160-6166.
16. Fataftah, M. S.; Zadrozny, J. M.; Rogers, D. M.; Freedman, D. E., A Mononuclear Transition Metal Single-Molecule Magnet in a Nuclear Spin-Free Ligand Environment. *Inorg. Chem.* **2014,** *53* (19), 10716-10721.
17. Tu, D.; Shao, D.; Yan, H.; Lu, C., A carborane-incorporated mononuclear Co(ii) complex showing zero-field slow magnetic relaxation. *Chem. Commun.* **2016,** *52* (99), 14326-14329.
18. Zadrozny, J. M.; Long, J. R., Slow Magnetic Relaxation at Zero Field in the Tetrahedral Complex [Co(SPh)4]2–. *J. Am. Chem. Soc.* **2011,** *133* (51), 20732-20734.
19. Suturina, E. A.; Nehrkorn, J.; Zadrozny, J. M.; Liu, J.; Atanasov, M.; Weyhermüller, T.; Maganas, D.; Hill, S.; Schnegg, A.; Bill, E.; Long, J. R.; Neese, F., Magneto-Structural Correlations in Pseudotetrahedral Forms of the [Co(SPh)4]2– Complex Probed by Magnetometry, MCD Spectroscopy, Advanced EPR Techniques, and ab Initio Electronic Structure Calculations. *Inorg. Chem.* **2017,** *56* (5), 3102-3118.
20. Vaidya, S.; Tewary, S.; Singh, S. K.; Langley, S. K.; Murray, K. S.; Lan, Y.; Wernsdorfer, W.; Rajaraman, G.; Shanmugam, M., What Controls the Sign and Magnitude of Magnetic Anisotropy in Tetrahedral Cobalt(II) Single-Ion Magnets? *Inorg. Chem.* **2016,** *55* (19), 9564-9578.
21. Tripathi, S.; Vaidya, S.; Ansari, K. U.; Ahmed, N.; Rivière, E.; Spillecke, L.; Koo, C.; Klingeler, R.; Mallah, T.; Rajaraman, G.; Shanmugam, M., Influence of a Counteranion on the Zero-Field Splitting of Tetrahedral Cobalt(II) Thiourea Complexes. *Inorg. Chem.* **2019,** *58* (14), 9085-9100.
22. Yao, X.-N.; Yang, M.-W.; Xiong, J.; Liu, J.-J.; Gao, C.; Meng, Y.-S.; Jiang, S.-D.; Wang, B.-W.; Gao, S., Enhanced magnetic anisotropy in a tellurium-coordinated cobalt single-ion magnet. *Inorg. Chem. Front.* **2017,** *4* (4), 701-705.

23. Bunting, P. C.; Atanasov, M.; Damgaard-Møller, E.; Perfetti, M.; Crassee, I.; Orlita, M.; Overgaard, J.; Slageren, J. v.; Neese, F.; Long, J. R., A linear cobalt(II) complex with maximal orbital angular momentum from a non-Aufbau ground state. *Science* **2018,** *362* (6421), eaat7319.
24. Yao, X.-N.; Du, J.-Z.; Zhang, Y.-Q.; Leng, X.-B.; Yang, M.-W.; Jiang, S.-D.; Wang, Z.-X.; Ouyang, Z.-W.; Deng, L.; Wang, B.-W.; Gao, S., Two-Coordinate Co(II) Imido Complexes as Outstanding Single-Molecule Magnets. *J. Am. Chem. Soc.* **2017,** *139* (1), 373-380.
25. Zadrozny, J. M.; Atanasov, M.; Bryan, A. M.; Lin, C.-Y.; Rekken, B. D.; Power, P. P.; Neese, F.; Long, J. R., Slow magnetization dynamics in a series of two-coordinate iron(ii) complexes. *Chem. Sci.* **2013,** *4* (1), 125-138.
26. Zadrozny, J. M.; Xiao, D. J.; Atanasov, M.; Long, G. J.; Grandjean, F.; Neese, F.; Long, J. R., Magnetic blocking in a linear iron(I) complex. *Nat. Chem.* **2013,** *5* (7), 577-581.
27. Deng, Y.-F.; Han, T.; Yin, B.; Zheng, Y.-Z., On balancing the QTM and the direct relaxation processes in single-ion magnets – the importance of symmetry control. *Inorganic Chemistry Frontiers* **2017,** *4* (7), 1141-1148.
28. Gupta, S. K.; Rao, S. V.; Demeshko, S.; Dechert, S.; Bill, E.; Atanasov, M.; Neese, F.; Meyer, F., Air-stable four-coordinate cobalt(ii) single-ion magnets: experimental and ab initio ligand field analyses of correlations between dihedral angles and magnetic anisotropy. *Chem. Sci.* **2023,** *14* (23), 6355-6374.
29. Freedman, D. E.; Harman, W. H.; Harris, T. D.; Long, G. J.; Chang, C. J.; Long, J. R., Slow Magnetic Relaxation in a High-Spin Iron(II) Complex. *J. Am. Chem. Soc.* **2010,** *132* (4), 1224-1225.
30. Feng, X.; Hwang, S. J.; Liu, J.-L.; Chen, Y.-C.; Tong, M.-L.; Nocera, D. G., Slow Magnetic Relaxation in Intermediate Spin S = 3/2 Mononuclear Fe(III) Complexes. *J. Am. Chem. Soc.* **2017,** *139* (46), 16474-16477.
31. Yao, B.; Singh, M. K.; Deng, Y.-F.; Wang, Y.-N.; Dunbar, K. R.; Zhang, Y.-Z., Trigonal Prismatic Cobalt(II) Single-Ion Magnets: Manipulating the Magnetic Relaxation Through Symmetry Control. *Inorg. Chem.* **2020,** *59* (12), 8505-8513.
32. Landart-Gereka, A.; Quesada-Moreno, M. M.; Díaz-Ortega, I. F.; Nojiri, H.; Ozerov, M.; Krzystek, J.; Palacios, M. A.; Colacio, E., Large easy-axis magnetic anisotropy in a series of trigonal prismatic mononuclear cobalt(ii) complexes with zero-field hidden single-molecule magnet behaviour: the important role of the distortion of the coordination sphere and intermolecular interactions in the slow relaxation. *Inorganic Chemistry Frontiers* **2022,** *9* (12), 2810-2831.
33. Novikov, V. V.; Pavlov, A. A.; Nelyubina, Y. V.; Boulon, M.-E.; Varzatskii, O. A.; Voloshin, Y. Z.; Winpenny, R. E. P., A Trigonal Prismatic Mononuclear Cobalt(II) Complex Showing Single-Molecule Magnet Behavior. *J. Am. Chem. Soc.* **2015,** *137* (31), 9792-9795.
34. Rigamonti, L.; Bridonneau, N.; Poneti, G.; Tesi, L.; Sorace, L.; Pinkowicz, D.; Jover, J.; Ruiz, E.; Sessoli, R.; Cornia, A., A Pseudo-Octahedral Cobalt(II) Complex with Bispyrazolylpyridine Ligands Acting as a Zero-Field Single-Molecule Magnet with Easy Axis Anisotropy. *Chem. Eur. J.* **2018,** *24* (35), 8857-8868.
35. Saber, M. R.; Singh, M. K.; Dunbar, K. R., Geometrical control of the magnetic anisotropy in six coordinate cobalt complexes. *Chem. Commun.* **2020,** *56* (60), 8492-8495.
36. Zhang, Y.-Z.; Gómez-Coca, S.; Brown, A. J.; Saber, M. R.; Zhang, X.; Dunbar, K. R., Trigonal antiprismatic Co(ii) single molecule magnets with large uniaxial anisotropies: importance of Raman and tunneling mechanisms. *Chem. Sci.* **2016,** *7* (10), 6519-6527.
37. Zhu, Y.-Y.; Cui, C.; Zhang, Y.-Q.; Jia, J.-H.; Guo, X.; Gao, C.; Qian, K.; Jiang, S.-D.; Wang, B.-W.; Wang, Z.-M.; Gao, S., Zero-field slow magnetic relaxation from single Co(ii) ion: a transition metal single-molecule magnet with high anisotropy barrier. *Chem. Sci.* **2013,** *4* (4), 1802-1806.
